# ATP2B4 driven chromatin compaction exacerbates pancreatic cancer radiotherapy resistance

**DOI:** 10.1038/s41420-026-03142-7

**Published:** 2026-05-25

**Authors:** Yuyu Luo, Wei Jiang, Yanfang Liu, Qinghua Li, Yuanfei Chen, Shiwan Lin, Hsiang-i Tsai, Lirong Zhang, Dongqing Wang, Xiang Liao, Haitao Zhu

**Affiliations:** 1 Institute of Medical Imaging and Artificial Intelligence, Jiangsu University, Zhenjiang, China; 2 School of Medicine, Jiangsu University, Zhenjiang, China; 3 Department of Medical Imaging, The Affiliated Hospital of Jiangsu University, Zhenjiang, China

**Keywords:** Cancer, Chromatin structure, Apoptosis, Proteomics, RNA sequencing

## Abstract

The intrinsic radio-resistance of pancreatic cancer cells significantly hinders therapeutic efficacy. However, the precise molecular mechanisms underlying this resistance remain inadequately understood and warrant further investigation. Here, using the high-throughput metabolic CRISPR library screening and RNA sequencing, we identified an ATPase Plasma Membrane Ca^2+^ Transporting 4 (ATP2B4) as a novel molecular contributor to radiotherapy resistance in pancreatic cancer both in vitro and in vivo. Functionally, micrococcal nuclease assay, drug rescue assays, along with overexpression and silencing experiments, revealed that knockout of ATP2B4 induced chromatin decompaction through the downregulation of histone H1.0, thereby exacerbating DNA damage and increasing RT-induced cell apoptosis. Mechanistically, TurboID-based mass spectrometry and immunoprecipitation (IP) demonstrated that ATP2B4 stabilized ELAVL1, maintaining its function, which further regulated the mRNA stability of histone H1.0. Taken together, our findings identified ATP2B4 as a key regulator of chromatin compaction and DNA damage response, positioning it as a potential biomarker for predicting RT outcomes and a promising therapeutic target for overcoming RTR.

## Introduction

Radiotherapy (RT) is utilized in over 90% of pancreatic ductal adenocarcinoma (PDAC) cases for curative, palliative, or adjuvant purposes [1, 2]. Although RT effectively inhibits tumor growth during the initial stages of treatment, increasing clinical evidence suggests that tumor recurrence or even accelerated progression often follows RT. This phenomenon is primarily attributed to the intrinsic radio-resistance of pancreatic cancer cells [3]. Consequently, the molecular mechanisms underlying this radio-resistance require further investigation.

The efficacy of RT in killing cancer cells critically depends on the induction of DNA damage [4]. The extent of DNA damage is determined by the balance between DNA susceptibility and the cell’s ability to repair such damage, a process influenced by multiple factors. Among these, chromatin compaction plays a pivotal role in both DNA vulnerability and DNA repair [5]. Chromatin compaction is a dynamic process involving both compaction and decompaction. On one hand, decompacted chromatin facilitates the recruitment of repair proteins, thereby promoting DNA repair [6, 7]. On the other hand, chromatin decompaction may render the DNA more susceptible to damage induced by persistent oxidative stress from RT (e.g., ROS) [8–11]. Conversely, highly compacted chromatin, such as heterochromatin, may protect against harmful factors while simultaneously hindering the repair process [12–15]. Overall, chromatin compaction plays a dual role in both DNA vulnerability and repair, suggesting that precise regulation of chromatin compaction could serve as a strategy for radio-sensitization. However, this potential remains incompletely understood and warrants further exploration.

The H1 histone family, comprising 11 variants, is directly involved in chromatin compaction and heterochromatin formation [16, 17]. Histone H1 is extensively implicated in DNA damage, apoptosis, chromatin decompaction, and the inhibition of DNA repair protein recruitment [13–15, 18–22]. Among the H1 family, H1.0 is the most conserved variant and serves as a strong condenser. H1.0 has been shown to mitigate chemotherapy resistance in ovarian cancer and promote autophagic cell death in hepatocellular carcinoma [16, 23–25]. However, its precise role in DNA damage associated with chemo/radiotherapy remains unclear and warrants further investigation. This study explores whether H1.0 mediates DNA damage response in pancreatic cancer by modulating chromatin compaction.

Using a metabolism-related CRISPR-Cas9 library and transcriptomic sequencing, this study identified ATPase Plasma Membrane Ca^2+^ Transporting 4(ATP2B4) as a key mediator of resistance to DNA damage and apoptosis in pancreatic cancer. Silencing ATP2B4 decreased H1.0 histone mRNA and protein levels, leading to enhanced chromatin decompaction. Turbo-ID analysis revealed that ATP2B4 affected H1.0 mRNA stability through ELAVL1. The deficiency of H1.0 resulted in chromatin decompaction and reduced heterochromatin, thereby sensitizing the cells to DNA damage and apoptosis induced by RT and chemotherapy.

## Results

### ATP2B4 was involved in RT resistant

**Fig. 1:**
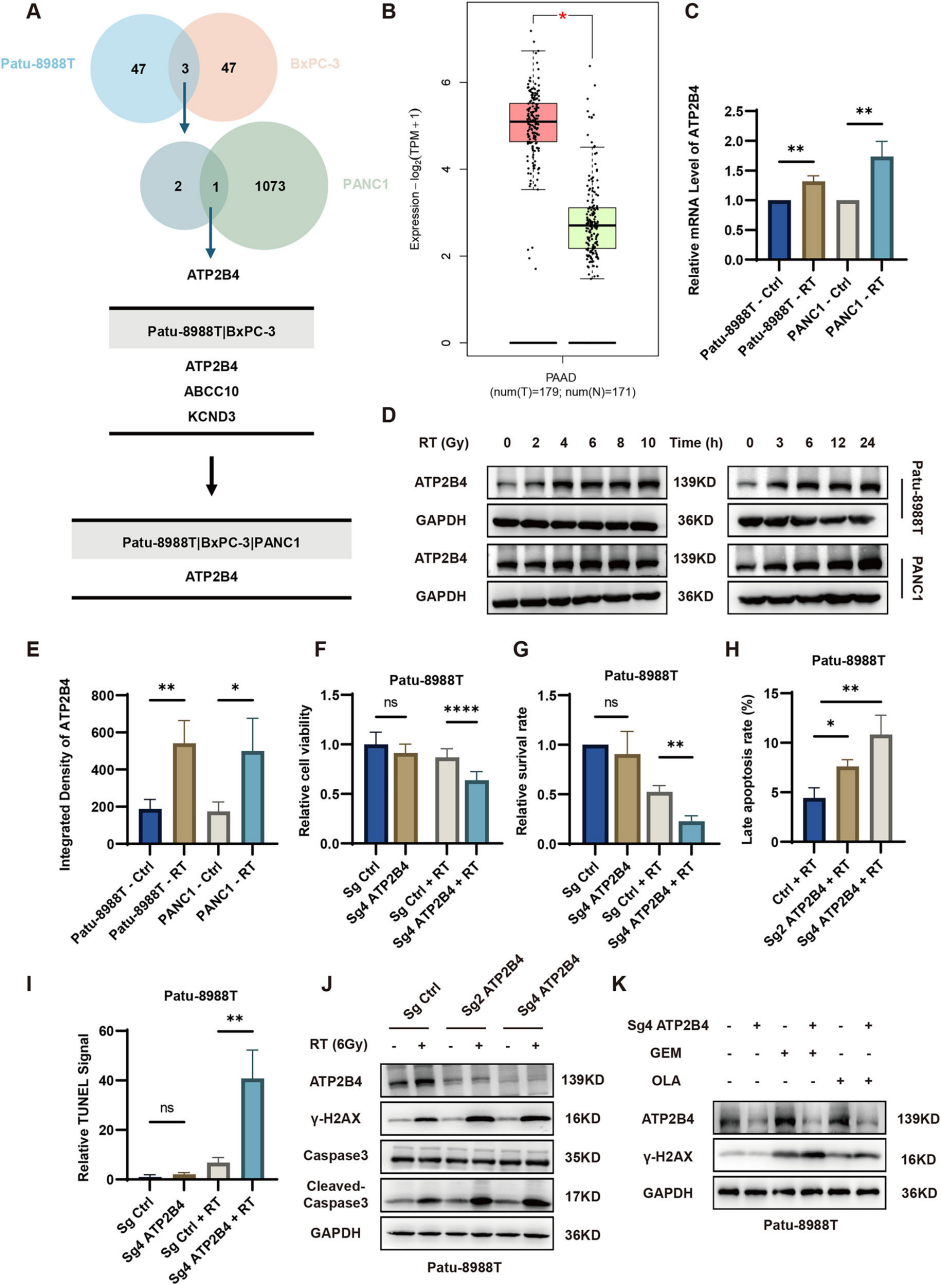
ATP2B4 was screened as an essential gene mediating RTR in pancreatic cancer. **A** Metabolic in vitro CRISPR screen aimed at identifying top 50 regulators of radiotherapy in Patu-8988T cells and BXPC-3 cells, RNA-seq identified up-regulated genes in PANC1 cells treated with a radiation dose of 6 Gy, ATP2B4 was screened by Veen map. **B** The expression of ATP2B4 in pancreatic cancer and adjacent tissues was plotted, data were obtained from TCGA database. **C** qPCR analysis of ATP2B4 mRNA expression in Patu-8988T and PANC1 cells 24 h after treatment with or without radiotherapy (6 Gy), *n* = 3. **D** Western blot of the protein expression of ATP2B4 with time and radiation dose after radiotherapy in Patu-8988T and PANC1 cells. **E** Quantification of Cell fluorescence which used to detect the expression of ATP2B4 in Patu-8988T and PANC1 cells 24 h after treatment with or without RT (6 Gy), *n* = 3. **F** Cell viability of Patu-8988T Ctrl and ATP2B4 KO cells were evaluated 24 h after treatment with or without RT (6 Gy), *n* = 9. **G** Quantification of clonogenic survival analysis of Patu-8988T cells, which were cultured for 2 weeks after treated with or without RT (4 Gy), *n* = 3. **H** Flow cytometry was used to measure apoptosis progression in Ctrl and ATP2B4 KO cells 24 h after treatment with or without RT (6 Gy) in Patu-8988T and PANC1 cells, proportion of late apoptosis cells was plotted. *n* = 3. **I** Quantification of tunel assay analysis in Patu-8988T cells 24 h after treatment with or without RT (6 Gy), *n* = 3. **J** Western blot of ATP2B4, γ-h2ax, Caspase3 and cleaved-caspase3 protein of Ctrl and ATP2B4 KO cells, Patu-8988T cells were cultured for 24 h after treated with RT (6 Gy) or not. **K** Western blot of ATP2B4 and γ-h2ax protein of Patu-8988T Ctrl and ATP2B4 KO cells treated with or without Gemcitabine (GEM, 10 μM) or Olaparib (OLA, 5 μM) for 24 hours. Data are presented as mean ± SD. *p < 0.05, **p < 0.01, ***p < 0.001, ****p < 0.0001.

Based on our previous metabolic CRISPR-Cas9 library results, the top 50 genes associated with radiotherapy resistance in human pancreatic cancer cells (Patu-8988T and BXPC3) were selected [26]. RNA sequencing identified 1074 upregulated genes following RT treatment in PANC1 cells. Among the two screening hits, ATPase Plasma Membrane Ca^2+^ Transporting 4(ATP2B4) consistently ranked highly (Fig. 1A). To validate the role of ATP2B4 in radiotherapy outcomes, data from the TCGA database revealing that ATP2B4 expression was significantly higher in tumors compared to peritumoral tissues, although no correlation was observed with patient overall survival (Fig. 1B and Fig. S1A). Treatment of Patu-8988T and PANC1 cells with varying doses of X-ray showed a radiation dose- and time-dependent upregulation of ATP2B4 protein and mRNA levels (Fig. 1C, D). Similar results were observed through immunofluorescence assays (Fig. 1E and Fig. S4J). Furthermore, in the Patu-8988T radioresistant (RR) cell line [27] (Fig. S1B), which demonstrated higher cell viability and lower γ-H2AX and P53 expression in response to RT, elevated ATP2B4 protein levels were also observed, which were further enhanced with RT treatment (Fig. S1D, E).

**Fig. 2:**
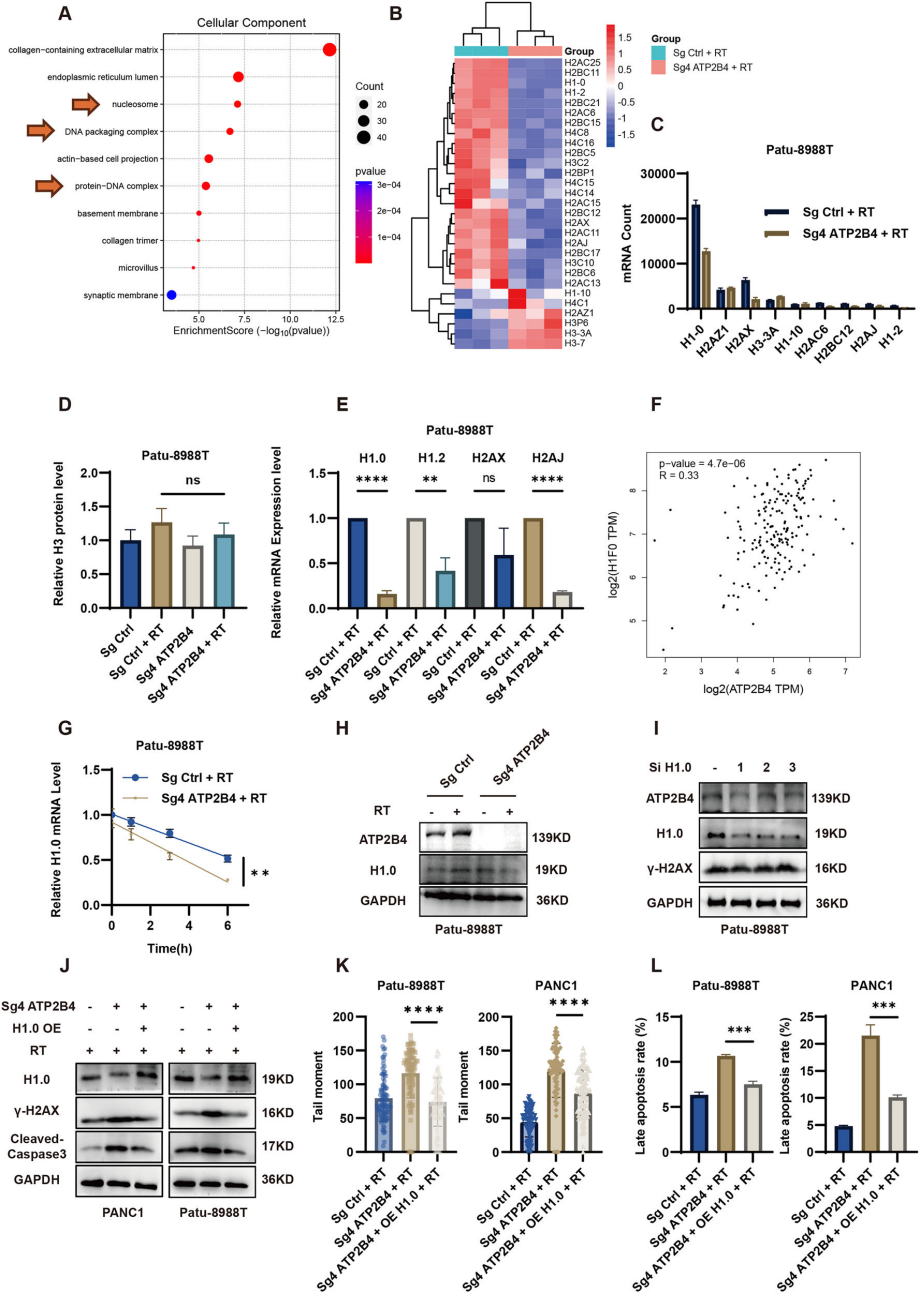
ATP2B4 mediates radiotherapy resistance in pancreatic cancer cells through H1.0. **A** Cellular components enrichment analysis of downregulated genes of ATP2B4 KO Patu8988T cells compared with Ctrl cells, Patu-8988T cells were cultured for 24 h after treated with RT (6 Gy). **B** Heatmap of histones which significantly changed in ATP2B4 KO cells compared with Ctrl cells, both were treated with RT (6 Gy). **C** mRNA changes in several histones from RNA-seq. **D** H3 protein levels of Ctrl and ATP2B4 KO cells treated with or without RT (6 Gy) in Patu-8988T cells were assessed by quantitative Western blotting. The intensity of Coomassie staining was used as loading control and normalization. All cells were cultured in 1% fetal bovine serum for 24 h to ensure cell synchronization, *n* = 3. **E** qPCR analysis of H1.0, H1.2, H2AX and H2AJ mRNA expression in Ctrl and ATP2B4 KO cells 24 h after treatment with RT (6 Gy), all cells were cultured in 1% fetal bovine serum for 24 h to ensure cell synchronization, *n* = 3. **F** The significant positive correlation between ATP2B4 and H1.0 mRNA expression was shown, data were obtained from TCGA database. **G** Stability of H1.0 mRNA was measured by qPCR, Patu-8988T Ctrl and ATP2B4 KO cells were cultured for 1 h, 3 h, 6 h with a 5 ug/ml concentration of Actinomycin D after treated with RT (6 Gy), *n* = 3. **H** Western blot of H1.0 and ATP2B4 protein in Patu-8988T Ctrl and ATP2B4 KO cells treated with or without RT (6 Gy). **I** Western blot of H1.0, ATP2B4 and γ-h2ax protein in Patu-8988T cells after treated with H1.0 siRNA for 48 h. **J** Western blot of H1.0, cleaved-caspase3 and γ-h2ax protein in Patu-8988T cells treated with RT (6 Gy). **K** The tail moment was measured and statistically analyzed, *n* > 80. **L** Flow cytometry was used to measure apoptosis progression of Patu-8988T and PANC1 cells 48 h after treatment with RT (6 Gy), proportion of late apoptosis cells was plotted, *n* = 3. Data are presented as mean ± SD. *p < 0.05, **p < 0.01, ***p < 0.001, ****p < 0.0001.

To further elucidate the role of ATP2B4 in RT resistance, ATP2B4 knockout cancer cells with 80% efficiency were established (Fig. S1C). While ATP2B4 knockout did not significantly affect cancer cell viability under normal conditions, it markedly sensitized cancer cells to RT-induced viability suppression (Fig. 1F and Fig. S1F). Colony-forming assays revealed that RT treatment significantly inhibited the proliferative capacity of ATP2B4 knockout cells compared to the ATP2B4 wide type cells (Fig. 1G and Fig. S1G). RT-induced cancer cell death relies on sustained oxidative stress-driven DNA damage and subsequent apoptosis [4, 28]. FACS analysis showed a significant increase in late apoptosis in RT-treated ATP2B4 knockout cells (Fig. 1H and Fig. S1H). TUNEL assays further demonstrated a substantial increase in DNA fragmentation in ATP2B4 knockout cells compared to wild-type cells, suggesting enhanced apoptosis upon ATP2B4 knockdown (Fig. 1I and Fig. S1I). Notably, γ-H2AX expression, a marker of DNA damage, and cleaved caspase-3 were unaffected in ATP2B4 knockout cells, but significantly elevated upon RT treatment (Fig. 1J and Fig. S1J). Additionally, RAD51 expression level were also upregulated, while DNA-PKcs remained stable, indicating that the synchronous enhancement of homologous recombination repair pathway after RT (Fig. S1K). Comet assays revealed a marked increase in tail moment in RT-treated ATP2B4 knockout cells (Fig. 2J and Fig. S2C), while similar Reactive Oxygen Species (ROS) level observed, further supporting increased DNA damage which was independent of ROS level (Fig. S1L). Additionally, treatment of ATP2B4 knockout cells with gemcitabine (GEM) and Olaparib (OLA), two well-established DNA-damaging chemotherapeutic agents, resulted in upregulation of γ-H2AX and increased late apoptosis (Fig. 1K). Collectively, these results demonstrate that ATP2B4 is involved in DNA damage-related resistance to cancer therapies.

### ATP2B4 mediated radio-resistance via H1.0

To investigate the mechanisms underlying ATP2B4-mediated resistance to DNA damage-based therapies, mRNA sequencing was performed on ATP2B4 knockout and wild-type Patu-8988T cells. Genes significantly downregulated (*P* < 0.05) were identified and subjected to Cellular Component (CC) enrichment analysis. Notably, nucleosome-related pathways ranked among the top ten enriched pathways (Fig. 2A). Heatmap analysis revealed significant downregulation of multiple histones, key components of the nucleosome, in the ATP2B4 knockout group (Fig. 2B). The mRNA counts from RNA-seq revealed the changes in the abundance of histones (Fig. 2C).

To clarify the role of histones in ATP2B4-regulated RT resistance, histone abundance and global nucleosome occupancy were first quantified, using H3 protein expression as a proxy, according to the previous publication [29]. ATP2B4 knockout did not affect H3 protein expression (Fig. 2D). Furthermore, no significant difference in H3 protein levels was observed between RT-treated ATP2B4 knockout and wild-type cancer cells, indicating that some specific histones were involved in ATP2B4-mediated RT resistance, rather than the global histones (Fig. 2D). To further identify the specific histones, four highly expressed candidates (H1.0, H1.2, H2AX, and H2AJ) were selected (Fig. S2A). Notably, only H1.0 expression levels, rather than those of H1.2, H2AX, or H2AJ, were strongly associated with ATP2B4 expression in the TCGA database (Fig. 2F and Fig. S2B). Moreover, ATP2B4 knockout led to the most significant downregulation of H1.0 at the mRNA level (Fig. 2E). To exclude potential cell cycle differences induced by ATP2B4 knockout, both wild-type and knockout cells were cultured under serum-deprived conditions to synchronize the cell cycle, and the association between ATP2B4 and H1.0 expression remained consistent (Fig. 2E). Actinomycin D chase assay revealed an increased degradation rate of H1.0 mRNA in ATP2B4 knockout cells, suggesting that the reduction in H1.0 was due to decreased mRNA stability rather than transcriptional regulation (Fig. 2G). Additionally, while H1.0 protein levels remained unchanged in response to RT treatment, they were significantly downregulated in RT-treated ATP2B4 knockout cells (Fig. 2H), with similar results observed following GEM and OLA treatment (Fig. S2C). Furthermore, RT upregulated H1.0 protein levels in RR cells, while GEM treatment increased H1.0 levels in both Patu-8988T and RR cells (Fig. S2D).

**Fig. 3:**
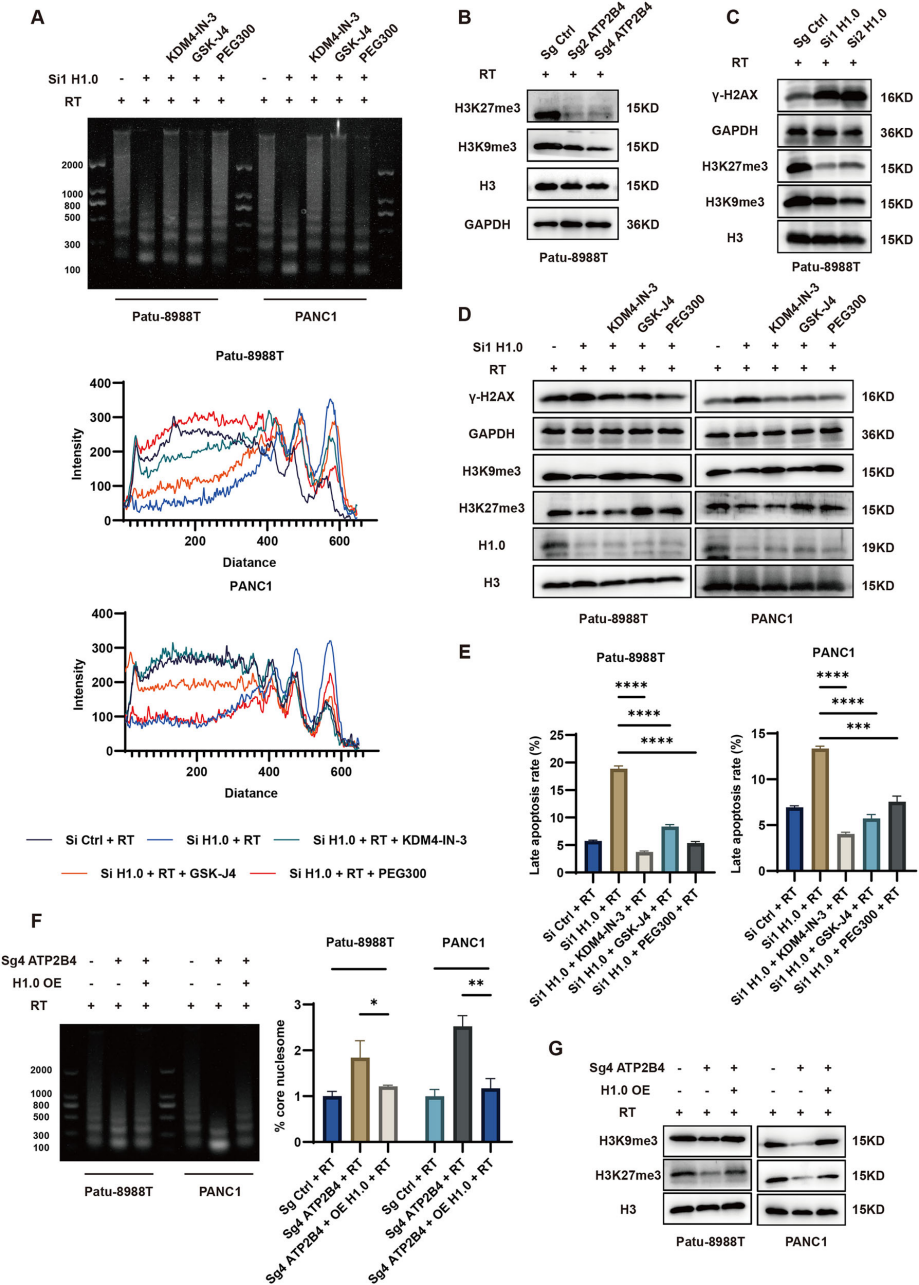
H1.0 mediates radioresistance by affecting chromatin compaction. **A** MNase digestion was used to detect the chromosome compaction degree of Ctrl, H1.0 knockdown cells treated with chromatin compaction inducers and RT (6 Gy), the intensity of each lane was quantified. Drug concentration: GSK-J4 (1 μM), KDM-IN-3 (1 μM), PEG300 (80 mM). **B** Western blot of H3K9me3 and H3K27me3 protein in Ctrl and ATP2B4 KO Patu-8988T cells treated with RT (6 Gy). **C** Western blot of γ-h2ax, H3K9me3, and H3K27me3 protein in Ctrl and H1.0 knockdown Patu-8988T cells treated with RT (6 Gy). **D** Western blot of γ-h2ax, H1.0, H3K9me3, and H3K27me3 protein of Ctrl, H1.0 knockdown cells treated with chromatin compaction inducers and RT (6 Gy). **E** Flow cytometry was used to measure apoptosis progression of Ctrl, H1.0 knockdown cells treated with chromatin compaction inducers and RT (6 Gy), proportion of late apoptosis cells was plotted, *n* = 3. **F** MNase digestion was used to detect chromosome compaction of Ctrl, ATP2B4 KO, and H1.0 overexpression based on ATP2B4 KO cells treated with RT. with the percentage of core nucleosome (approximately 100-200-bp fragment) quantified, *n* = 3. **G** Western blot of H3K9me3 and H3K27me3 protein of Ctrl, ATP2B4 KO and H1.0 overexpression based on ATP2B4 KO cells treated with RT (6 Gy). Data are presented as mean ± SD. *p < 0.05, **p < 0.01, ***p < 0.001, ****p < 0.0001.

Next, the role of H1.0 in radiotherapy sensitivity was assessed using established H1.0 knockdown cancer cells. H1.0 knockdown did not affect ATP2B4 expression, confirming that ATP2B4 is upstream of H1.0. And the mRNA expression of other H1 family members were not significantly affected by the knockdown of H1.0 (Fig. S2E). γ-H2AX levels remained unchanged in H1.0 knockdown cells, but increased significantly upon RT treatment, indicating that H1.0 deficiency does not impact DNA damage but affects RT therapy sensitivity (Fig. 2I and Fig. 3F). To further confirm that H1.0 mediates ATP2B4-driven RT resistance, H1.0 was overexpressed in ATP2B4 knockout cells. Overexpression of H1.0 reversed the upregulation of γ-H2AX and cleaved caspase-3, as well as the increased tail moment observed in RT-treated ATP2B4 knockout cells (Fig. 2J, K and Fig. S2F). The higher percentage of late apoptosis in RT- or GEM-treated ATP2B4 knockout cells was also restored in H1.0 overexpressing cells (Fig. 2L and Fig. S2G, H). Collectively, these results demonstrate that H1.0 plays a key role in ATP2B4-mediated RT resistance.

### H1.0 compacted chromatin structure enhances ATP2B4-mediated RT resistance

Chromatin compaction, tightly regulated by H1.0, plays a pivotal role in DNA damage-related cancer therapies. To determine whether H1.0-mediated chromatin compaction underlies ATP2B4-induced RT resistance, the global chromatin compaction level was assessed using the Micrococcal nuclease (MNase) digestion assay, as previously described [30]. MNase digestion revealed chromatin decompaction in H1.0 knockdown cancer cell (Fig. 3A). Additionally, protein levels of H3K9me3 and H3K27me3, markers of heterochromatin, were downregulated in both H1.0 knockdown and ATP2B4 knockout cancer cells induced by RT (Fig. 3B, C and Fig S3B).

To further confirm the role of H1.0-induced chromatin compaction in RT resistance, chromatin compaction inducers (GSK-J4, KDM-IN-3, and PEG300) were utilized [31–33]. At non-toxic concentrations—1 µM GSK-J4, 10 µM KDM-IN-3, and 80 mM PEG300—these inducers were applied in subsequent experiments (Fig. S3A). MNase digestion assays demonstrated that chromatin decompaction in RT-treated H1.0 knockdown cells could be partially restored by the chromatin compaction inducers (Fig. 3A). Furthermore, the downregulation of H3K9me3 and H3K27me3, along with increased γ-H2AX levels and a higher late apoptosis ratio in RT-treated H1.0 knockdown cells, was reversed upon treatment with the chromatin compaction inducers (Fig. 3D, E and Fig. S3C). These results confirmed that chromatin compaction is involved in H1.0-mediated RT resistance, and the expression level of H1.0 can directly regulate chromatin compaction level.

To explore whether H1.0-mediated chromatin compaction also underpins ATP2B4-induced RT resistance, chromatin compaction was examined in RT-treated ATP2B4 knockout cells. The signal intensity of mononucleosomes in RT-treated ATP2B4 knockout cells was higher compared to RT-treated wild-type cells, a finding reversed by overexpression of H1.0 in ATP2B4 knockout cells, suggesting that ATP2B4 knockout reduces chromatin compaction and H1.0 overexpression restores it (Fig. 3F). Protein levels of H3K9me3 and H3K27me3 were significantly reduced in RT-treated ATP2B4 knockout cells (Fig. 3G). Overexpression of H1.0 in ATP2B4 knockout cells partially restored the reduced levels of H3K9me3 and H3K27me3 following RT treatment (Fig. 3G). Collectively, these results demonstrate that H1.0-mediated chromatin compaction is integral to ATP2B4-driven RT resistance.

### ATP2B4 maintains H1.0 mRNA via ELAVL1

To investigate the relationship between ATP2B4 and H1.0, it is important to note that ATP2B4 functions through the extrusion of intracellular Ca^2+^. KEGG enrichment analysis revealed significant enrichment of the calcium signaling pathway based on downregulated gene sets in ATP2B4 knockout cancer cells (Fig. S4A). Interestingly, treatment with BAPTA, a specific Ca^2+^ chelator used to maintain low intracellular Ca^2+^ levels, did not significantly affect the protein levels of H3, H1.0, or γ-H2AX, nor the late apoptosis rate in either ATP2B4 knockout or wild-type cancer cells (Fig. S4C–E). Similarly, treatment with ATA, an ATP2B4 Ca^2+^ pump inhibitor that increases intracellular Ca^2+^ concentration, resulted in comparable outcomes, suggesting that ATP2B4-mediated Ca^2+^ efflux does not significantly influenced RT resistance (Fig. S4B–E). Immunofluorescence further confirmed that ATP2B4 predominantly localized to the cytoplasm following RT treatment (Fig. S4J). These findings suggest that ATP2B4 regulates H1.0 *via* a non-canonical pathway.

**Fig. 4:**
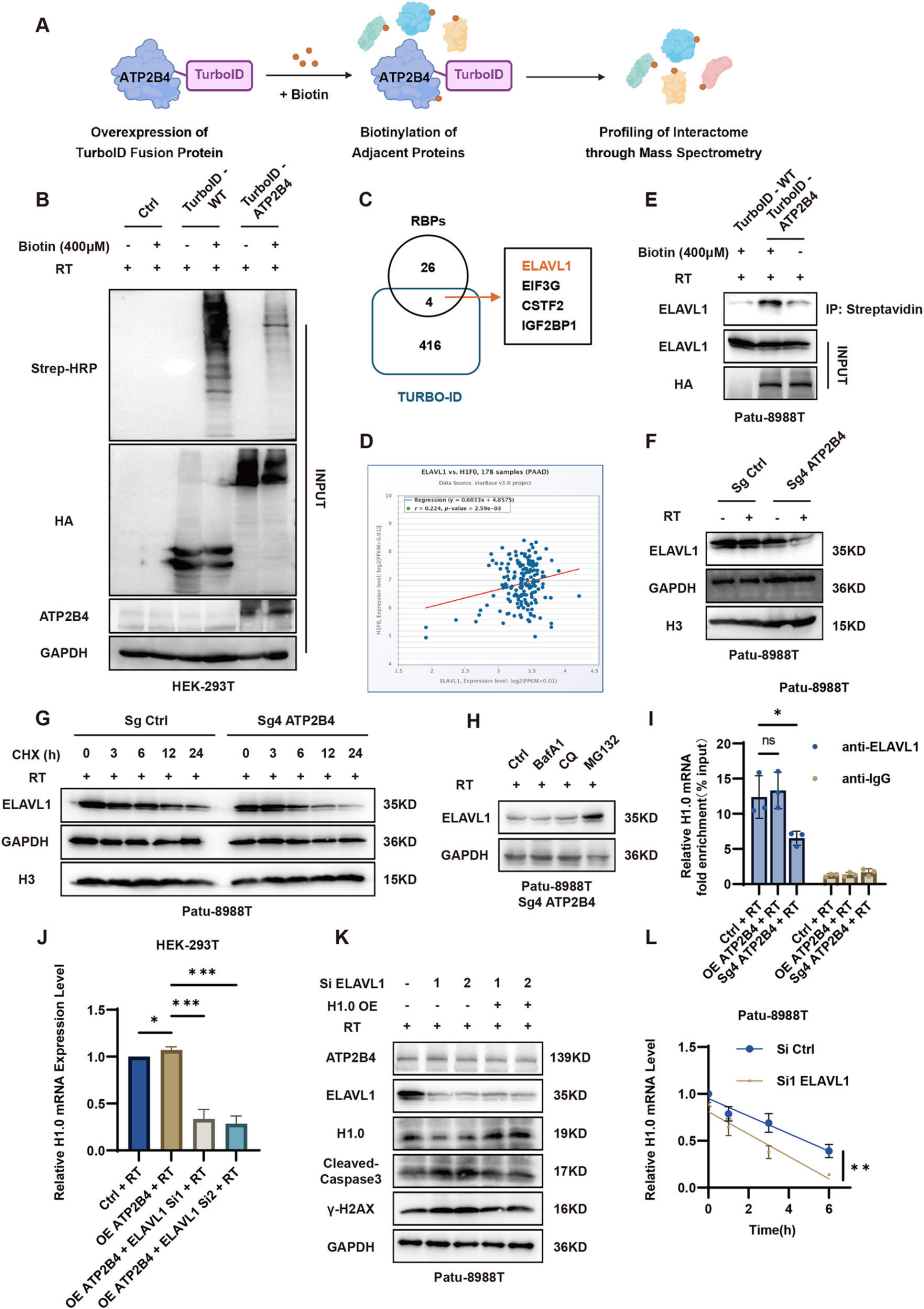
ATP2B4 regulates the stability of H1.0 mRNA through ELAVLA. **A** The pattern diagram shows the construction principle of the Turbo-ID system. The TurboID fusion protein was overexpressed and labeled with biotin, adjacent possible interacting proteins were also labeled and enriched using streptavidin magnetic beads. The enriched proteins were identified by mass spectrometry. **B** plv-HA-ATP2B4-TurboID plasmids were transfected in the HEK293 cells, biotinylated proteins were shown by Western blot analysis. **C** RBPs bind to the H1.0 mRNA obtained by POSTAR3 (http://111.198.139.65/) and biotinylated proteins were identified by mass spectrometry (Table S2), Veen diagram showed 4 candidates. **D** The significant positive correlation between the expression of ELAVL1 and H1.0 mRNA was shown, data was obtained from Starbase v3.0. **E** Immunoprecipitation was performed on the lysates of Patu-8988T cells expressing ELAVL1 protein using ELAVL1 or mouse IgG homologous antibodies. **F** Western blot of ELAVL1 protein in Ctrl and ATP2B4 KO Patu-8988T cells treated with or without RT (6 Gy). **G** ELAVL1 protein stability was measured by Western blot after Patu-8988T cells were exposed to CHX (50 ug/mL) after treatment with RT (6 Gy). **H** Western blot of ELAVL1 protein in Patu-8988T ATP2B4 KO cells treated with drugs that inhibit different protein degradation pathways for 12 h after treatment with RT (6 Gy), Drug concentration: BafA1 (1 μM), CQ (20 μM), MG132 (50 μM). **I** RIP assays were used to detect the H1.0 mRNA bound to ELAVL1 in Ctrl, ATP2B4 overexpression and ATP2B4 KO Patu-8988T cells with treatment of RT (6 Gy), *n* = 3. **J** H1.0 mRNA was detected in Ctrl, ATP2B4 overexpression, and ELAVL1 knockdown based on ATP2B4 overexpression cells, *n* = 3. **K** Western blot of H1.0, ATP2B4, ELAVL1, cleaved-caspase3, and γ-h2ax protein levels in Patu-8988T cells treated with RT (6 Gy). **L** Stability of H1.0 mRNA was measured by qPCR, Patu-8988T Ctrl and ELAVL1 knockdown cells were cultured for 1 h, 3 h, 6 h with a 5 ug/ml concentration of Actinomycin D after treated with RT (6 Gy), *n* = 3. Data are presented as mean ± SD. *p < 0.05, **p < 0.01, ***p < 0.001.

To further investigate the regulatory relationship between ATP2B4 and H1.0, Turbo-ID technology was employed for proximity labeling proteomics to identify ATP2B4 interactors (Fig. 4A) [34], resulting in the identification of 420 potential ATP2B4-associated proteins *via* mass spectrometry (Fig. 4B). Additionally, 30 RNA-binding proteins (RBPs) associated with H1.0 mRNA were identified from the POSTAR3 database (Table S2). A Venn diagram was used to screen four key proteins, with ELAVL1 emerging as a significant hit, widely known for its role in RNA stability (Fig. 4C). Starbase V3.0 confirmed a positive correlation between ELAVL1 expression and H1.0 mRNA levels (Fig. 4D). IP confirmed the interaction between ATP2B4 and ELAVL1 (Fig. 4E). Notably, the downregulation of ELAVL1 was observed at the protein level in both the nucleus and the cytoplasm, but not at the mRNA level, in ATP2B4 knockout Patu-8988T cells upon RT treatment (Fig. 4F and Fig. S4G, H). CHX chase assays revealed accelerated degradation of ELAVL1 in ATP2B4 knockout Patu-8988T cells (Fig. 4G). Further experiments revealed that the ubiquitination level of ELAVL1 was higher in the knockout group (Fig. S4I). To elucidate the mechanism by which ATP2B4 stabilizes ELAVL1, ATP2B4 knockout cancer cells were treated with BafA1 (an autophagy inhibitor), chloroquine (CQ, a lysosomal inhibitor), and MG132 (a proteasome inhibitor). Only MG132, but not BafA1 or CQ, restored ELAVL1 levels in ATP2B4 knockout cells, indicating that ATP2B4 stabilizes ELAVL1 by inhibiting proteasomal degradation (Fig. 4H).

Further investigation into the role of ELAVL1 in ATP2B4-mediated H1.0 downregulation was conducted by knocking down ELAVL1 in wild-type and ATP2B4 overexpressing cancer cells. ELAVL1 knockdown did not affect ATP2B4 protein levels but led to downregulation of H1.0, confirming that ELAVL1 is downstream of ATP2B4 and upstream of H1.0 (Fig. 4L). RNA IP (RIP) assays revealed that the binding between ELAVL1 and H1.0 mRNA was significantly reduced in ATP2B4 knockout cells, whereas ATP2B4 overexpression had a minimal effect on H1.0 mRNA binding by ELAVL1 (Fig. 4I and Fig. S4K). Furthermore, the mRNA upregulation of H1.0 induced by ATP2B4 overexpression was significantly reversed upon ELAVL1 knockdown (Fig. 4J). ELAVL1 knockdown resulted in increased γ-H2AX and cleaved caspase-3 levels, which were reversed upon H1.0 overexpression (Fig. 4K). Moreover, H1.0 mRNA degradation was also accelerated in ELAVL1 knockdown Patu-8988T cells, further supporting the role of ELAVL1 in stabilizing H1.0 mRNA (Fig. 4L). Notably, the addition or deprivation of Ca^2+^ did not affect ELAVL1 protein levels (Fig. S4F). Collectively, these results indicate that ATP2B4 regulates H1.0 mRNA stability *via* ELAVL1, independent of global change of concentration of Ca^2+^.

### ATP2B4-ELAVL1-H1.0 axis drives RT resistance in pancreatic cancer

**Fig. 5:**
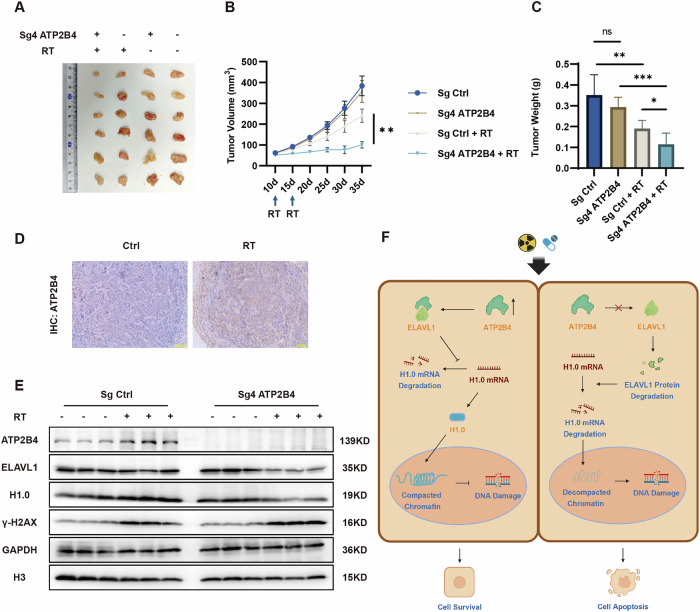
The ATP2B4/ELAVL1/H1.0 signaling axis plays an important role in the treatment resistance of pancreatic cancer. **A** BXPC3 cells infected with lentivirus were injected subcutaneously into the right flanks of Balb/c nude mice, mice were treated with RT (6 Gy, twice), and the xenograft tumors were removed 35 days after injection. **B** Tumor growth curve was performed with length × width^2^ × 1/2 of the tumor measured every 5 days. **C** The weight of xenograft tumor. **D** Representative images of IHC, the expression of ATP2B4 was determined. **E** The expression level of ELAVL1, ATP2B4, γ-H2AX, and H1.0 protein in xenograft tumors were determined by Western blot. **F** A proposed modulatory model for the function of ATP2B4-ELAVL1-H1.0 axis in pancreatic cancer for treatment resistance. *p < 0.05, **p < 0.01.

To further validate our in vitro findings, a nude mouse model bearing BxPC3 subcutaneous tumors was established and randomly divided into the following treatment groups: Sg Ctrl, Sg Ctrl + RT, Sg4 ATP2B4, and Sg4 ATP2B4 + RT. Tumor growth, volume, and weight were significantly inhibited in the Sg Ctrl + RT and Sg4 ATP2B4 + RT groups compared to controls, with no significant differences observed in the Sg4 ATP2B4 group. Additionally, tumor growth, volume, and weight were notably reduced in the Sg4 ATP2B4 + RT group compared to the Sg Ctrl + RT group. In terms of protein expression, ATP2B4 and γ-H2AX levels were upregulated, while ELAVL1 and H1.0 expression remained unchanged in the RT treatment group (Fig. 5A–D). Furthermore, in the RT + ATP2B4 knockout group, ATP2B4, ELAVL1, and H1.0 expression levels were downregulated, while γ-H2AX expression was upregulated compared to the RT treatment group (Fig. 5E).

## Discussion

DNA damage response plays a pivotal role in determining the efficacy of radiotherapy and is regulated by various factors, including chromatin structure and function. In this study, CRISPR-Cas9 metabolic screening and RNA sequencing identified ATP2B4 as an indirect chromatin compaction inducer—distinct from its classical role as a calcium pump—that alleviates DNA damage and cancer cell apoptosis, thereby promoting radiotherapy resistance. Additionally, knockout of ATP2B4 mediates chromatin decompaction through the downregulation of H1.0 at both the mRNA and protein levels. Moreover, ELAVL1, which escapes proteasomal degradation in the presence of ATP2B4, serves as a key bridge that maintains ATP2B4-induced H1.0 expression. Thus, targeting the ATP2B4-ELAVL1-H1.0 axis could potentiate DNA damage response, representing a novel strategy to enhance the efficacy of radiotherapy and DNA-damaging chemotherapy.

ATP2B4, a calcium pump predominantly located on the cell membrane, is responsible for exporting calcium to the extracellular space. Radiotherapy has been shown to cause excessive elevation of intracellular Ca^2+^ due to cell membrane damage, leading to calcium overload [35–38]. As oxidative stress gradually returns to baseline after radiotherapy, some of the excess Ca^2+^ is transported out of the cell. siRNA-mediated silencing of ATP2B4 has been reported to sensitize pancreatic cancer cells to various apoptosis inducers, a phenomenon linked to its classical function [39]. Similar findings have been observed in gastric cancer and glioma, although further validation is needed to confirm that calcium efflux is the primary functional form of ATP2B4 in resisting apoptosis [40, 41]. Our study discovered that ATP2B4 regulates the mRNA levels of histone H1.0 to enhance DNA damage and apoptosis through a non-classical mechanism, which is also dependent of global intracellular Ca²⁺ level change. And the dynamic changes in intracellular calcium levels following radiotherapy and their potential role in radiosensitization require further investigation. Both intracellular calcium regulation and ATP2B4’s role in radioresistance are likely to be crucial, warranting additional evidence to fully elucidate their contributions.

RBPs are key regulators of post-transcriptional processes, influencing RNA localization, translation, and stability. ELAVL1 (HuR), one of the most widely studied RBPs, is expressed in all human tissues and has been implicated in tumor cell death resistance and radiation resistance [42]. The present study identified a direct regulatory role for ELAVL1 in stabilizing H1.0 mRNA, providing insight into one of the mechanisms by which ELAVL1 mediates radiation resistance. Moreover, ATP2B4 directly affects ELAVL1 protein stability and function. However, the precise spatial interaction between ELAVL1 and ATP2B4, as well as how this interaction influences ELAVL1 binding to H1.0 mRNA, remains to be explored. Understanding these interactions will provide valuable insights for designing targeted therapies aimed at modulating this axis.

The histone H1 family plays a pivotal role in connecting nucleosomes within chromatin, operating in a distinct manner. Reduced H1 protein levels sensitize cells to DNA damage induced by radiotherapy and chemotherapy, however, they also facilitate the recruitment of repair proteins to promote DNA damage repair [7, 12, 14, 21, 43]. Despite these insights, the regulatory mechanisms governing H1 mRNA stability have not been fully explored [44]. The present study identified ELAVL1 as a key direct regulator that maintains the stability of H1.0 mRNA, thus influencing both H1.0 mRNA and protein levels.

A direct relationship between chromatin compaction and several members of the histone H1 family has been established [45–47]. While the interplay between chromatin compaction and DNA damage remains complex [48], our findings indicate that chromatin decompaction, induced by sustained low levels of H1.0 protein, sensitizes cells to radiotherapy. To further investigate this, several drugs that directly or indirectly induce chromatin decompaction were selected for validation. Although further evidence is required for broader application, these drugs demonstrated potential in promoting chromatin decompaction in this study, independent of H1.0 protein.

In conclusion, ATP2B4 was identified as a key molecule in enhancing the DNA damage response, suggesting its potential as a biomarker for predicting the efficacy of radiotherapy and DNA-damaging chemotherapy (Fig. 5F). Moreover, inhibition of the ATP2B4-ELAVL1-H1.0 axis presents a promising strategy to enhance the effectiveness of radiotherapy and DNA-damage-based chemotherapies.

## Materials and methods

### Reagents and plasmids

BAPTA (HY-100168), Aurintricarboxylic acid (ATA, HY-122575), PEG300 (HY-Y0873), KDM4-IN-3 (HY-132896), GSK-J4 (HY-15648B), Olaparid (HY-10162), Gemcitabine (HY-17026) were purchased from Med Chem Express (MCE, USA), lentiCRISPRv2 lentiviral vector was used to knock out ATP2B4, PCDH-CMV lentiviral vector for H1F0 and ATP2B4 overexpression. plv-HA-empty-TurboID or plv-HA-ATP2B4-TurboID plasmids were transfected in the HEK293 cells to identify biotinylated proteins, Relative sequences are listed in Table S1.

### Cell culture

Human pancreatic cancer cells, Patu-8988T, BXPC-3, PANC1 and human embryonic kidney cells, HEK293T were purchased from the Cell Bank of the China Academy of Sciences (Shanghai, China). All the cells were authenticated and tested for mycoplasma contamination. Cells were cultured in high-glucose Dulbecco’s Modified Eagle’s medium (DMEM, NanJing KeyGen Biotech, China) that contained 10% fetal bovine serum (FBS, ExCell biotech, China) and 1%(100 U/mL) penicillin-streptomycin (Gibco, USA) at 37 °C in a humidified incubator that contained 5% CO2.

### Cell viability and colony formation assay

Cell viability was measured by Cell Counting Kit-8 kit (CCK-8, Beyotime). Briefly, cells were plated in a 96-well plate at a density of 5×10^3^ per well. After treatment according to the expected experimental formula, the original medium was discarded with 90 µl fresh medium and 10 µl CCK-8 solution added and incubated for 1-4 h at 37 °C. The absorbance at 450 nm was measured using a microplate reader.

For colony formation assay, each group was removed from 1×10^3^ cells in a six-well plate and cultured in complete DMEM medium which changed every 3 days. After 14 days, the colonies were washed with PBS before adding 4% paraformaldehyde (Beyotime, China) and stained with crystal violet solution (Beyotime, China).

### Lentiviral collection and establishment of stable cell lines

To produce lentivirus against or overexpression specific gene, recombinant packaging plasmids were co-transfected into HEK 293 T cells. Culture supernatants that contained the virus were collected 24 and 48 h after transfection. To establish respective cell lines, Patu-8988t and PANC1 cells were cultured with the lentiviral solution for 48 h in the presence of 1 μg/mL polybrene (Sigma–Aldrich, St. Louis, MO, USA), followed by 2 µg/ml puromycin for 48 h. All stable cell lines were long term cultured in medium containing 0.5 µg/ml puromycin.

### RT-PCR analysis

Total RNA from cells using FastPure Cell/Tissue Total RNA Isolation Kit (Vazyme Biotech, China) and reverse transcribed to cDNA using HiScript® III RT SuperMix for qPCR (Vazyme Biotech, China), mRNA expressions was analyzed by quantitative real-time polymerase chain reaction assay (qRT-PCR) using 2 × SYBR Green qPCR Master Mix (TransGen Biotech, China). Respective primers are listed in Table S1.

### Western blot analysis

The protein fractions were separated using a polyacrylamide gel (SDS-PAGE) and then transferred to NC membranes. Membranes were blocked with 5% BSA for 1 h at room temperature and incubated with the desired primary antibodies overnight at 4 °C. After 1 h incubation at room temperature with an HRP-coupled secondary antibody, Membranes were washed three times with TBST buffer, the specific proteins were detected using an ECL reagent (Protein Tech, China). ImageJ (v. 1.8.0, Bethesda, MD, USA) was used for normalized quantification of the western blot. The primary antibodies used were as follows: Anti-γ-H2AX (1:1000; AF3187, Affinity, USA); Anti-ATP2B4 (1:1000; DF13326, Affinity, USA); Anti-FGF1 (1:1000; 17400-1-AP, Proteintech, China); Anti-caspase3 (1:1000; 9662S, Cell Signaling, USA); Anti-GAPDH (1:1000; AF7021, Affinity, USA); Anti-H3 (1:1000; BF9211, Affinity, USA); Anti-H3K9Me3 (1:1000; DF6938, Affinity, USA); Anti-H3K27Me3 (1:1000; DF6941, Affinity, USA); Anti-ELAVL1 (1:1000; BF8063, Affinity, USA); Anti-H1.0 (1:1000; AF0359, Affinity, USA); Anti-Tubulin (1:1000; AF7011, Affinity, USA).

### Immunofluorescence staining

After the cells were seeded into 24-well plates and treated accordingly, they were fixed in 4% paraformaldehyde for 15 min, followed by permeabilization with 0.3% Triton X-100 for 5 min and blocked in PBST containing 3% BSA for 1 h at room temperature. The cells were incubated with primary antibodies overnight at 4 °C. Followed by incubation with Alexa Fluor 594 or Alexa Fluor 488 conjugated secondary antibody for 1 hour. The nuclei were stained with DAPI. After PBST washing, cells were observed and monitored using a fluorescence microscope (Leica, USA) or confocal microscopy (ZEISS, Jerman).

### Comet electrophoresis

The alkaline comet assay was performed according to Cell damage agent kit (KeyGEN BioTECH, K231211). Cells were resuspended in 0.5% low-melting-point agarose and deposited on a microscope slide pre-coated with a thin layer of 1% agarose. The slides were incubated in lysis buffer for 1 h at 4°C and then in alkaline electrophoresis buffer (1 mM EDTA, 300 mM NaOH) for 30 min. Electrophoresis was performed at 25 V for 30 min. After electrophoresis, slide was incubated in 0.4 M Tris, pH 7.5 for 5 min. DNA staining of each slide was performed by overlaying 20 μl of PI. Comets were imaged by fluorescence microscope and analyzed with CaspLab software to calculate the tail moment, which was used to quantify the DNA damage degree. Each group contains at least 80 comets.

### Measurement of apoptosis

Terminal deoxynucleotidyl transferase-mediated dUTP-biotin nick end labeling (TUNEL) method was used to measure apoptosis rate according to the instructions of the manufacturer (Beyotime, C1086). Three low-power fields were randomly selected to calculate the proportion of apoptotic cells. Patu-8988T or PANC1 cells were seeded into 6-well plates at a density of 1.0 × 10^5^ cells/well. After the cells were treated accordingly. They were collected as single cells and stained with Annexin V-FITC/PI for 5 min. Flow cytometry was used to analyze apoptotic cells, and apoptosis rates of cells were analyzed by FlowJo 10.8.1.

### Micrococcal nuclease assay

After washing with cold PBS, 1 × 10^6^ cell were collected and resuspended in hypotonic buffer (10 mM HEPES [pH 7.9], 10 mM KCl, 1.5 mM MgCl2, 0.34 M sucrose, 10% glycerol, 1 mM dithiothreitol, and 0.1% Triton X-100). Nuclei were washed once with the hypotonic buffer without Triton X-100, 2 U micrococcal nuclease (MNase) (Beyotime, D7201S) with the presence of 5 mM CaCl2 was used to digested chromatin, after 5 minutes in a 37°C water bath and then added with 0.5 mM EFTA to stop the reaction. After digestion, 1 mg/ml Proteinase K and 0.5 mg/ml RNase A were added and then incubated at 56°C for 10 minutes to digest DNA-associated proteins and RNA. DNA fragments of different lengths were separated by gel electrophoresis, and percent core nucleosome is the percent integrated area of single nucleosome, which is represented by 100- to 200-bp DNA fragments.

### RNA stability assay

After transfection with related plasmids or small interfering RNA(SiRNAs), the cell medium was supplemented with Actinomycin D at a final concentration of 5 ug/ml. Following that, H1.0 mRNA was assessed using the RT-qPCR assay.

### RNA-seq analysis

The differentially expressed genes in Sg4 ATP2B4 and Ctrl Patu-8988T cells were identified using RNA-sequence (RNA-Seq) analysis by ANNOROAD GENE Technology Co., Ltd. (Beijing, China).

### Biotin pulldowns and mass spectrometry analysis

Using the TurboID method, proteins in close proximity to ATP2B4 were biotinylated and isolated by streptavidin-bead pulldowns. Briefly, 24 h after biotin supplementation (100 μM) in HEK-293T cells transfected by plv-HA-empty-TurboID or plv-HA-ATP2B4-TurboID plasmids, All the proteins were collected and treated by Biotinylated Protein Pull-Down Kit (Beyotime, P0658S). Biotinylated proteins were identified using Mass spectrometry analysis by Shanghai Applied Protein Technology Co., Ltd (Shanghai, China), relative data are shown in Table S2.

### RNA immunoprecipitation (RIP) assay

The cells were first collected and treated according to BeyoRIP™ RIP Assay Kit (Beyotime, P1801S). Antibodies against HuR and IgG were then added to the whole lysate. Protein A/G plus-agarose was employed to extract RNA-protein complexes and was rinsed four times with lysis buffer. Finally, the precipitated RNA was quantified using RT-qPCR.

### Xenograft tumor models

Male Balb/c nude mice (4-6 weeks) were obtained from the Changzhou Cavens Laboratory Animal Company. All the animal experiments were performed under the standard conditions in Animal Center of Jiangsu University and by the Committee on the Use of Live Animals for Teaching and Research of Jiangsu University (UJS-IACUC-AP-2025031803). No blinding was performed during animal experiments. Mice were randomly divided into six groups (*n* = 6), BXPC3 cell xenograft models were established by the subcutaneous injection of 1 × 10^7^ of the control and sgATP2B4 cells. When tumors reached a volume of approximately 50 mm^3^, about a week post-tumor inoculation, mice were treated with radiotherapy (6 Gy, twice). Tumor volume was calculated every five days as length × width^2^ × 1/2, After 7 weeks of observation, mice were sacrificed; Tumor tissues were removed and weighed.

### Immunohistochemistry (IHC) assay

Slices of mice xenograft were collected and embedded in paraffin after fixation in 4% paraformaldehyde. The 5 µm-thick tissue slices were incubated with primary antibodies targeting ATP2B4 overnight at a temperature of 4 °C. After staining, the sections were rinsed with PBS and then incubated with a secondary antibody at room temperature for 1 h. The staining was completed using diaminobenzidine as the chromogen.

### Statistical analysis

All results are expressed as the mean ± standard deviation (SD). All samples were independent biological replicates. Student *t* test, unpaired or paired, was used to compare differences between the two groups. One-way or two-way ANOVA test, followed by post hoc Tukey multiple comparisons test was used for the analysis among three or more groups. Statistical significance was judged by ∗*p* < 0.05, ∗∗*p* < 0.01, ∗∗∗*p* < 0.001.

## Supplementary information

Figure S1

Figure S2

Figure S3

Figure S4

Supplementary Figure Legends

Table S1

Table S2

original WB

## Data Availability

All relevant data generated in this study are available within the article and Supplementary files.
