# Supplementary figures and images for: ATP2B4 driven chromatin compaction exacerbates pancreatic cancer radiotherapy resistance
Source: Cell Death Discov. 2026 May 25;12:313. doi: 10.1038/s41420-026-03142-7 (PMC13381861; doi:10.1038/s41420-026-03142-7)

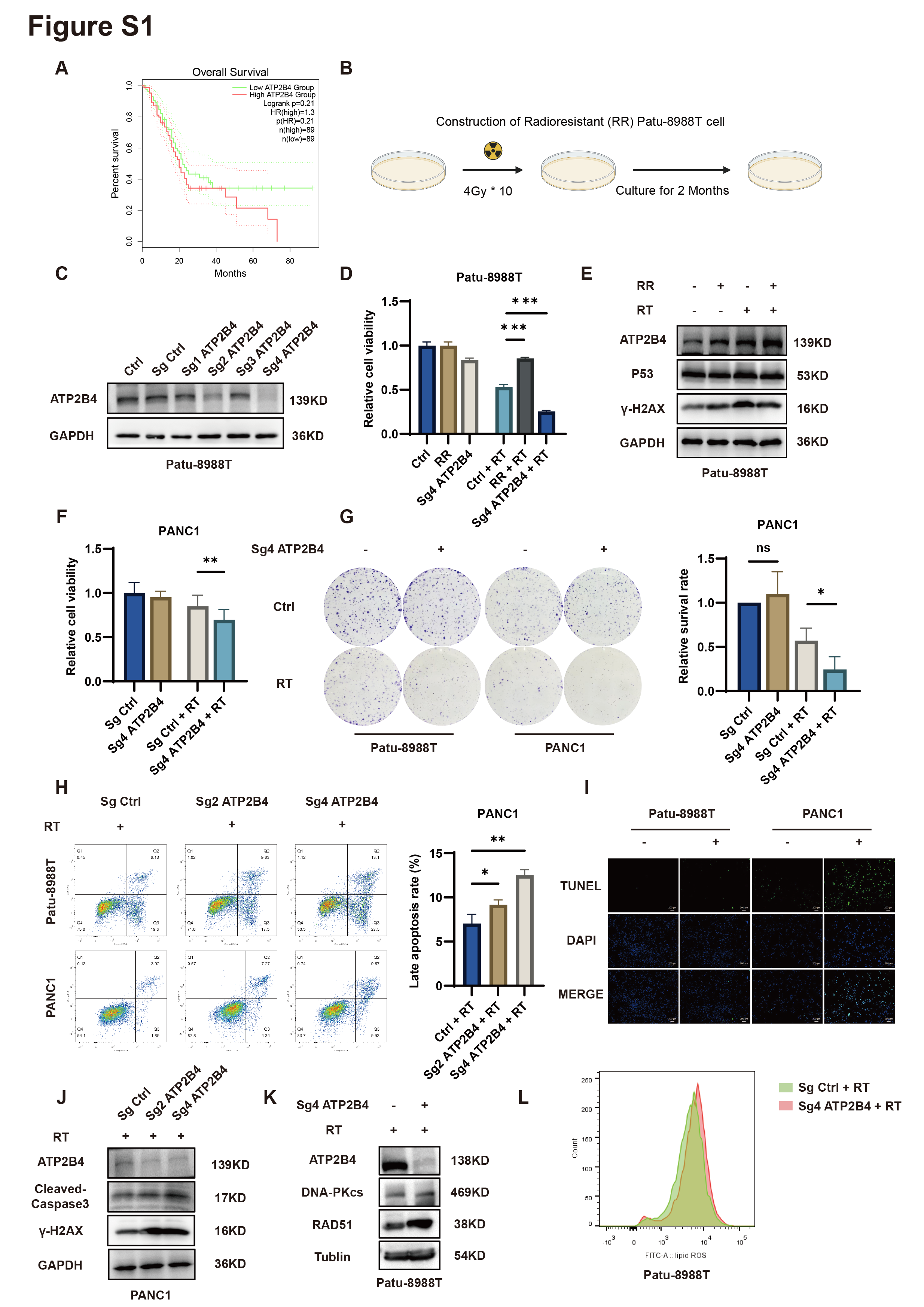

Supplement: Supplementary file 1 — Figure S1 [file 41420_2026_3142_MOESM1_ESM.png]

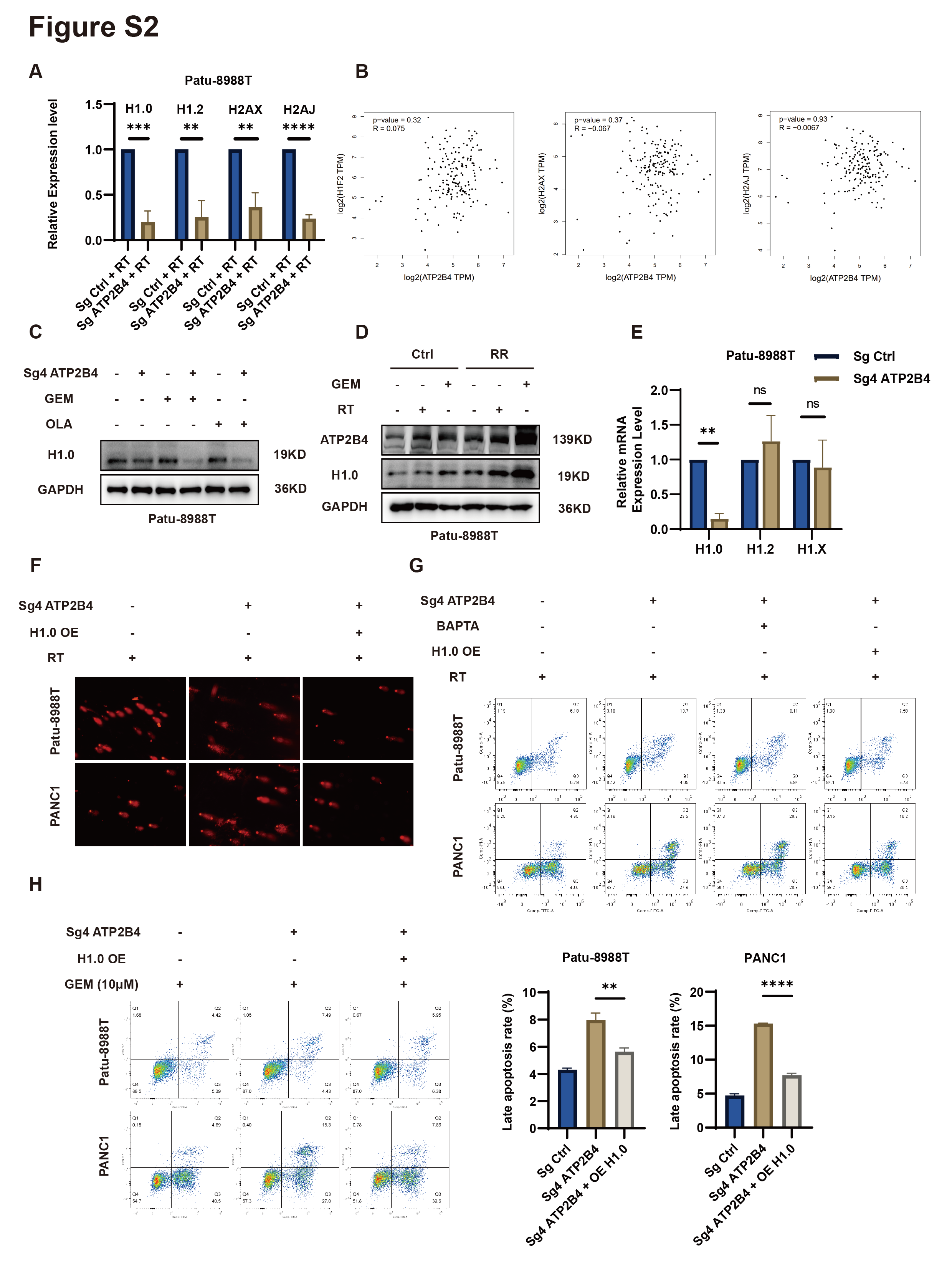

Supplement: Supplementary file 2 — Figure S2 [file 41420_2026_3142_MOESM2_ESM.png]

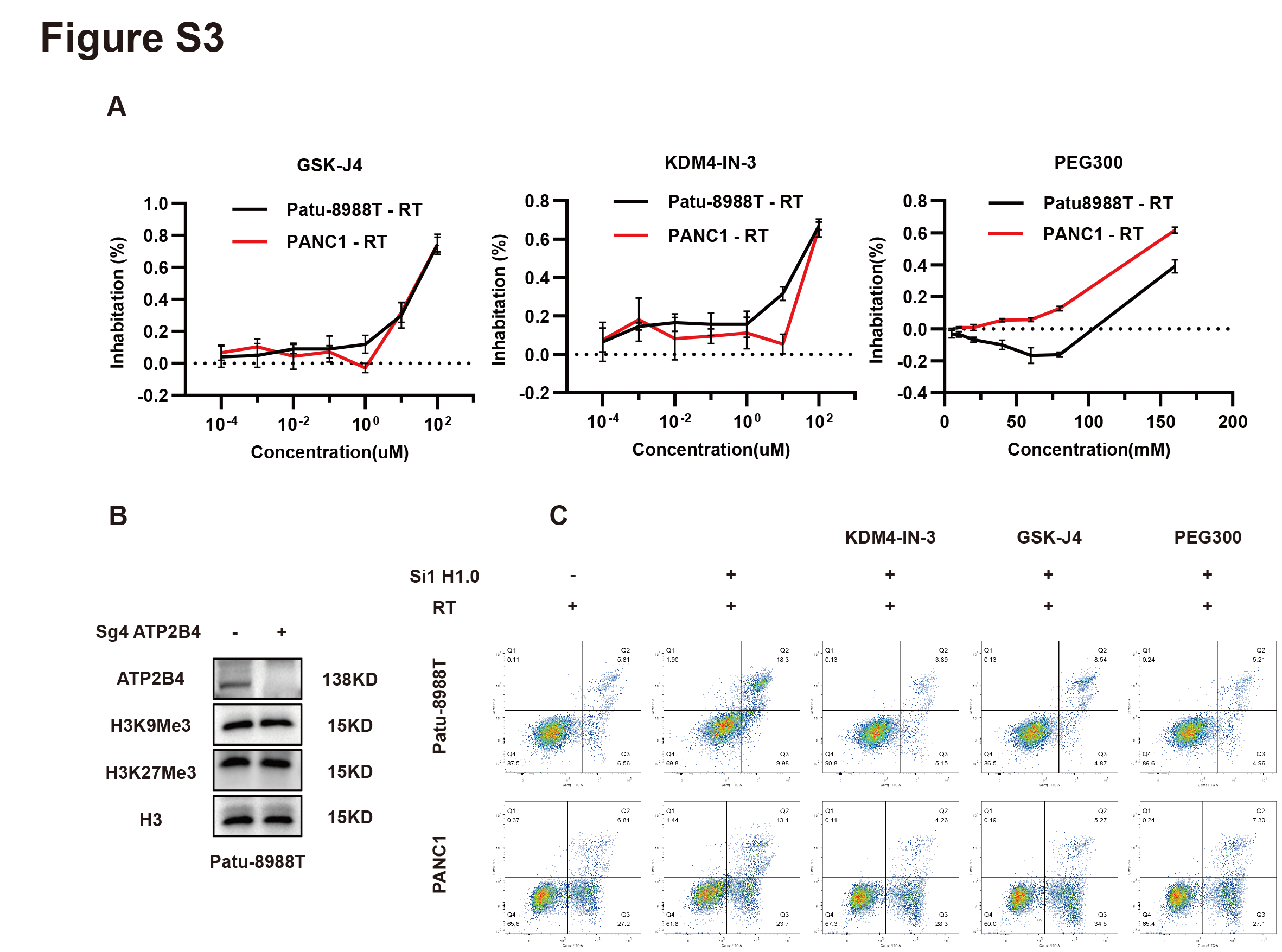

Supplement: Supplementary file 3 — Figure S3 [file 41420_2026_3142_MOESM3_ESM.png]

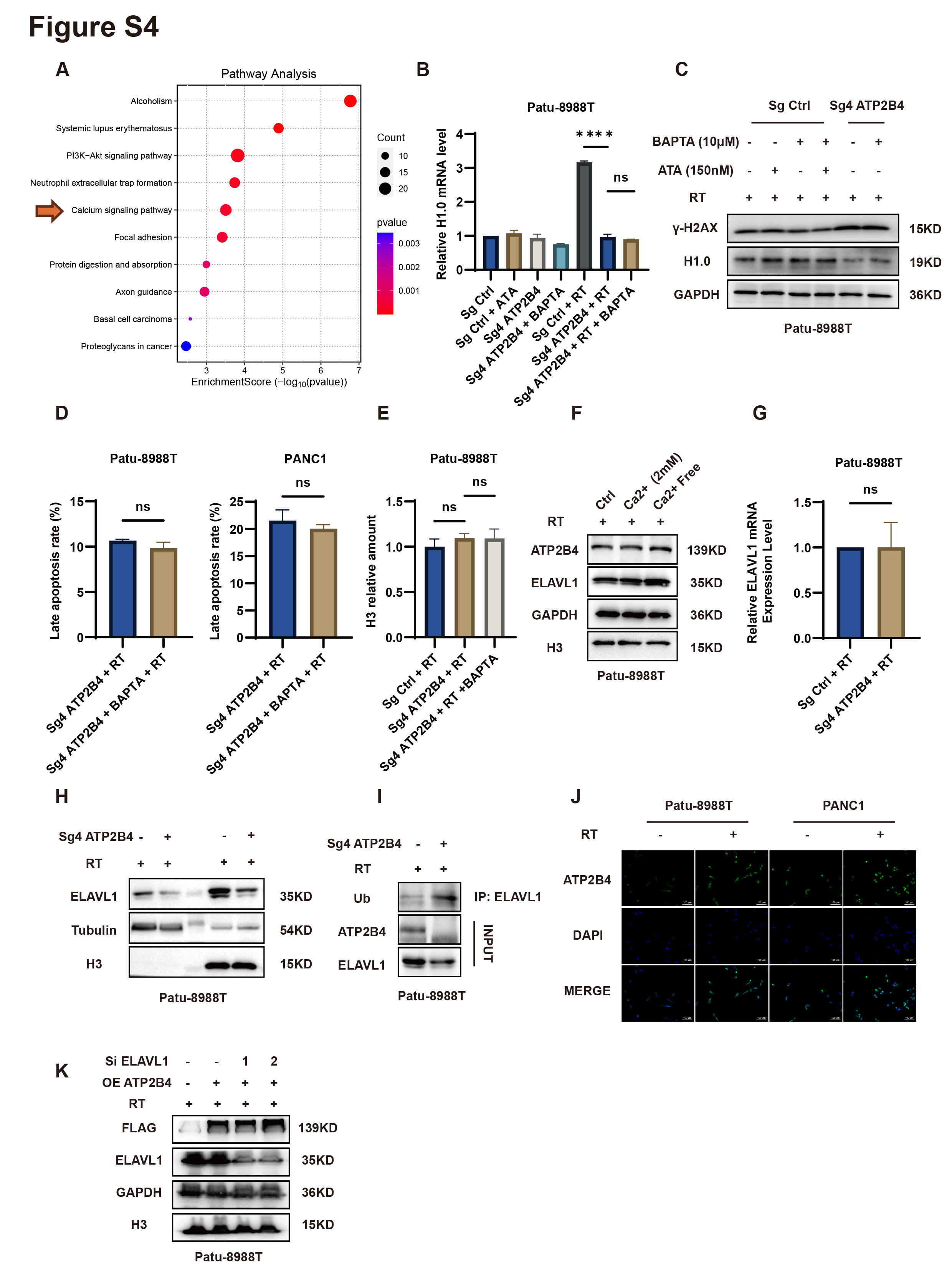

Supplement: Supplementary file 4 — Figure S4 [file 41420_2026_3142_MOESM4_ESM.png]
