# Supplementary material for: ATP2B4 driven chromatin compaction exacerbates pancreatic cancer radiotherapy resistance
Source: Cell Death Discov. 2026 May 25;12:313. doi: 10.1038/s41420-026-03142-7 (PMC13381861; doi:10.1038/s41420-026-03142-7)
Supplement: Supplementary file 5 — Supplementary Figure Legends [file 41420_2026_3142_MOESM5_ESM.docx]

**Fig S1. ATP2B4 mediating radioresistance of pancreatic cancer**

**A.** ATP2B4 expression level was not significantly associated with the overall survival rate of patients; data was obtained from TCGA database. **B.** The pattern diagram shows the RR cell line construction: Patu-8988T cells were cultured for more than 2 months after receiving a single dose of 4Gy radiation every other day with a total dose of 40gy. **C.** Validation of the efficiency of different ATP2B4 knockout plasmids in Patu-8988T cells through western blot. **D.** Cell viability of Patu-8988T Ctrl, ATP2B4 KO and RR cells were evaluated after 48 hours with or without RT (6 Gy), n = 3. **E.** Western blot of ATP2B4, P53 and γ-h2ax protein of Patu-8988T and RR cells 24h after treatment with or without RT (6 Gy). **F.** Cell viability of Panc-1 Ctrl and ATP2B4 KO cells were evaluated 24h after treatment with or without RT (6 Gy), n=9. **G-I.** Representative images of clonogenic survival assay, flow cytometry and tunel assay in Patu-8988T and Panc-1 cells. Clonogenic survival analysis and late apoptosis rate of Panc-1 cells was plotted, n=3. **J.** Western blot of ATP2B4, γ-h2ax and cleaved-caspase3 protein of Panc-1 Ctrl and ATP2B4 KO cells treated with RT (6 Gy). **K.** Western blot of ATP2B4, DNA-PKcs and RAD51 protein of Patu-8988T Ctrl and ATP2B4 KO cells treated with RT (6 Gy). **L.** ROS level of Patu-8988T Ctrl and ATP2B4 KO cells treated with RT (6 Gy). Data are presented as mean ± SD. **p* < 0.05, ***p* < 0.01,

**FigS2. H1.0 regulated by ATP2B4 plays an important role in the radioresistance of tumor cells**

**A.** qPCR analysis of H1.0, H1.2, H2AX and H2AJ mRNA expression in Patu-8988T cells, which were cultured in 10% fetal bovine serum for 24h after treatment with RT (6 Gy). **B.** The correlation between ATP2B4 and H1.2, H2AX, and H2AJ mRNA expression, data was obtained from TCGA database. **C.** Western blot of H1.0 protein of Patu-8988T Ctrl and ATP2B4 KO cells after treated with or without GEM (10μM) or OLA (5μM) for 24h. **D.** Western blot of H1.0 and ATP2B4 protein of Patu-8988T and RR cells treated with RT (6 Gy) or GEM (10μM). **E.** qPCR analysis of H1.0, H1.2 and H1.X mRNA expression in Patu-8988T cells. **F-G.** Representative images of comet assay, flow cytometry in Patu-8988T and Panc-1 cells. **H.** Representative images of flow cytometry of Patu-8988T and Panc-1 cells after treatment with GEM (10μM) for 24h, proportion of late apoptosis cells was plotted, n=3. Data are presented as mean ± SD. **p* < 0.05, ***p* < 0.01, ****p* < 0.001, *****p* < 0.0001.

**FigS3. Increased radiation-induced apoptosis due to H1.0 depletion could be reversed by drugs**

**A.** Cell viability of Patu-8988T and Panc-1 cells treated with gradient concentration of GSK-J4, KDM-IN-3, and PEG300. Maximum concentration of drug was determined with not significantly affect cell activity. GSK-J4 (1μM), KDM-IN-3 (1μM), PEG300 (80mM). **B.** Western blot of ATP2B4, H3K9Me3 and H3K27Me3 protein of Patu-8988T Ctrl and ATP2B4 KO cells. **C.** Representative images of flow cytometry in Patu-8988T and Panc-1 cells treated with chromatin compaction inducers.

**FigS4. Regulation of H1.0 mRNA levels by ATP2B4 is independent of its function as a calcium pump**

**A.** KEGG pathway enrichment analysis of downregulated genes from ATP2B4 KO Patu-8988T cells compared with Ctrl cells, both were treated with RT (6 Gy). Calcium related pathway was enriched. **B.** qPCR analysis of H1.0 mRNA expression of Ctrl and ATP2B4 KO Patu-8988T cells treated with ATA (10μM) or BAPTA (150nM) for 24h before RT (6Gy). **C.** Western blot of H1.0 and γ-h2ax protein of Ctrl and ATP2B4 KO Patu-8988T cells treated with ATA (10μM) or BAPTA (150nM) for 24h before RT (6Gy). **D.** Flow cytometry was used to measure apoptosis progression of Patu-8988T and Panc-1 ATP2B4 KO cells treated with BAPTA (150nM) or not, proportion of late apoptosis cells was plotted, n=3. **E.** H3 protein levels of Ctrl and ATP2B4 KO cells treated with BAPTA (150nM) or not in Patu-8988T cells were assessed by quantitative Western blotting. The intensity of Coomassie staining was used as loading control and normalization, n=3. **F.** Western blot of ELAVL1 and ATP2B4 protein levels in Patu-8988T cells treated with additional Ca^2+^ (2mM) or Ca^2+^ free medium, n=3. **G.** mRNA level of ELAVL1 in Ctrl and ATP2B4 KO Patu-8988T cells treated with RT (6Gy), n=3. H. Western blot of ELAVL1, Tubulin and H3 protein levels in Patu-8988T Ctrl and ATP2B4 KO cells. **I.** Western blot of ELAVL1 and ATP2B4 protein levels in Patu-8988T Ctrl and ATP2B4 KO cells, ELAVL1 ubiquitination was detected by immunoprecipitation using an anti-ELAVL1 antibody followed by immunoblotting with an anti-ubiquitin antibody. **J.** Representative images of fluorescence of ATP2B4 in Patu-8988T and Panc-1 cells treated with RT (6Gy). **K.** Immunoprecipitation was performed to verify the efficacy of ATP2B4 overexpression and ELAVL1 knockdown in HEK-293T cells. Data are presented as mean ± SD. *****p* < 0.0001.
