# Supplementary material for: ATP2B4 driven chromatin compaction exacerbates pancreatic cancer radiotherapy resistance
Source: Cell Death Discov. 2026 May 25;12:313. doi: 10.1038/s41420-026-03142-7 (PMC13381861; doi:10.1038/s41420-026-03142-7)

F1D.

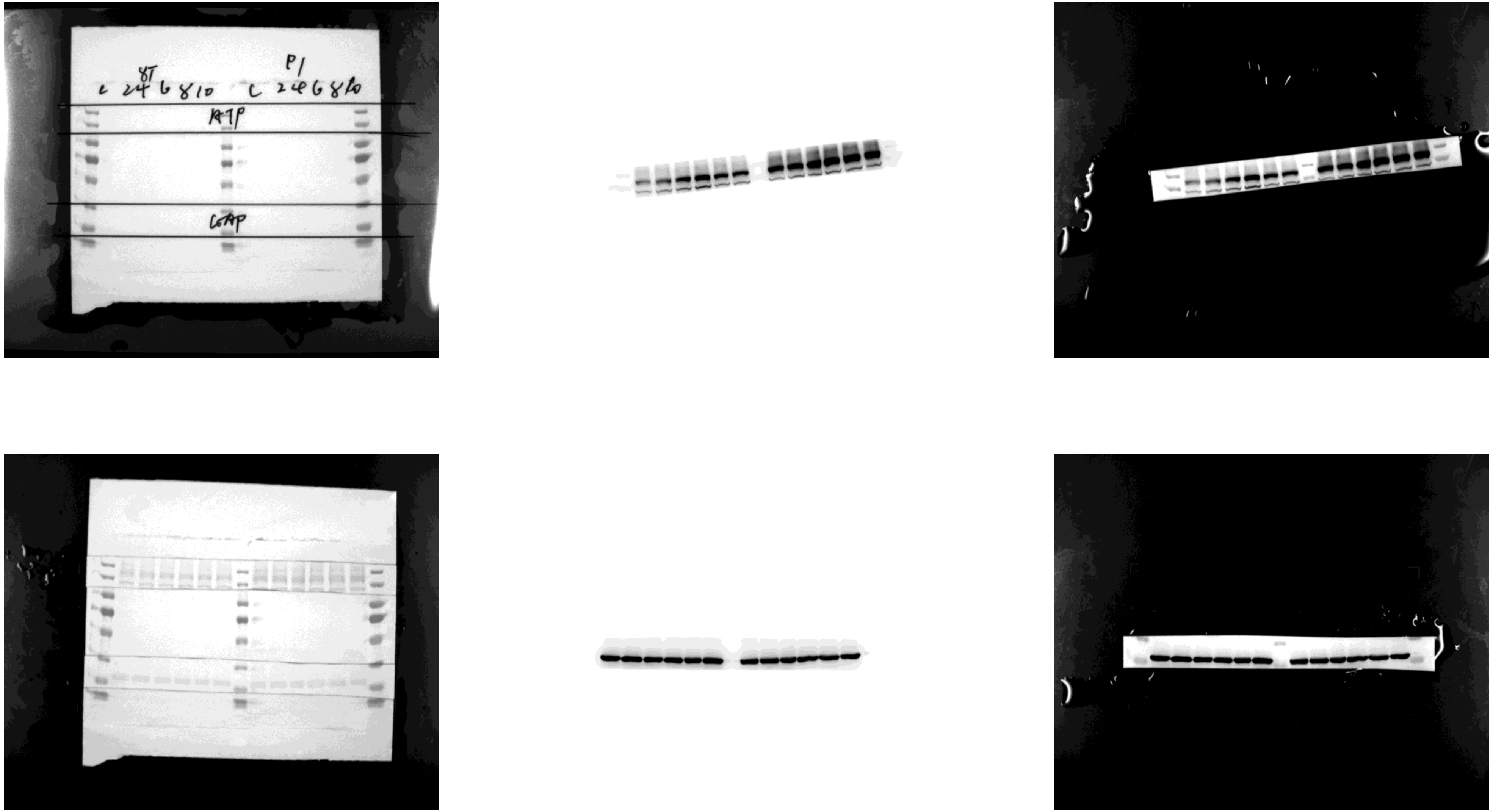

ATP2B4

GAPDH

F1D.

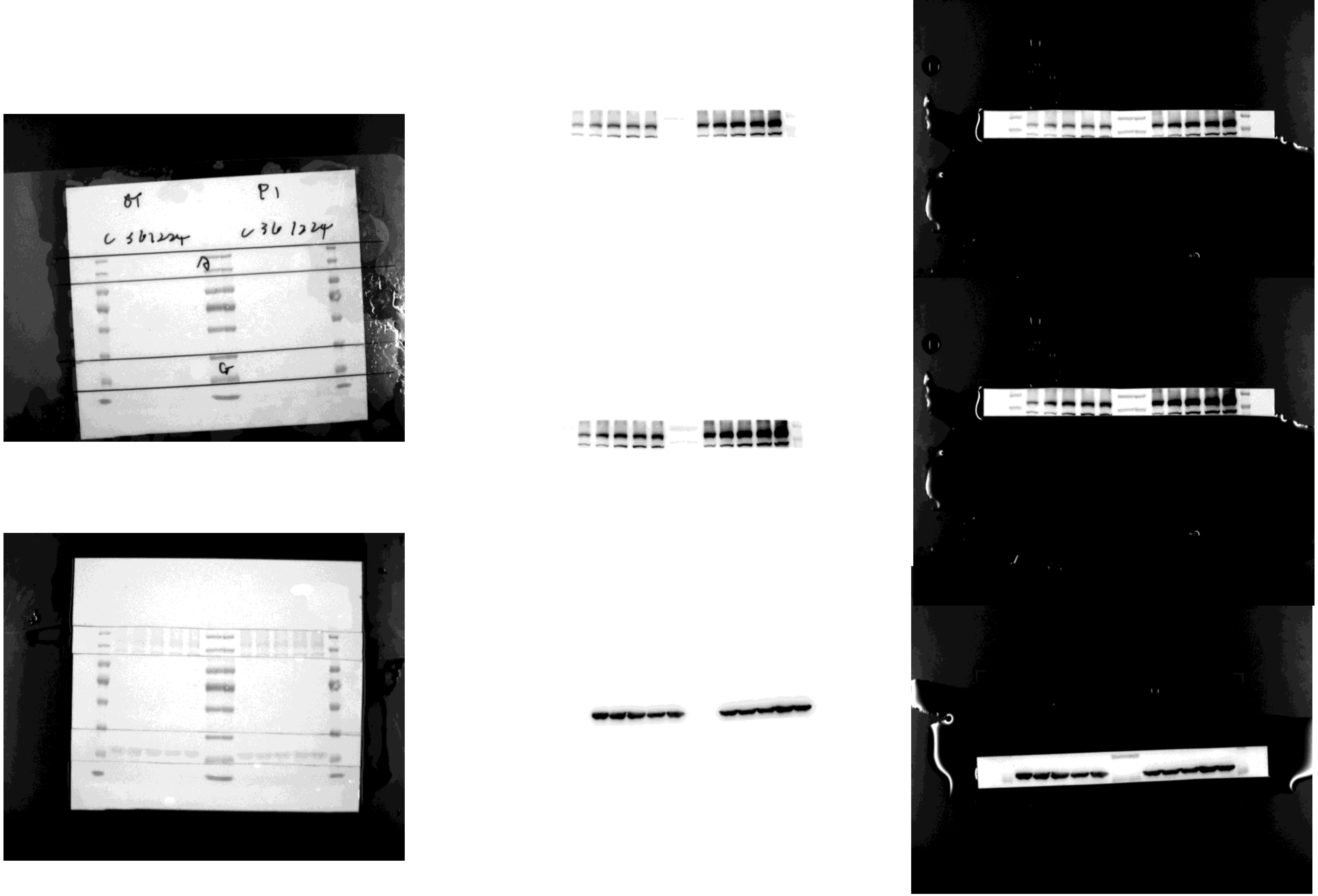

ATP2B4-Short exposure time

ATP2B4-Long exposure time

GAPDH

F1J.

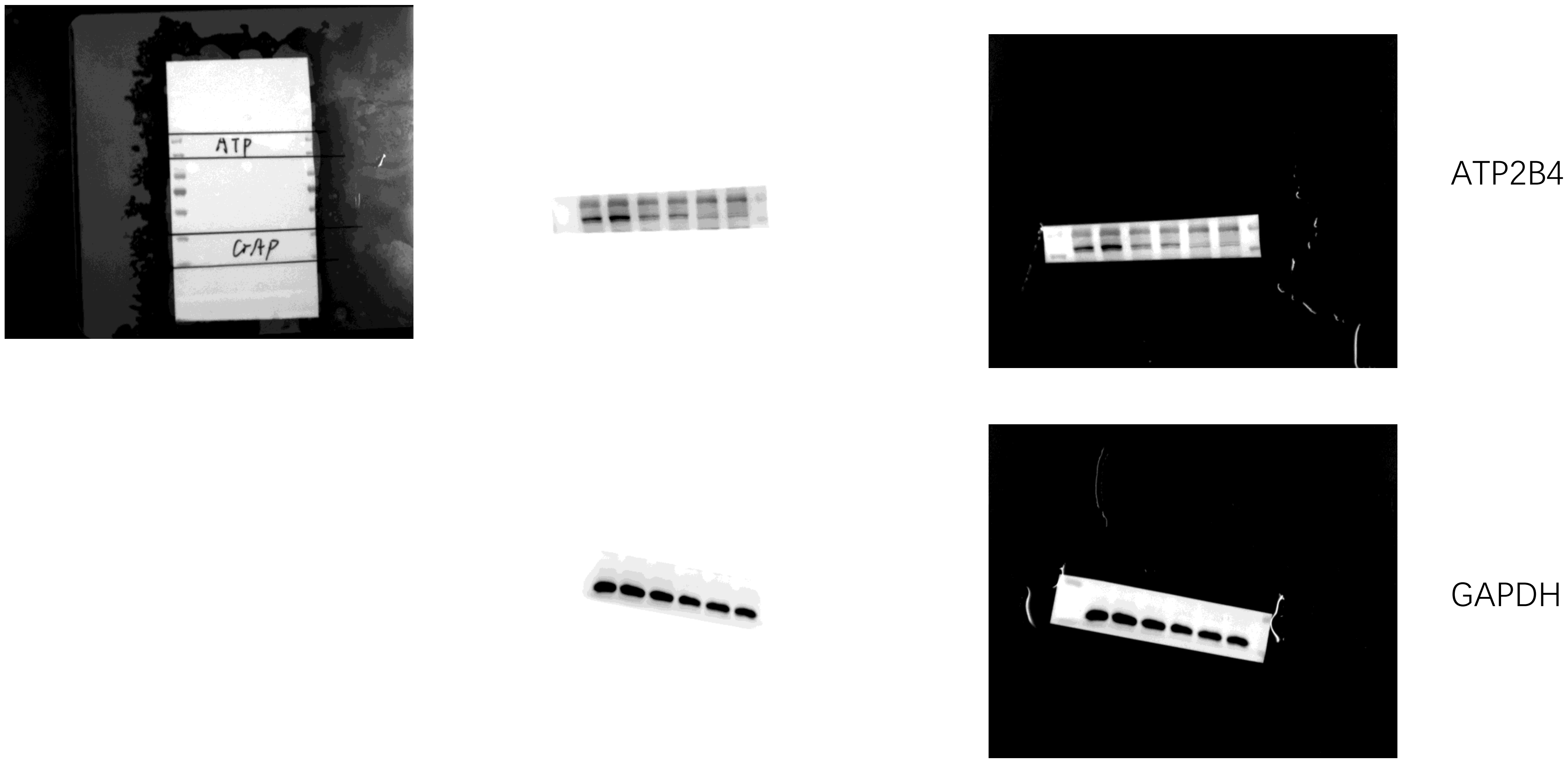

F1J.

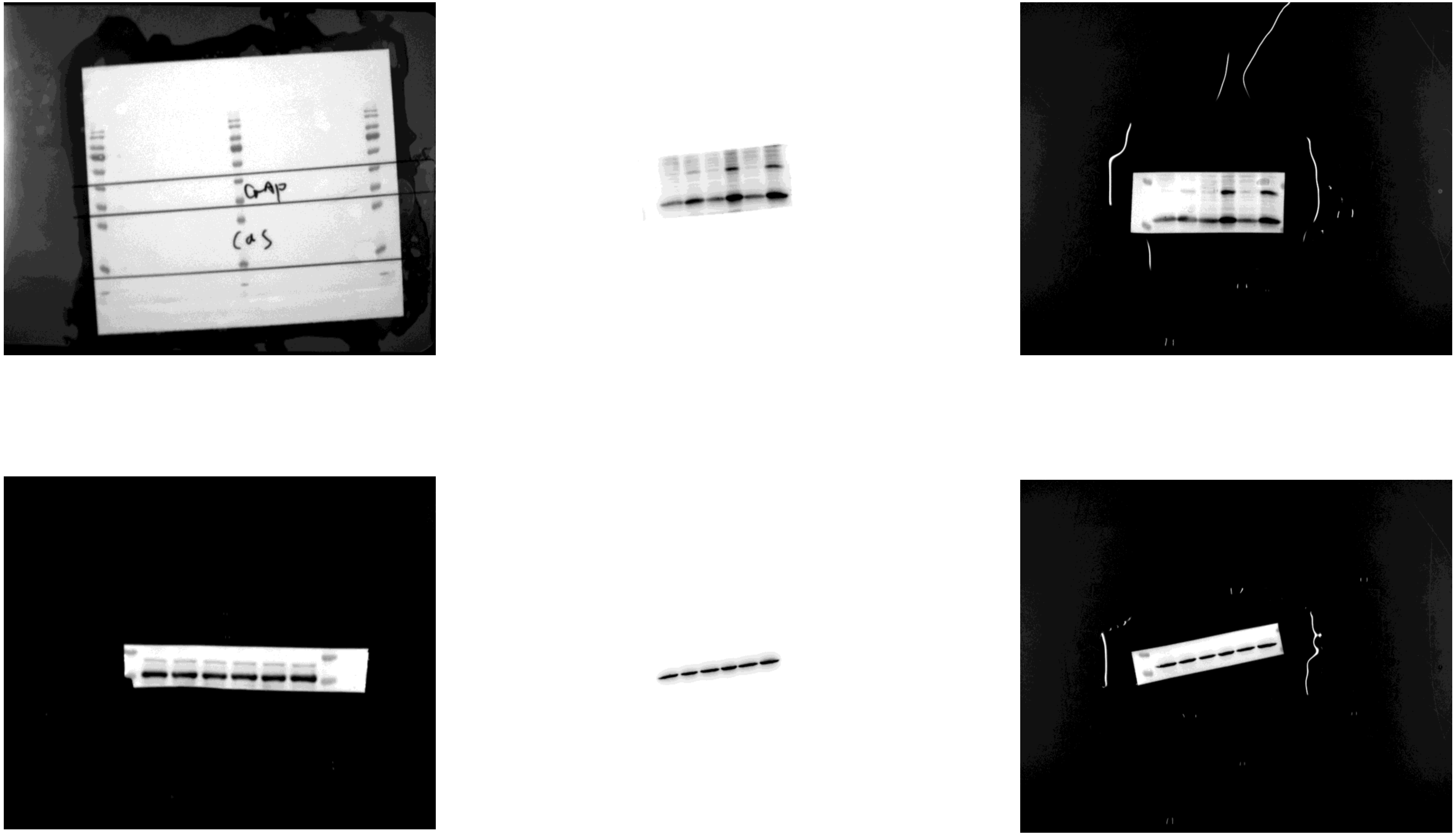

Cleaved-Caspase3

GAPDH

Caspase3

F1J.

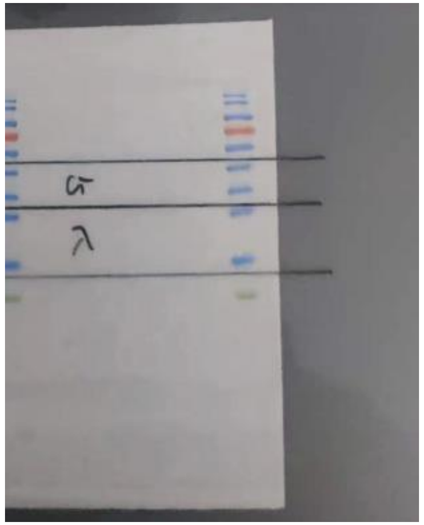

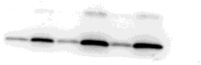

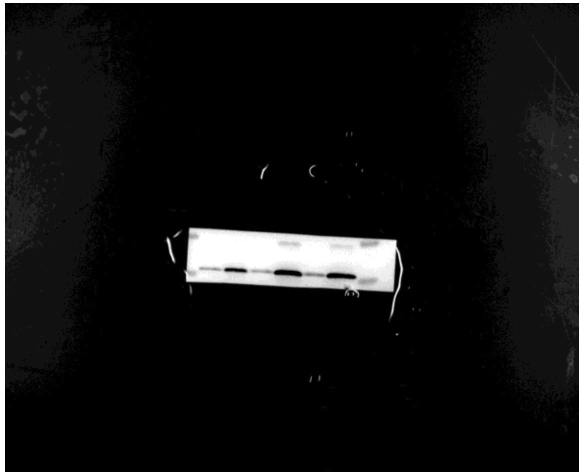

gamaH2AX

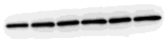

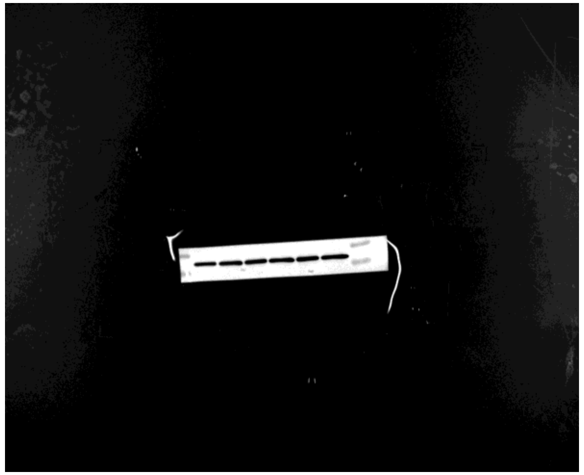

GAPDH

F1K.

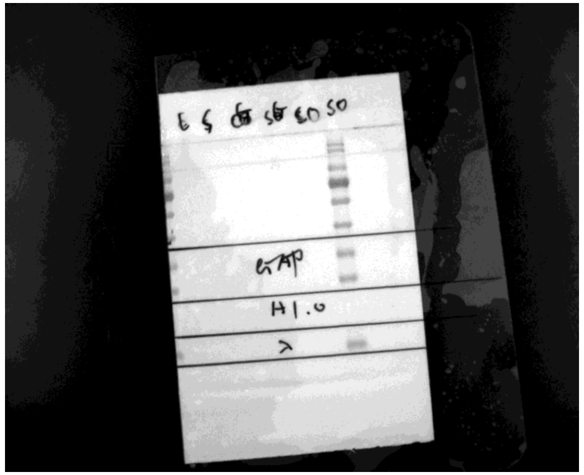

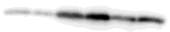

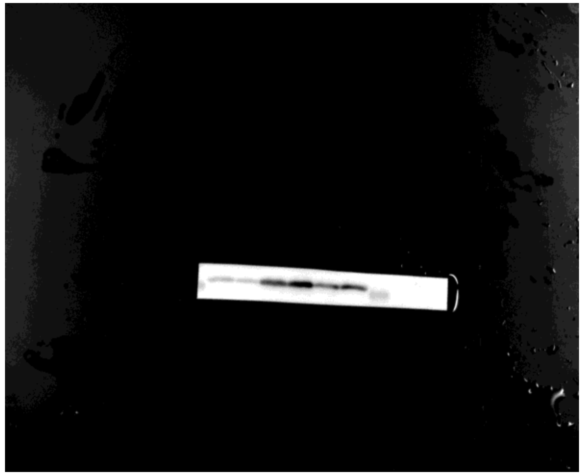

gamaH2AX

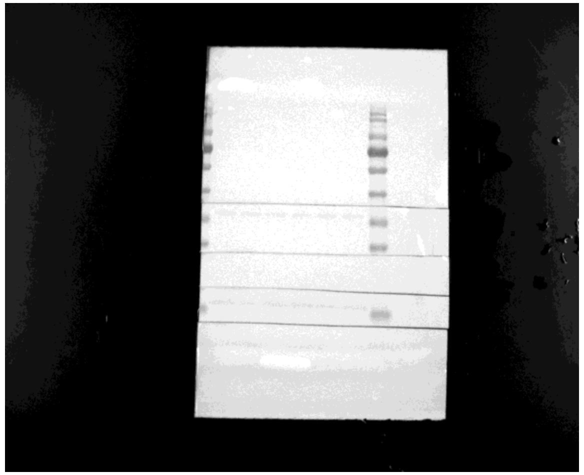

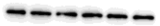

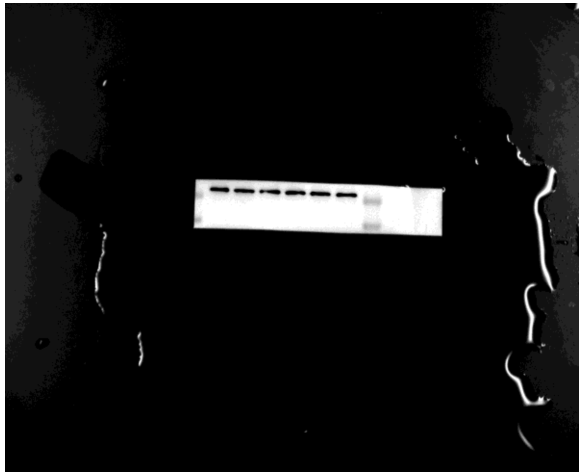

GAPDH

F1K.

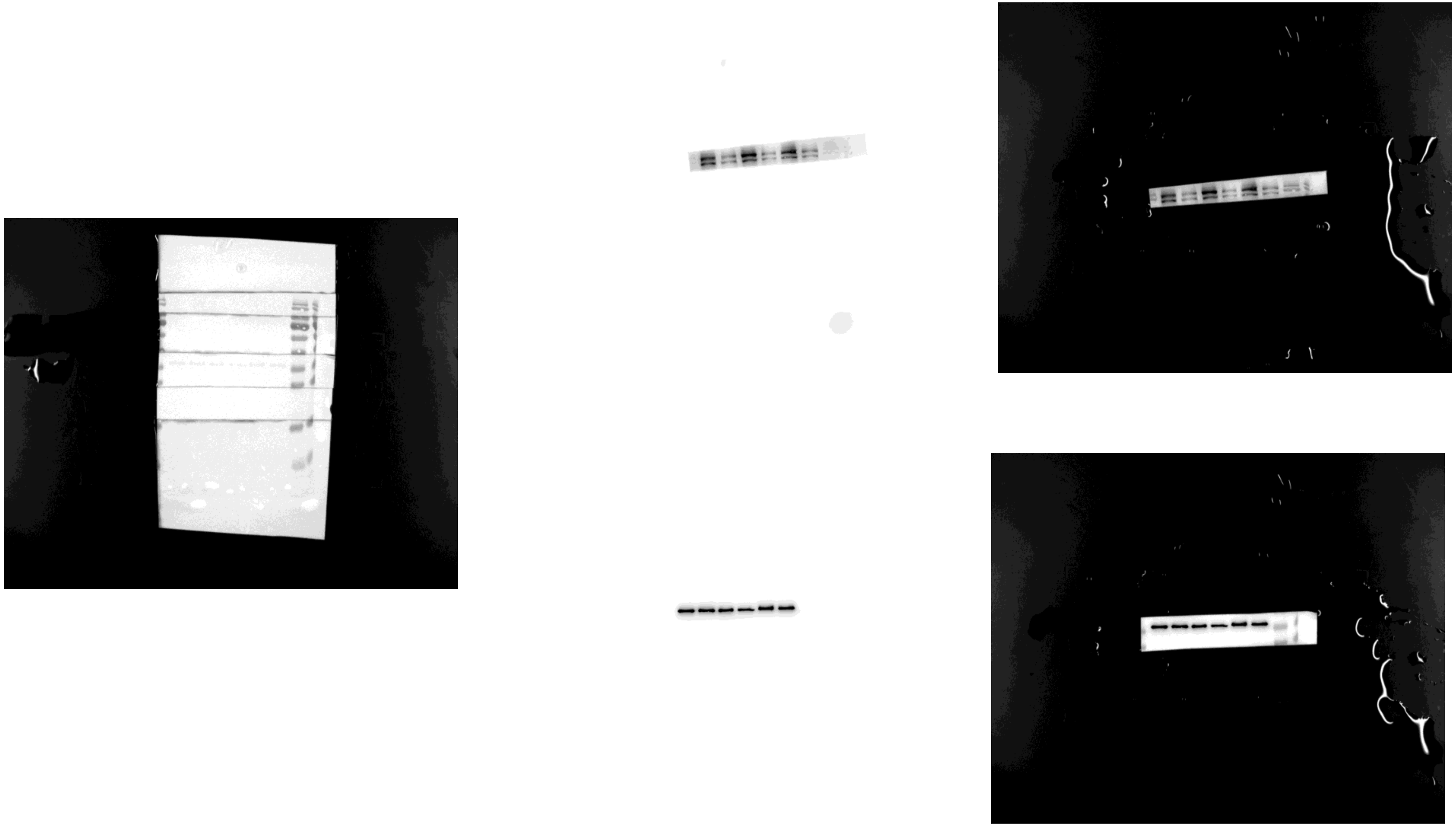

ATP2B4

GAPDH

F2D

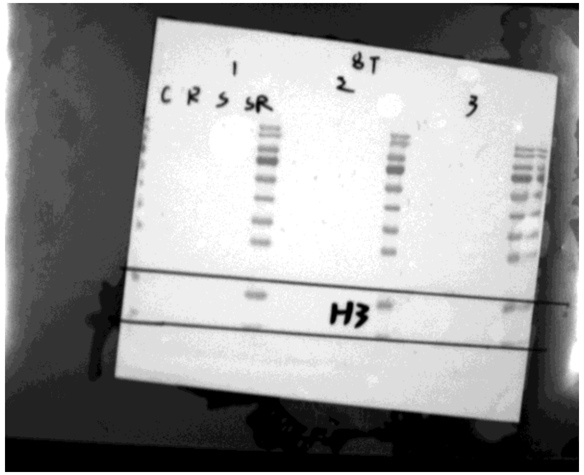

H3

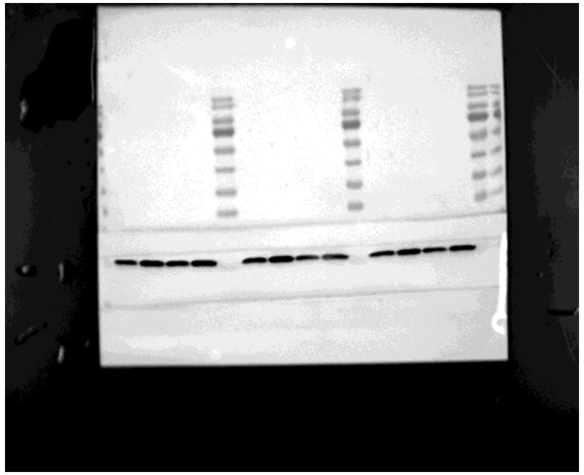

coomassie blue staining

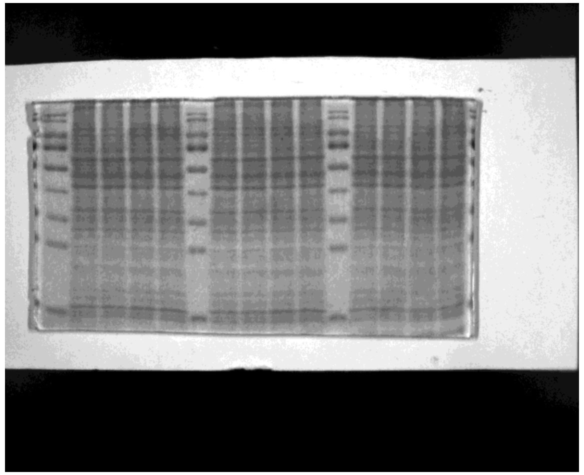

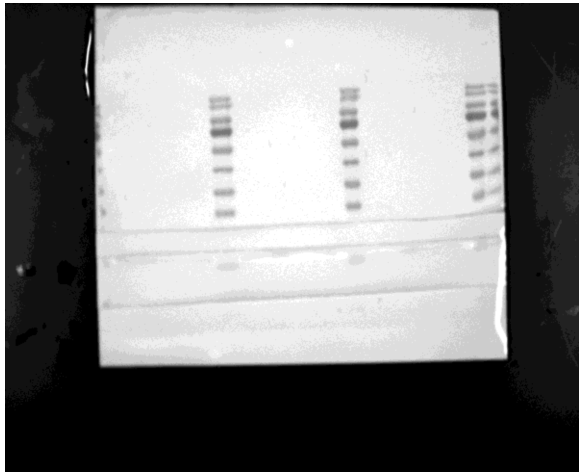

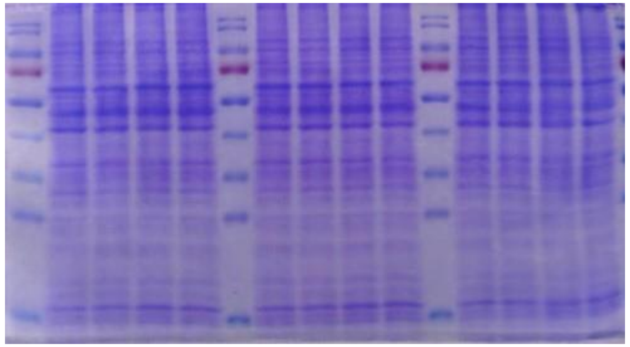

F2H.

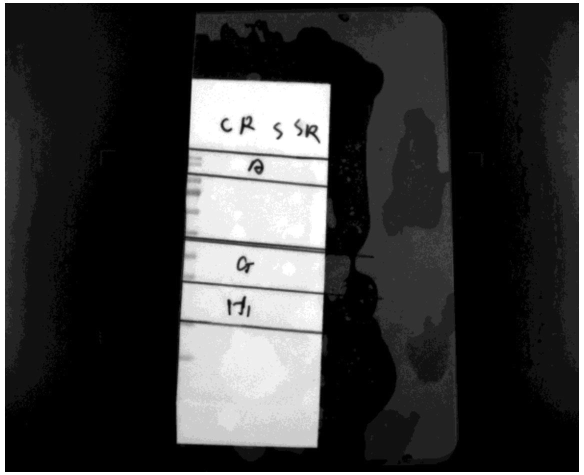

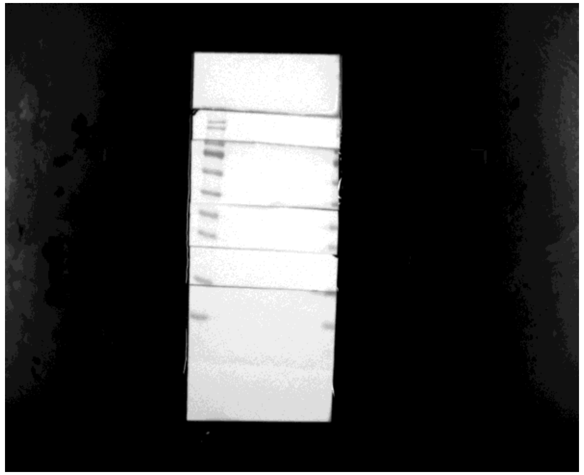

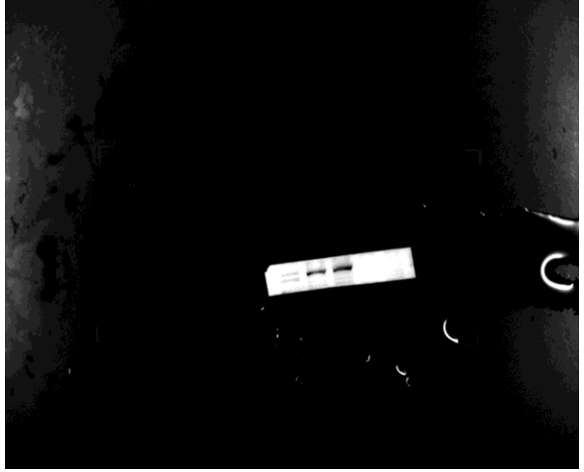

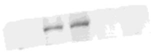

ATP2B4

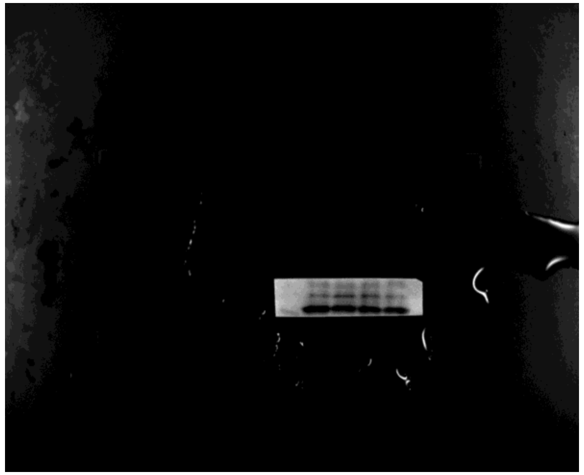

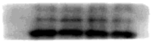

H1.0

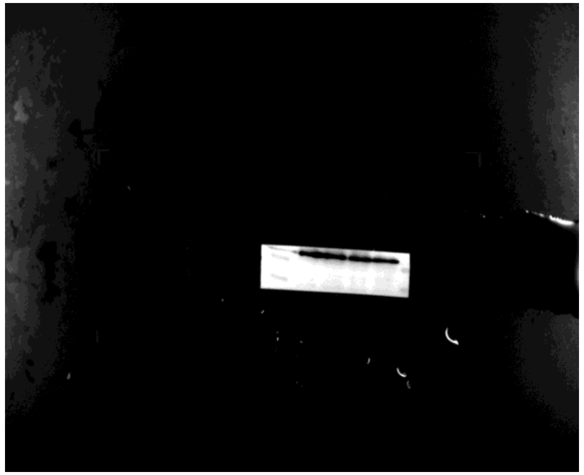

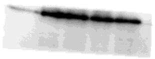

GAPDH

F2I.

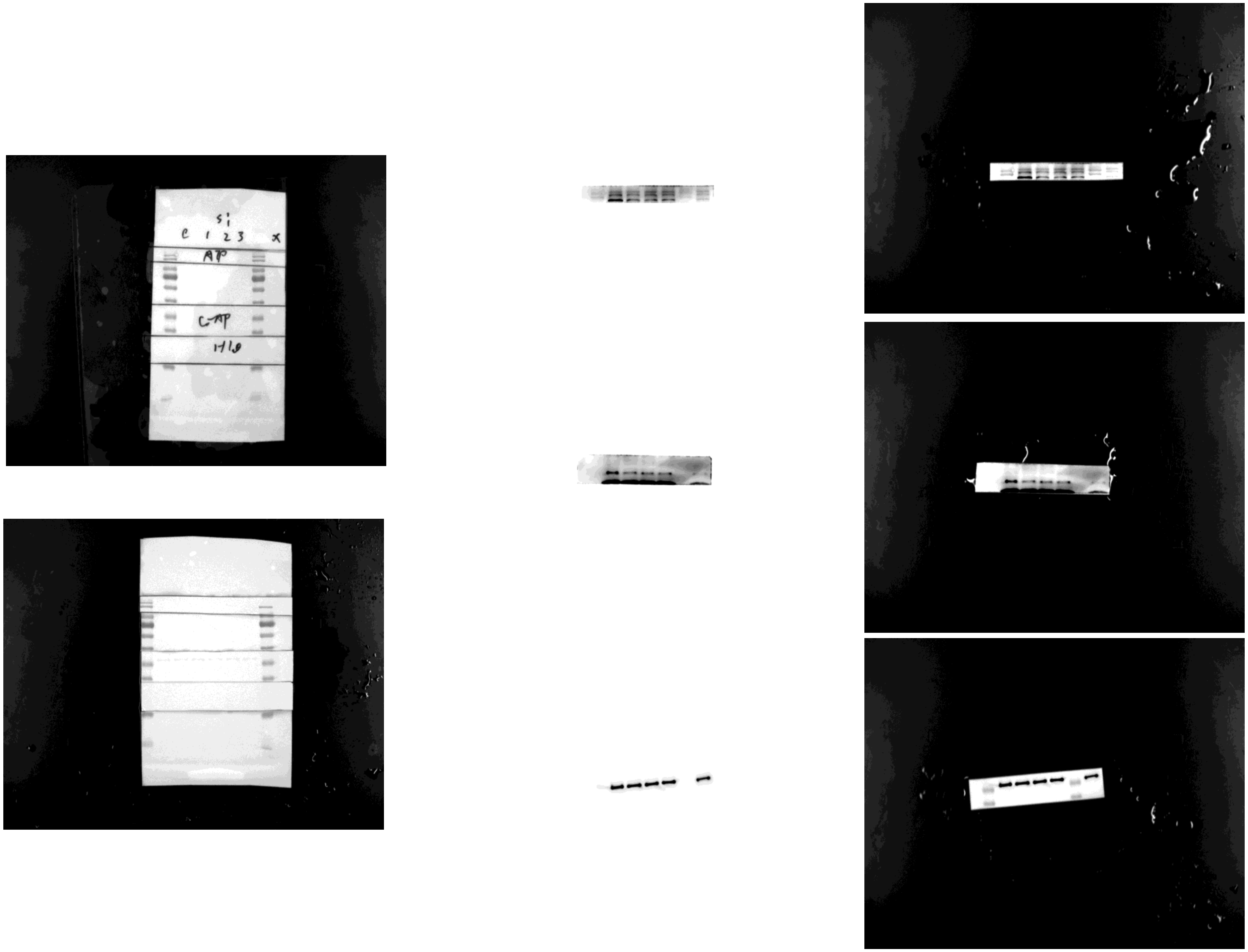

ATP2B4

H1.0

GAPDH

F2I.

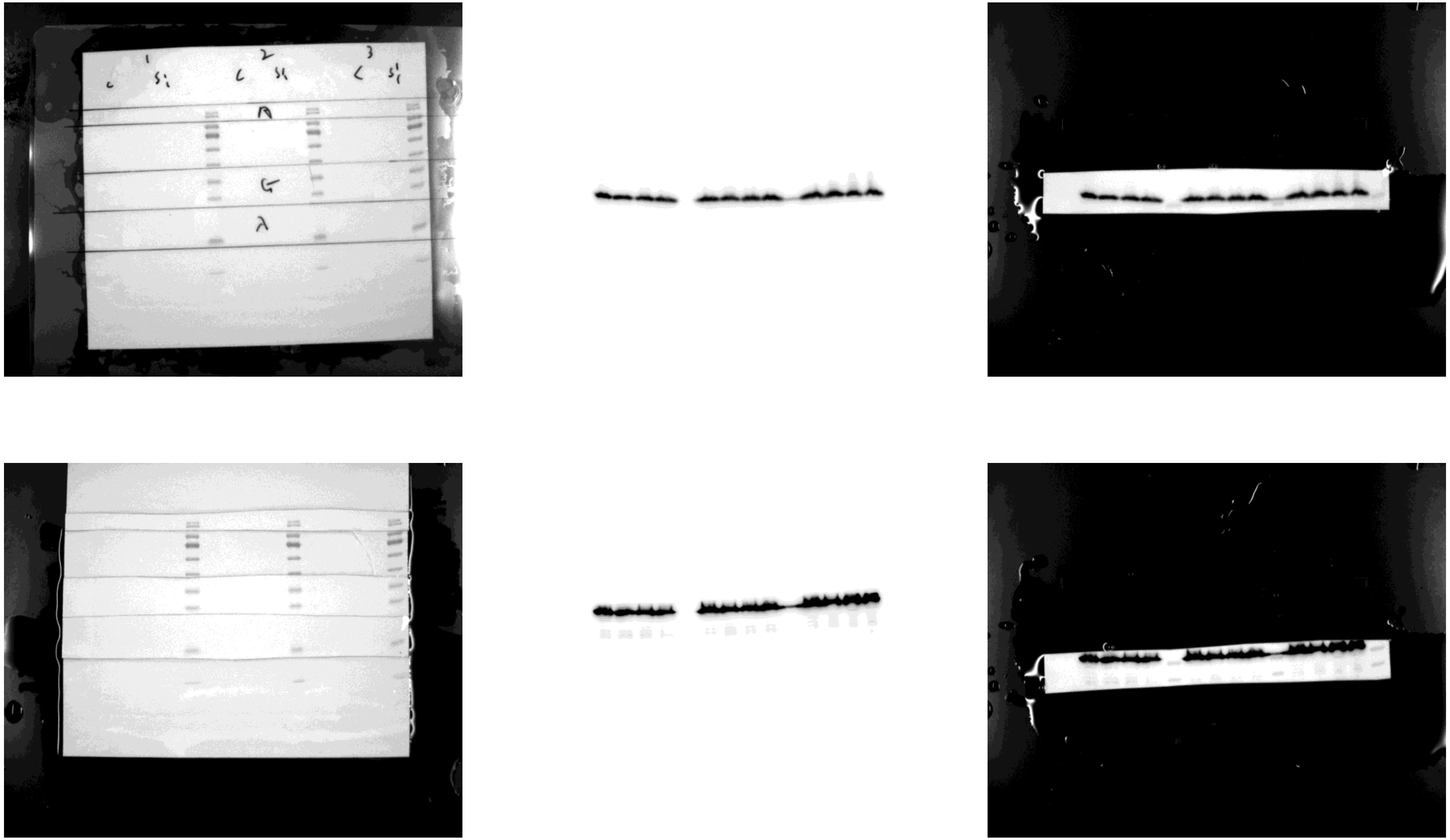

gamaH2AX

GAPDH

F2J-Panc1

GAPDH

H1.0

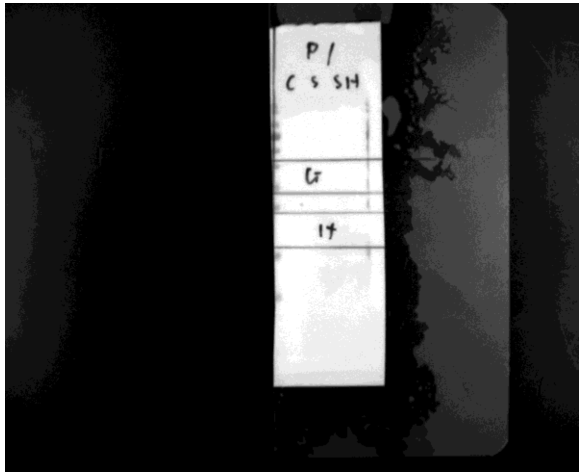

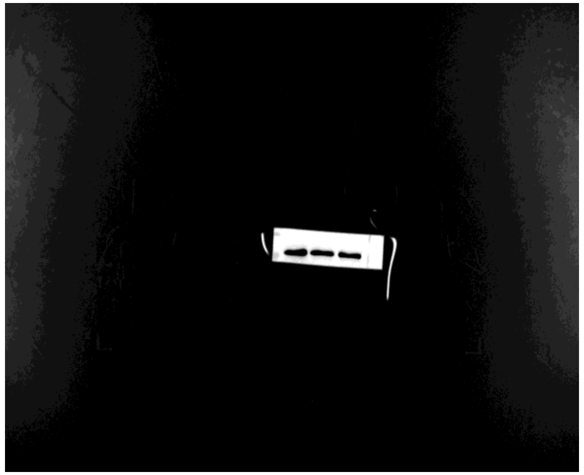

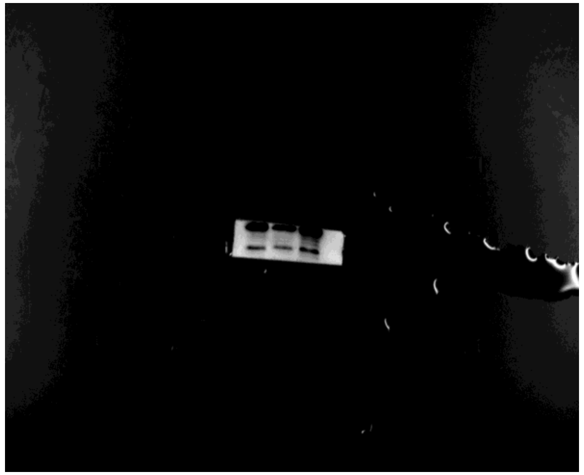

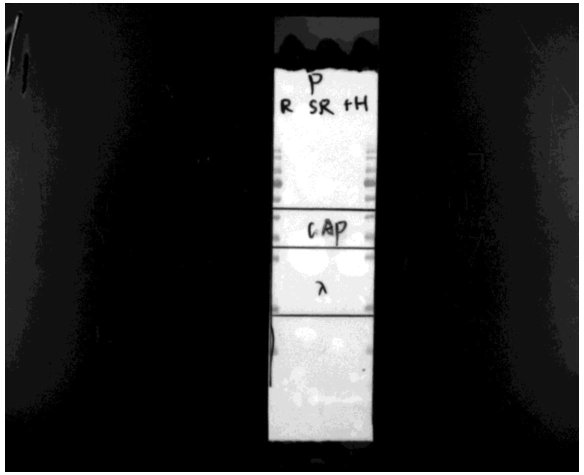

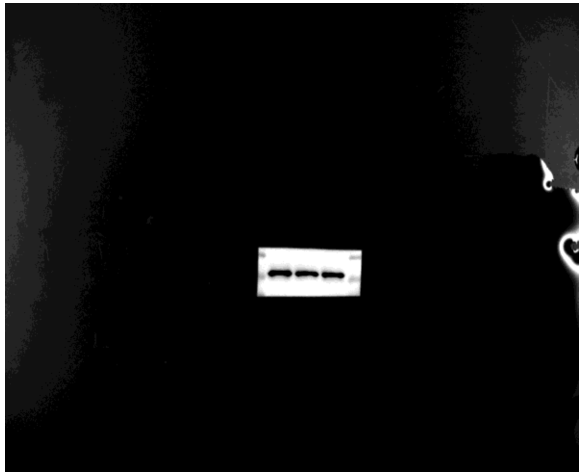

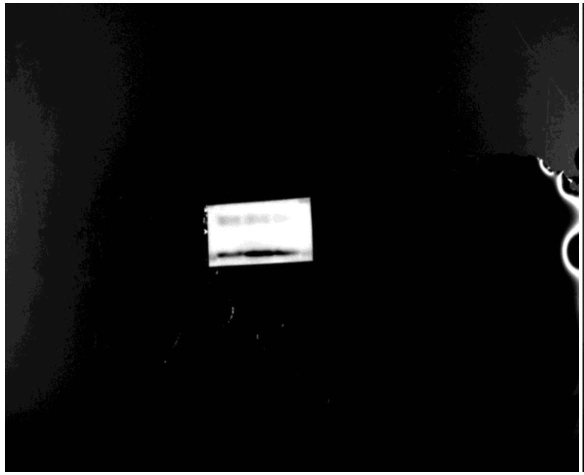

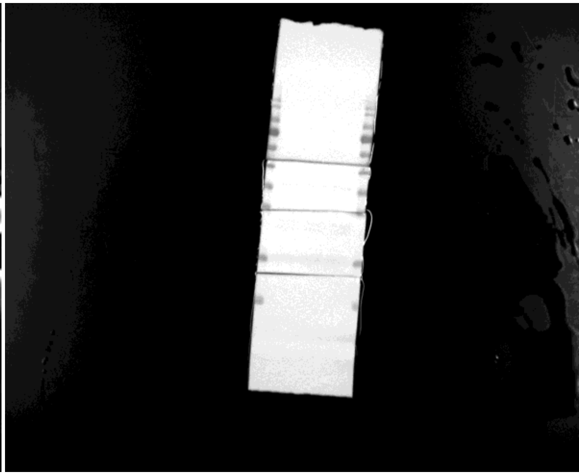

GAPDH

gamaH2AX

F2J-Panc1

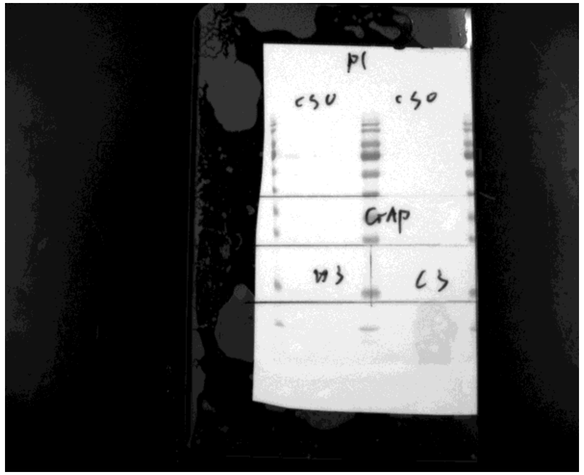

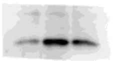

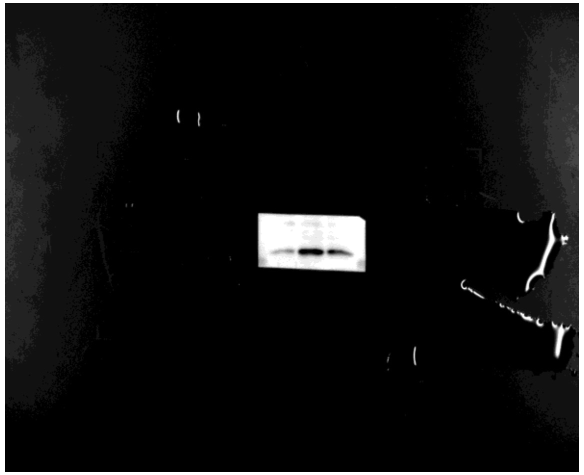

Cleaved-Caspase3

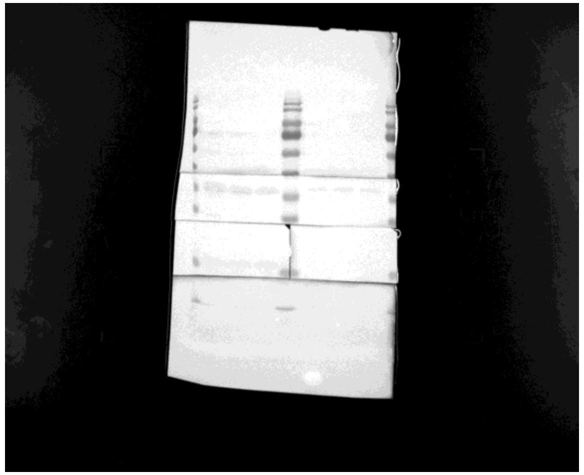

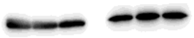

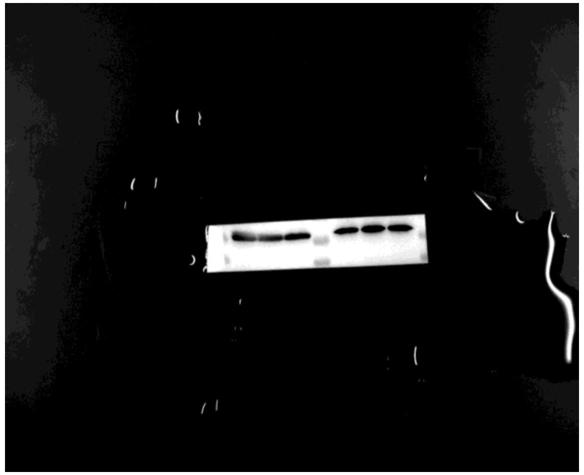

GAPDH

F2J-patu-8988T

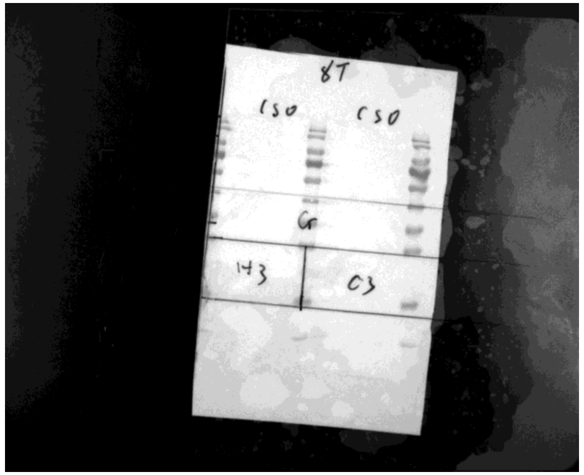

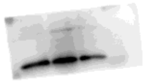

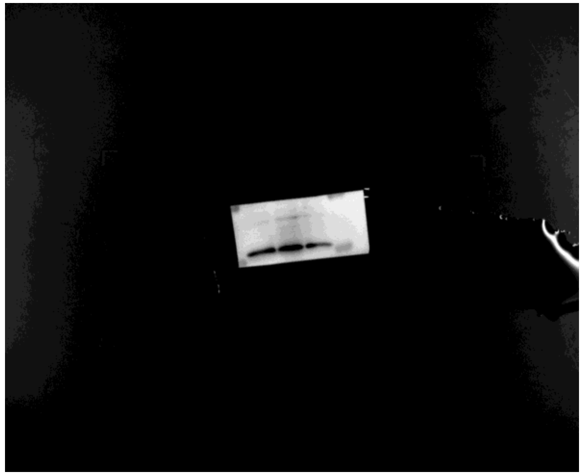

Cleaved-Caspase3

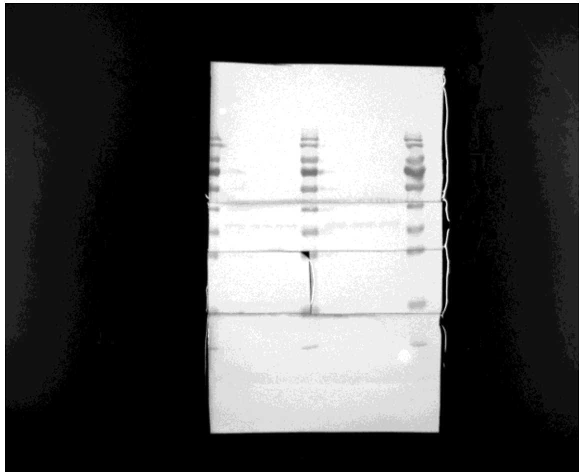

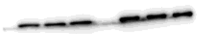

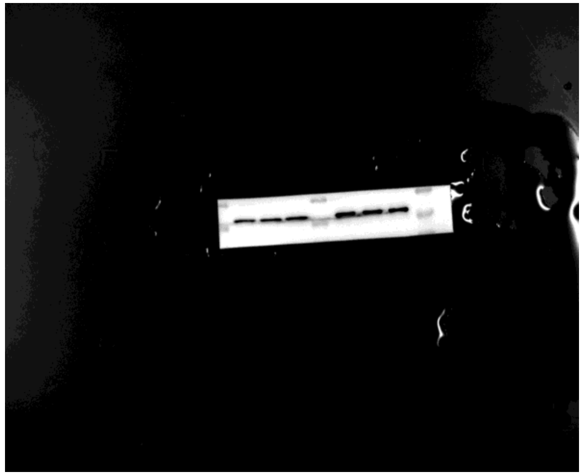

GAPDH

F2J-patu-8988T

GAPDH

H1.0

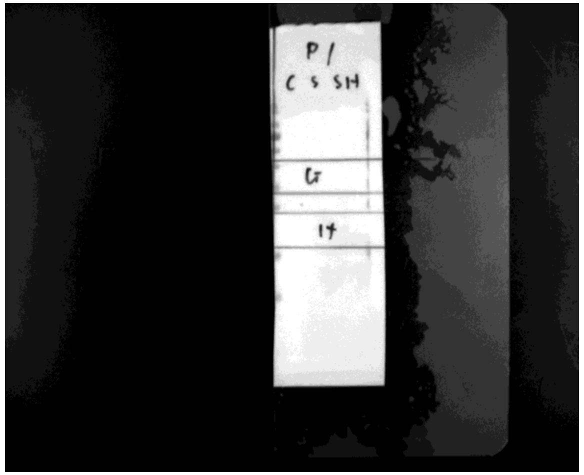

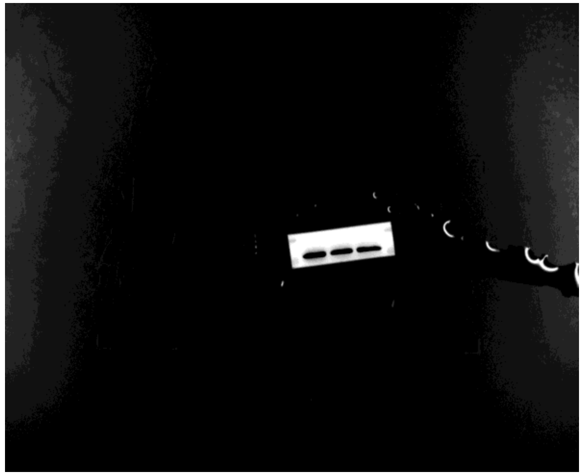

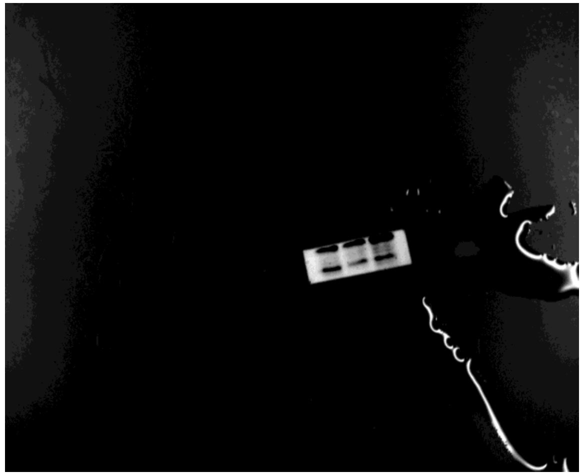

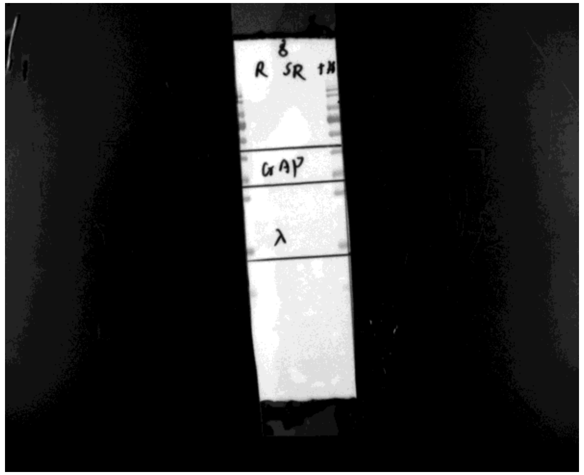

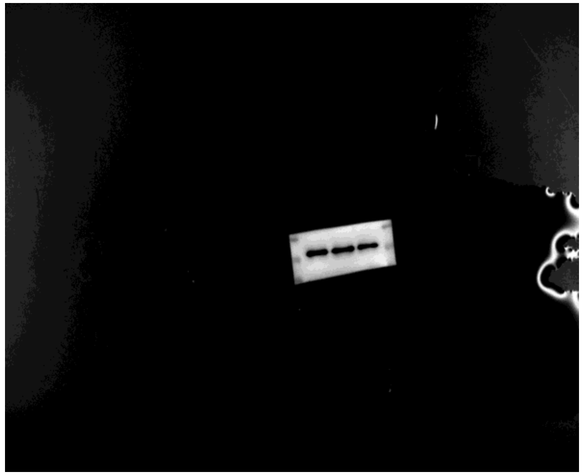

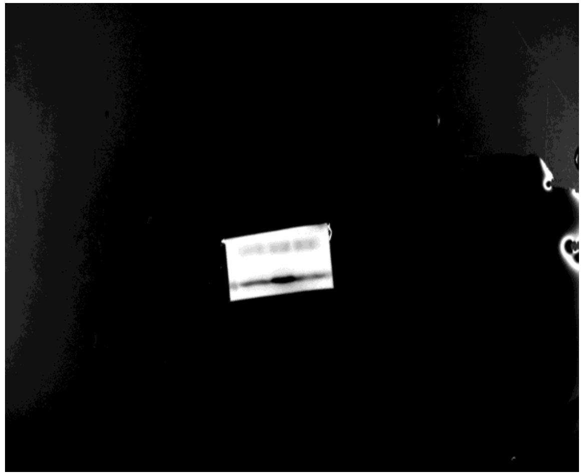

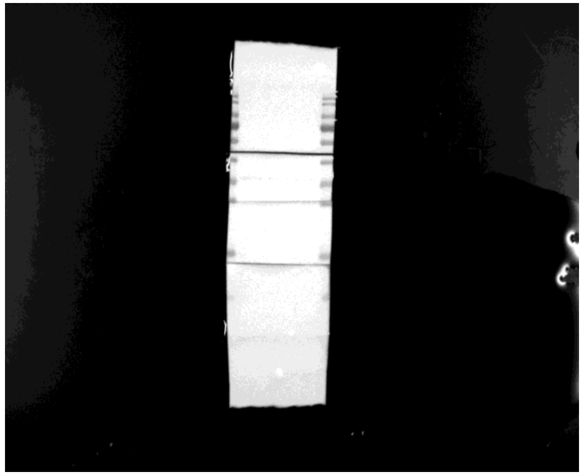

GAPDH

gamaH2AX

F3A

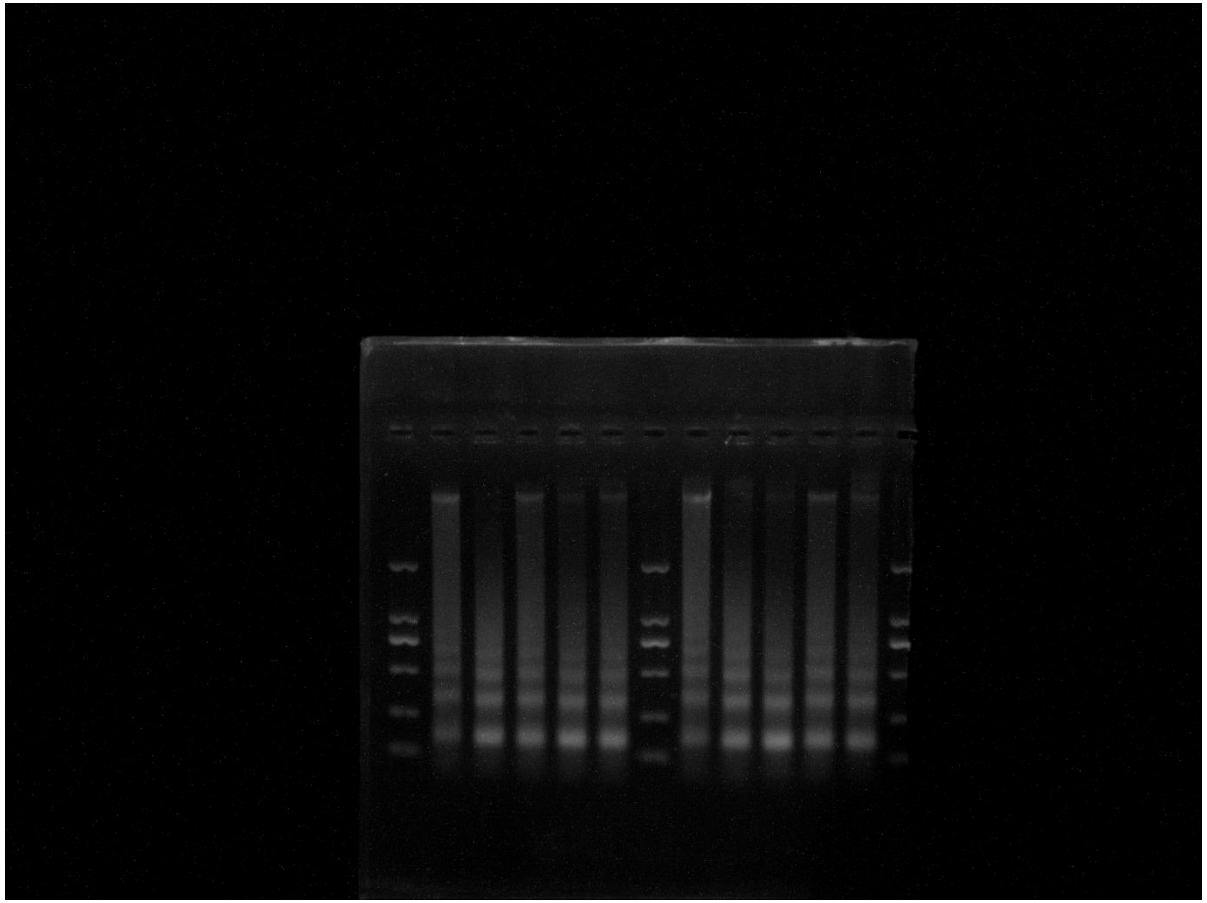

F3B

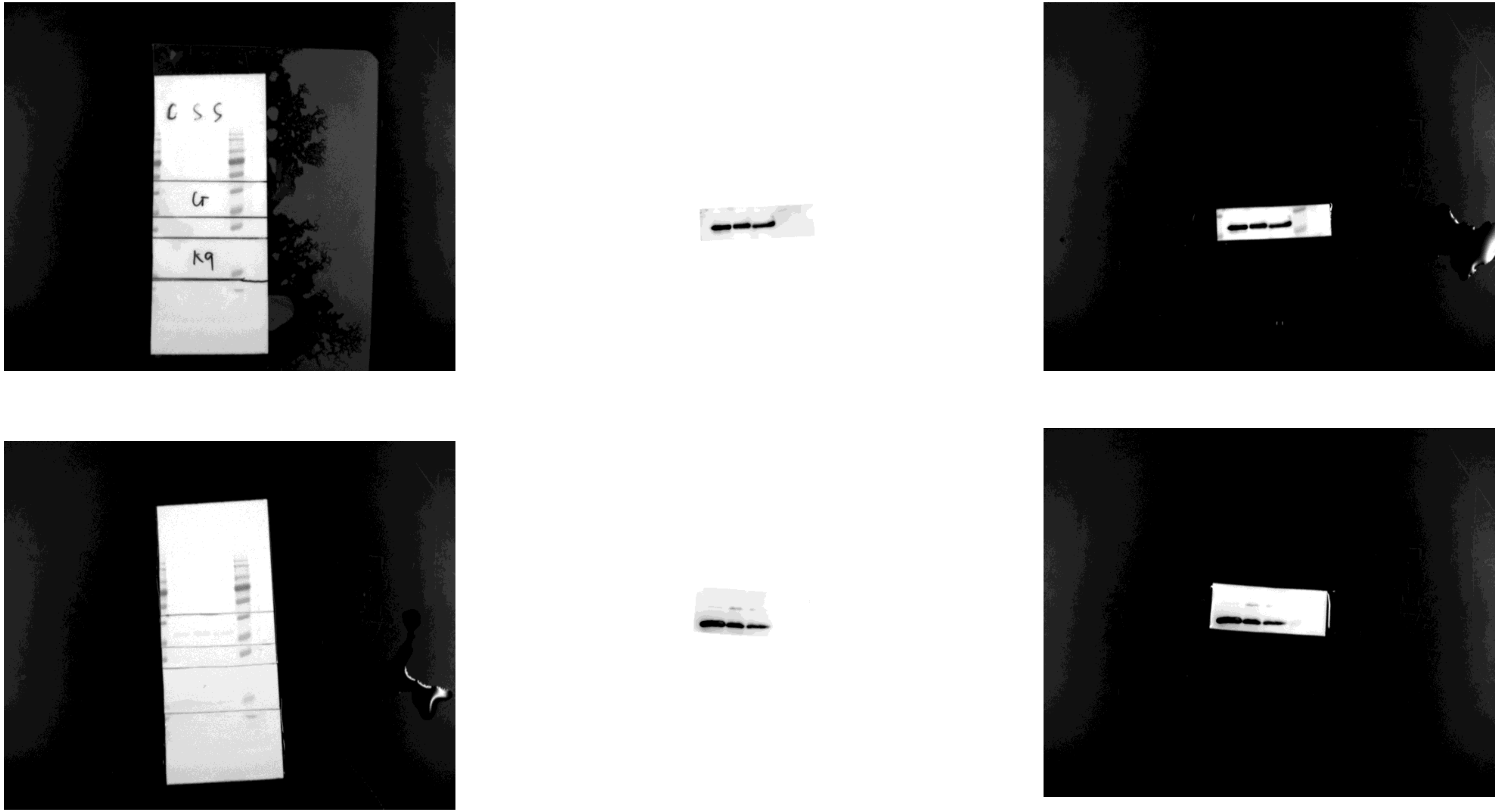

GAPDH

H3K9me3

F3B

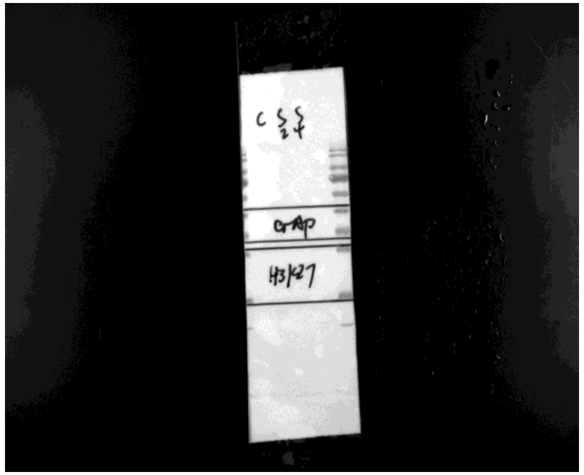

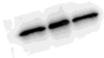

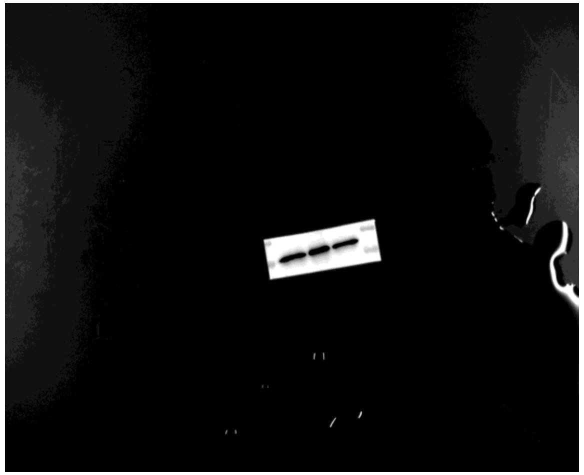

GAPDH

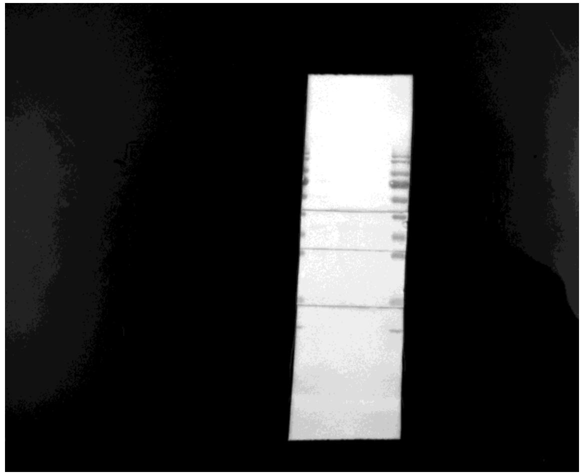

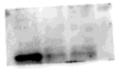

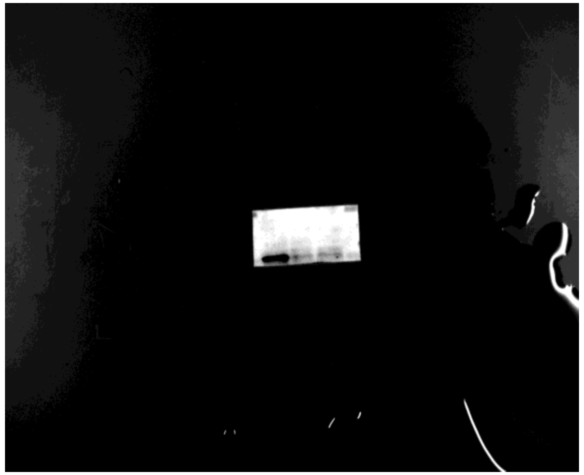

H3K27me3

F3B

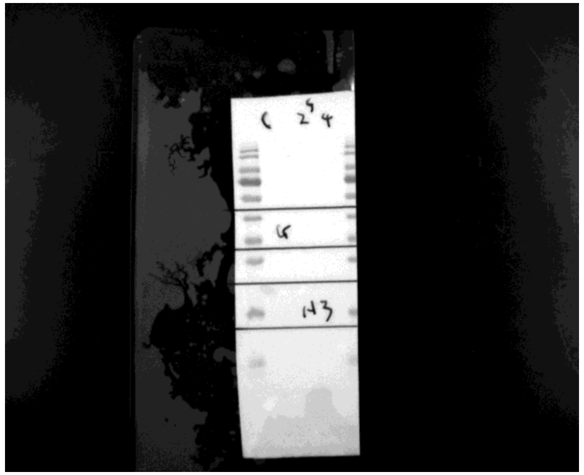

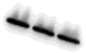

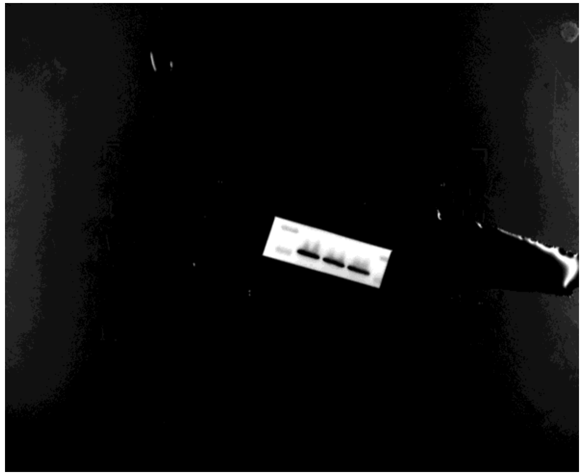

GAPDH

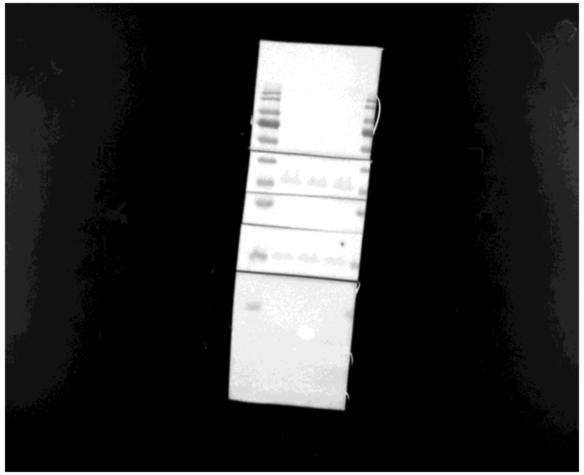

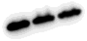

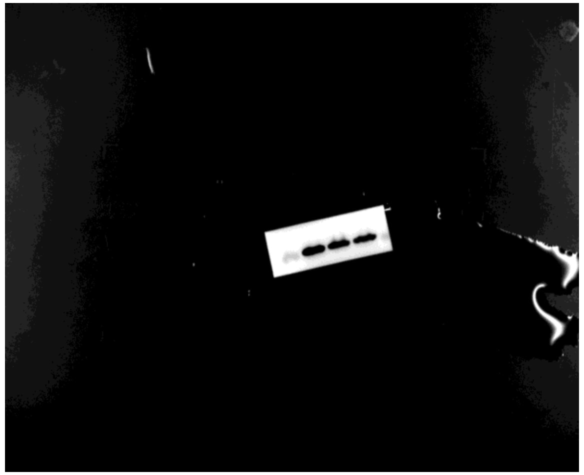

H3

F3C

GAPDH

gamaH2AX

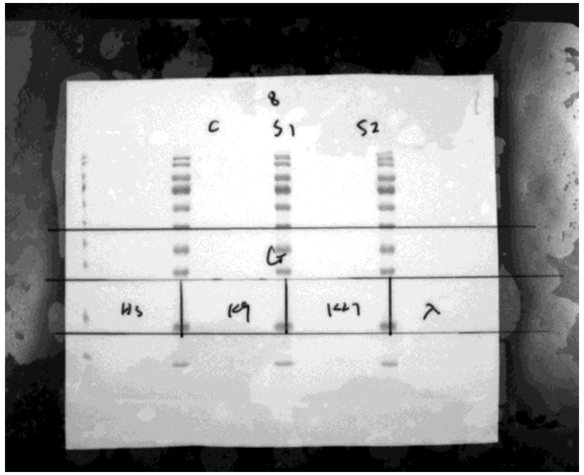

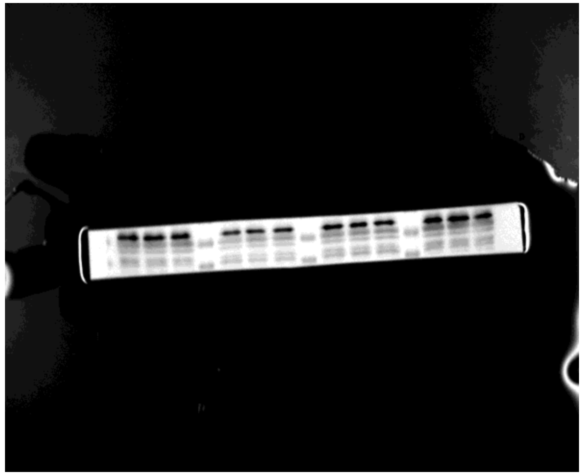

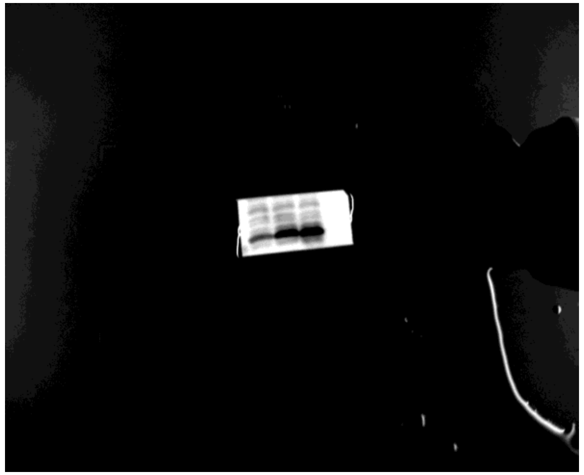

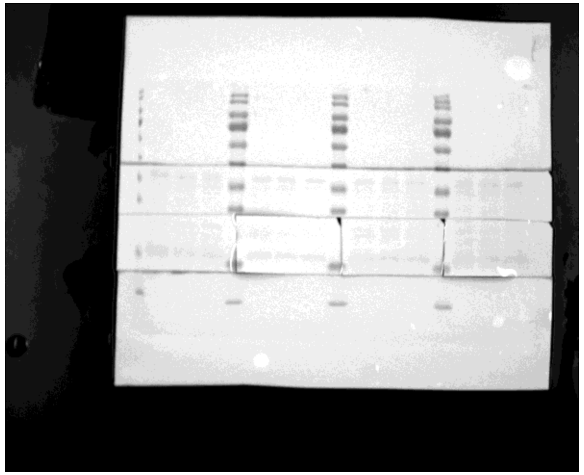

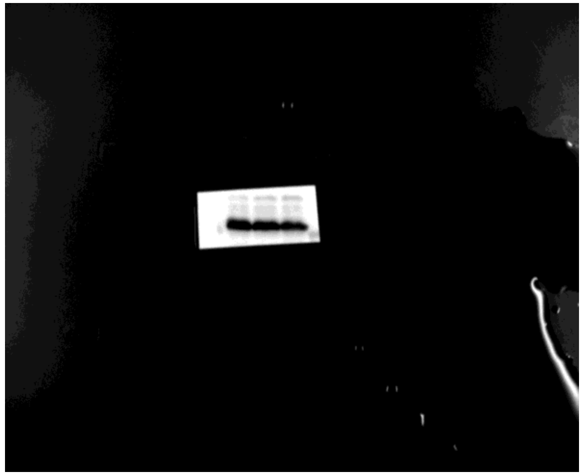

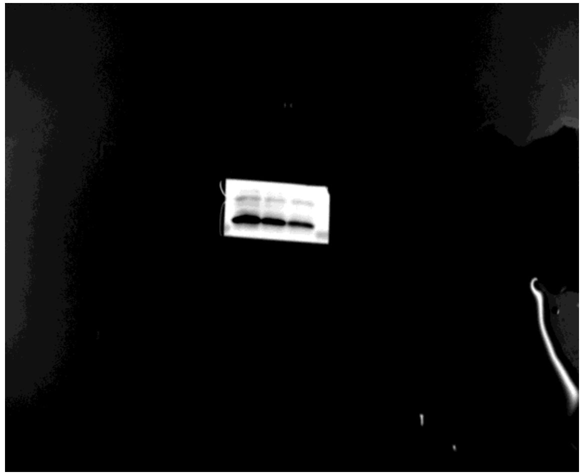

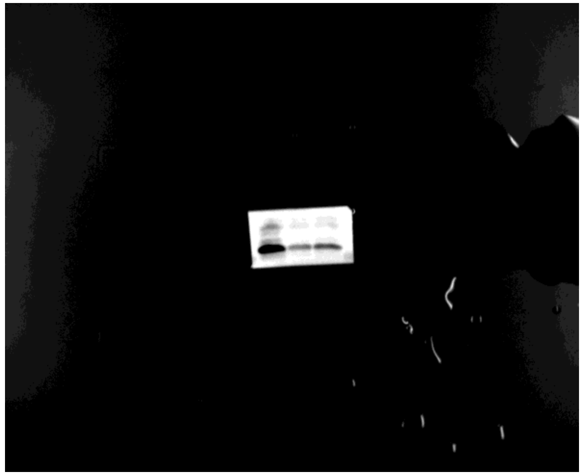

H3

H3K9me3

H3K27me3

F3D

Patu8988T GAPDH gamaH2AX

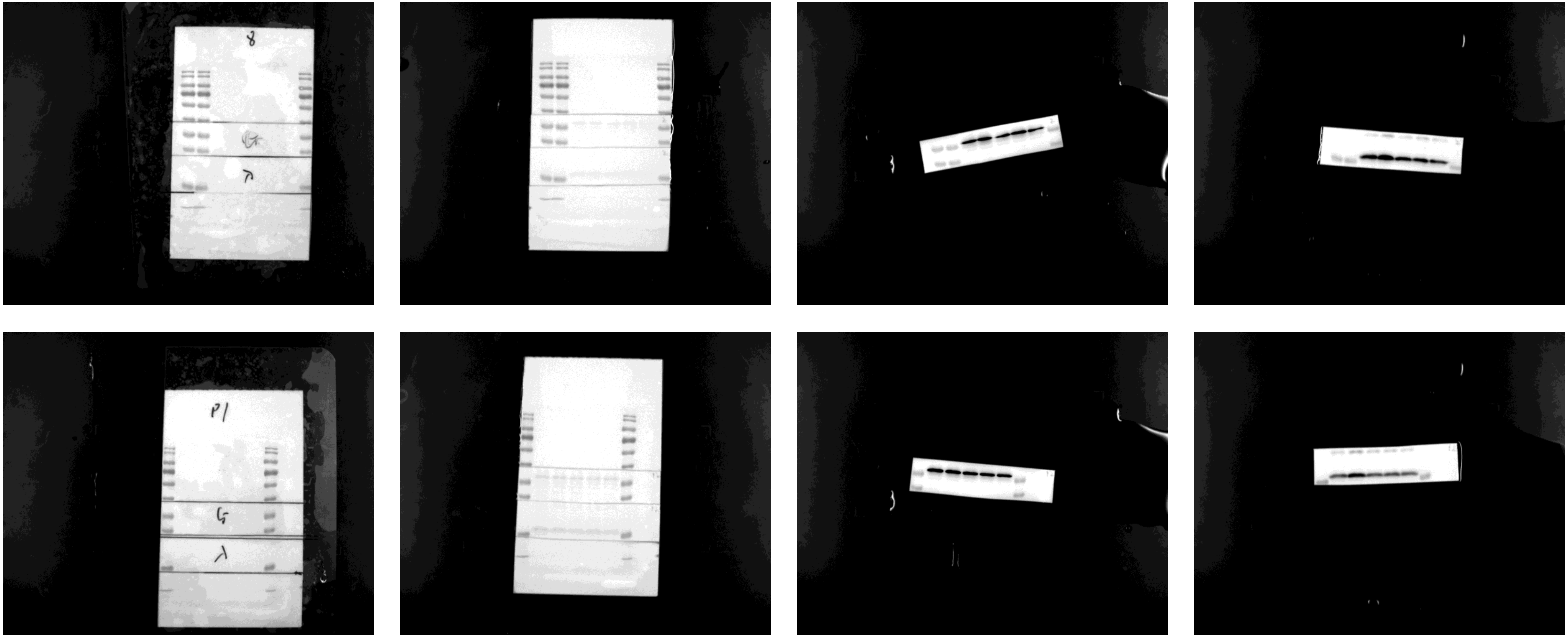

PANC1

F3D

Patu8988T GAPDH H1.0

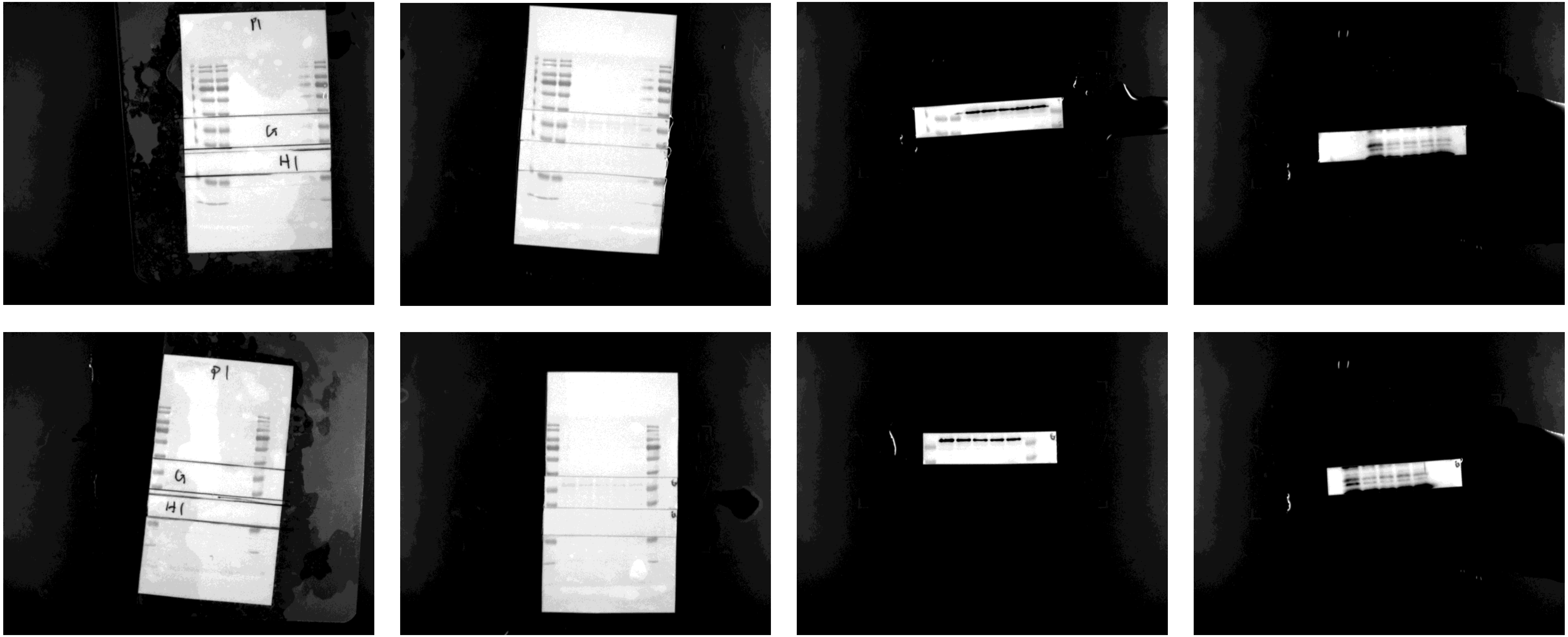

PANC1

F3D

Patu8988T

GAPDH

H3

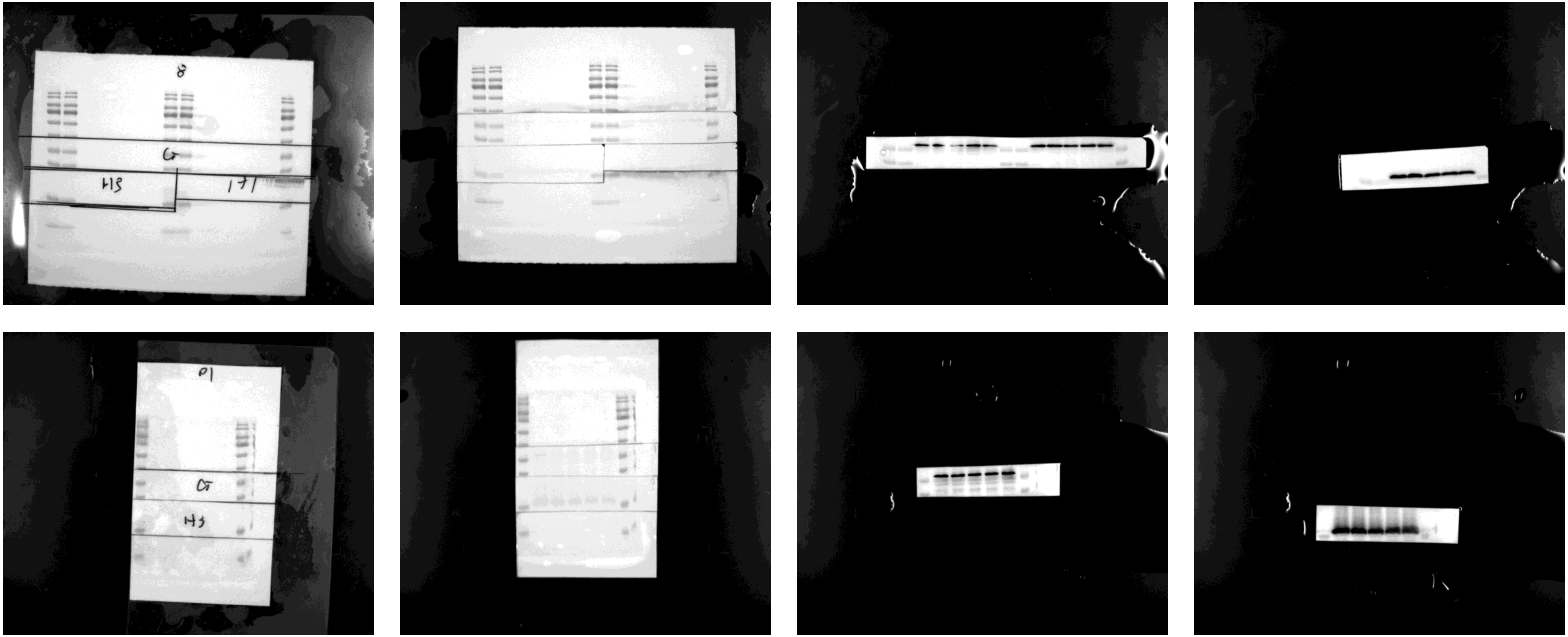

PANC1

F3D

Patu8988T

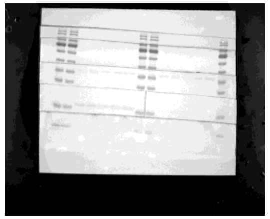

GAPDH

H3K9me3

H3K27me3

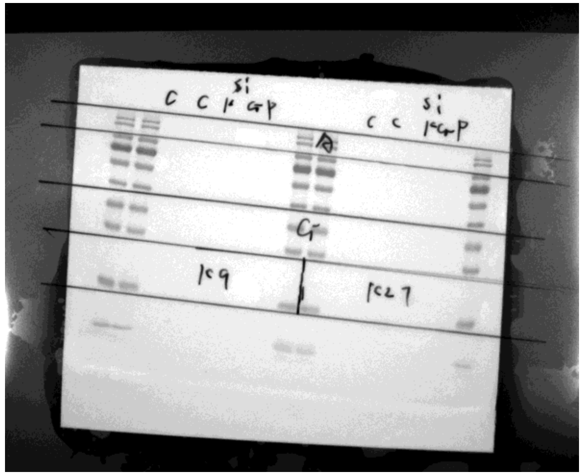

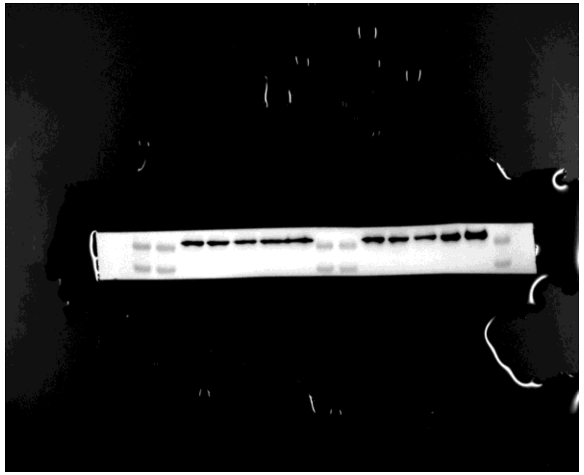

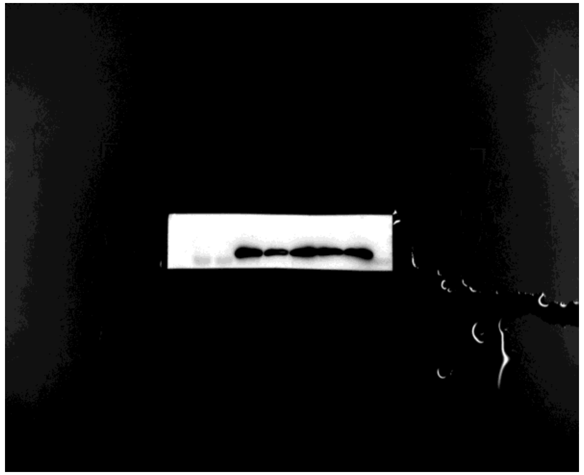

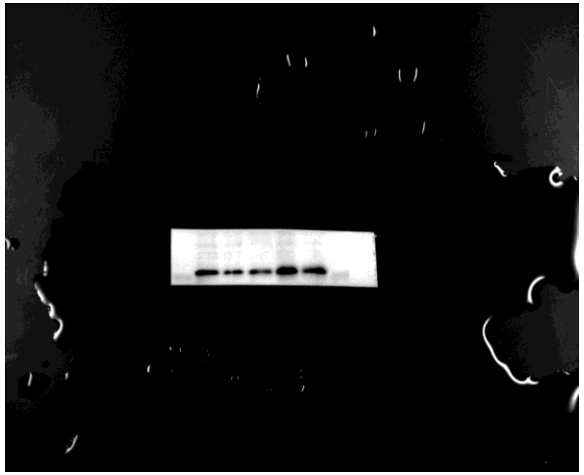

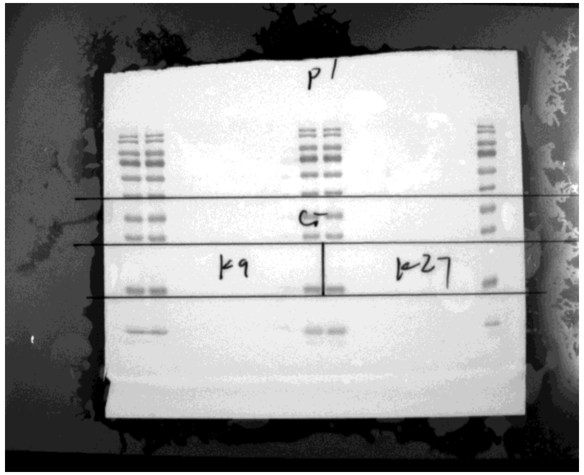

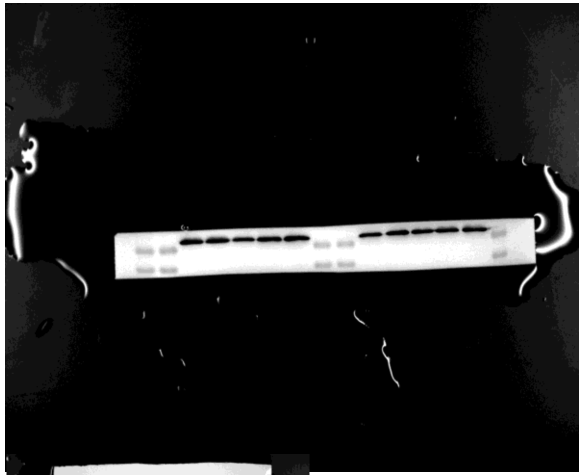

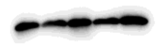

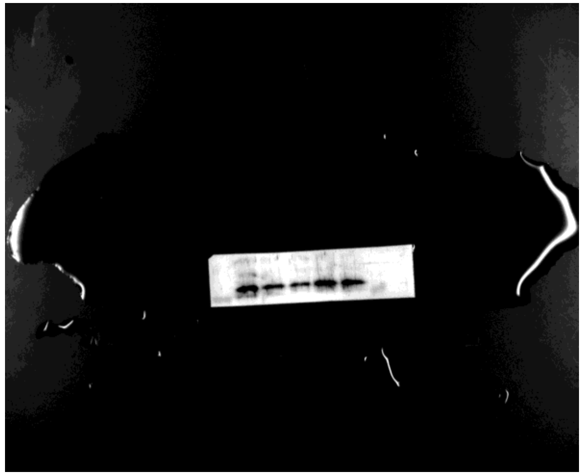

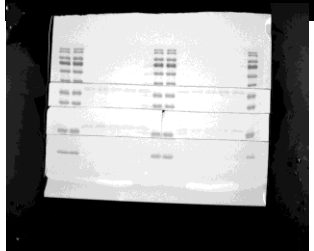

PANC1

F3F

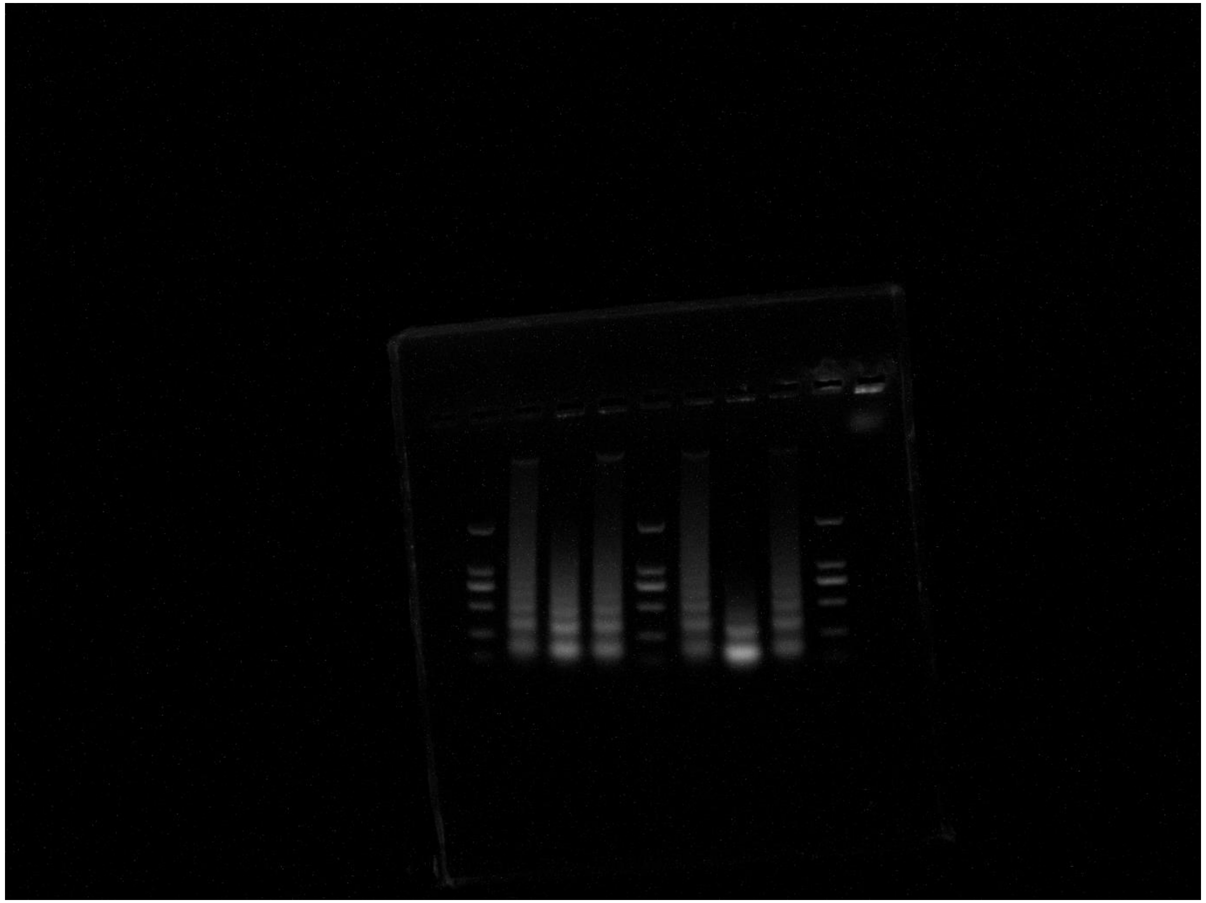

F3G

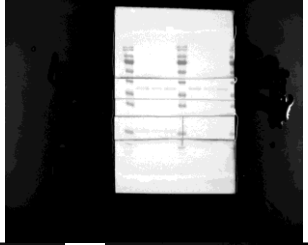

Patu8988T

GAPDH

H3K9me3

H3K27me3

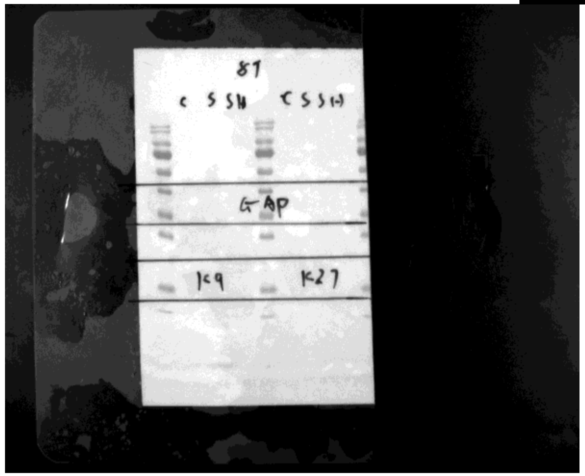

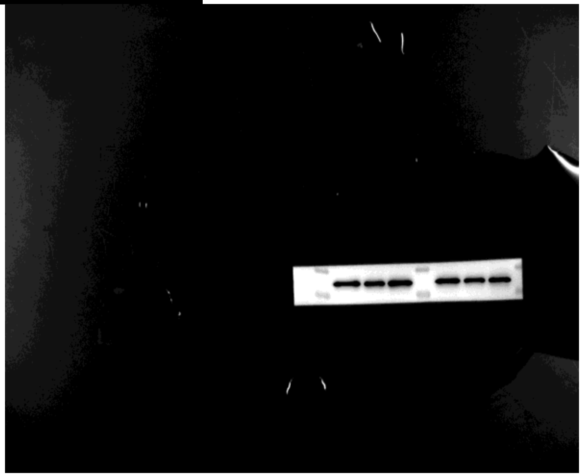

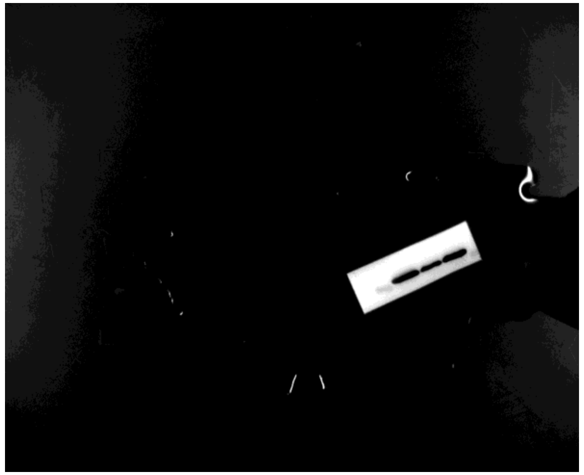

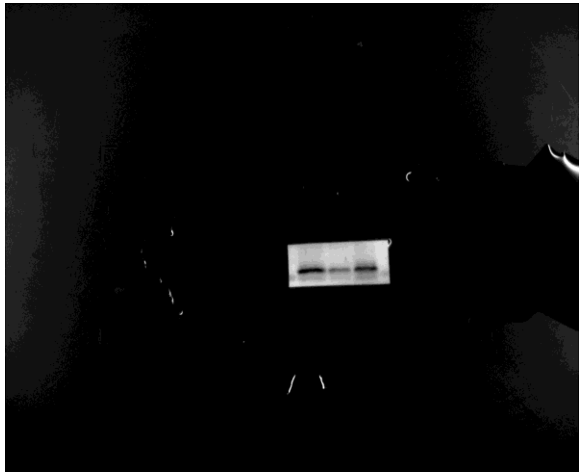

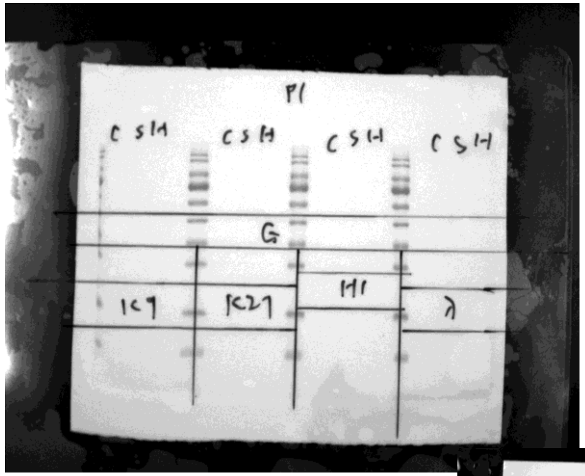

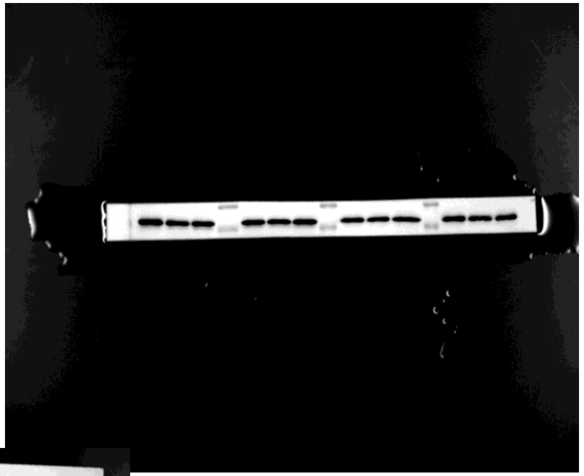

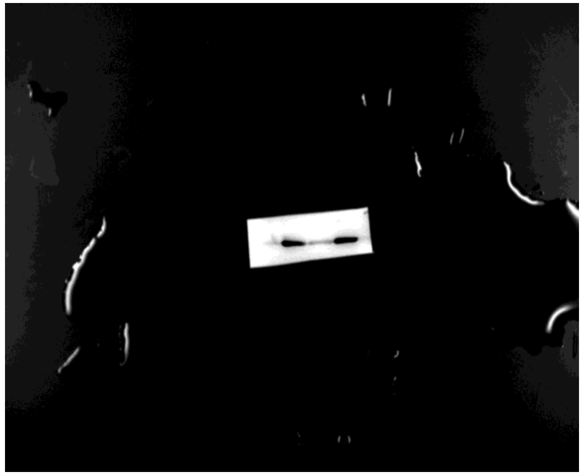

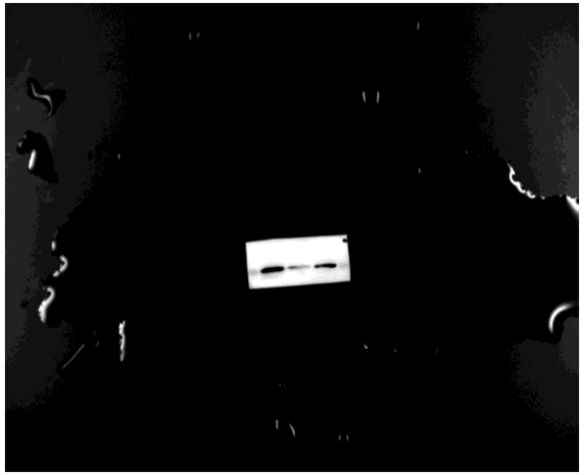

PANC1

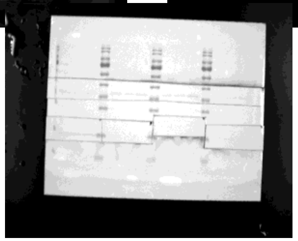

F4B

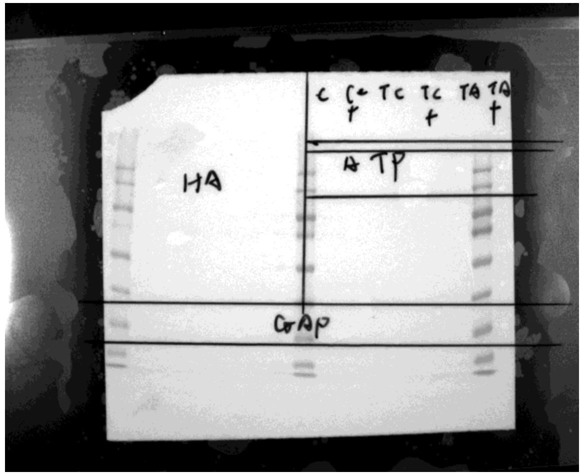

ATP2B4

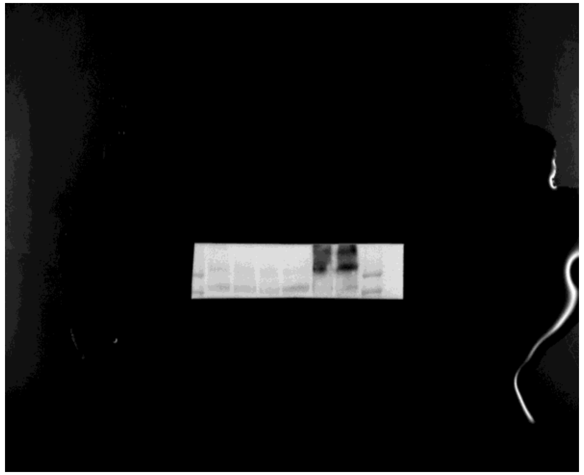

GAPDH

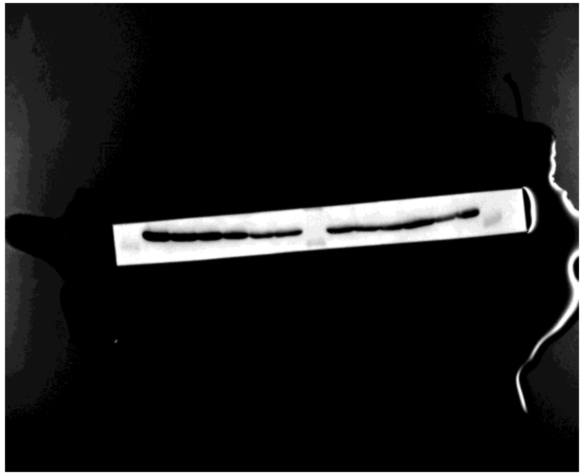

HA

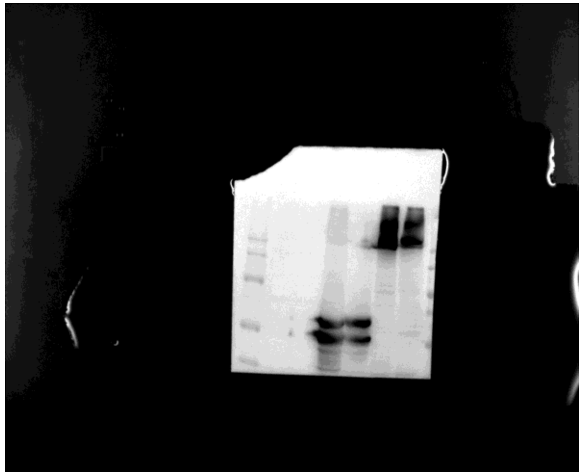

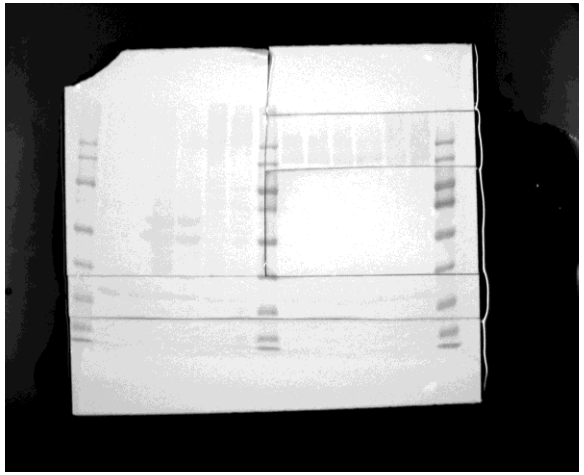

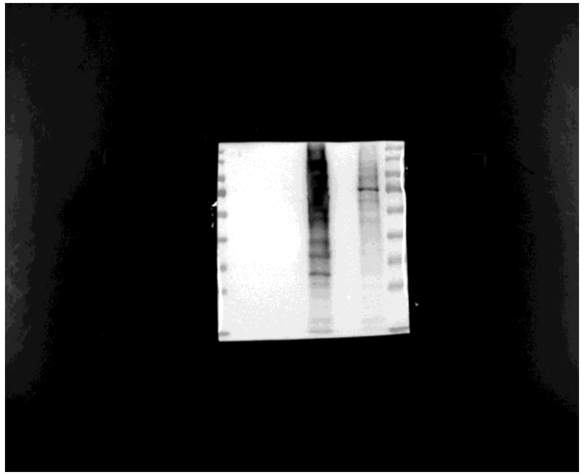

Sterp-HRP

F4E

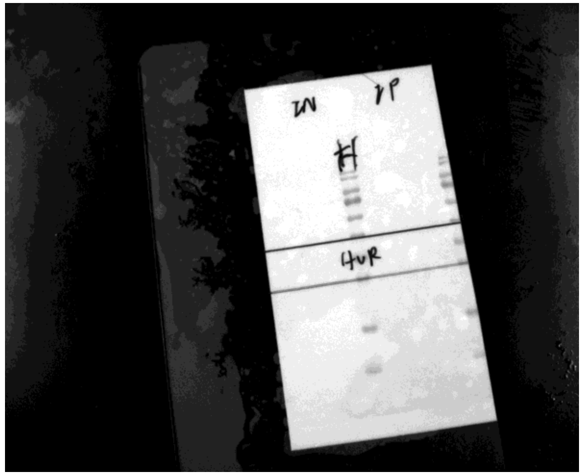

HA

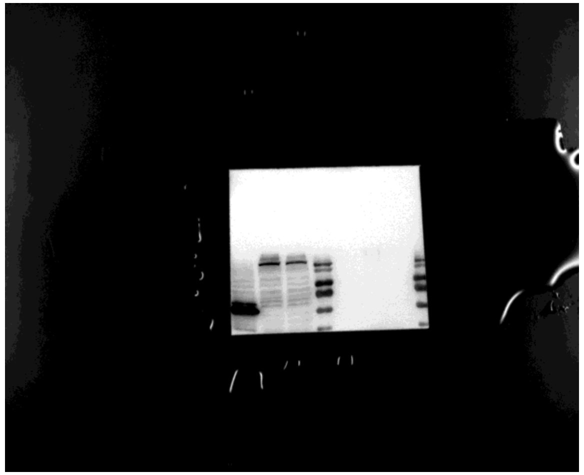

ELAVL1
(short exposure time)

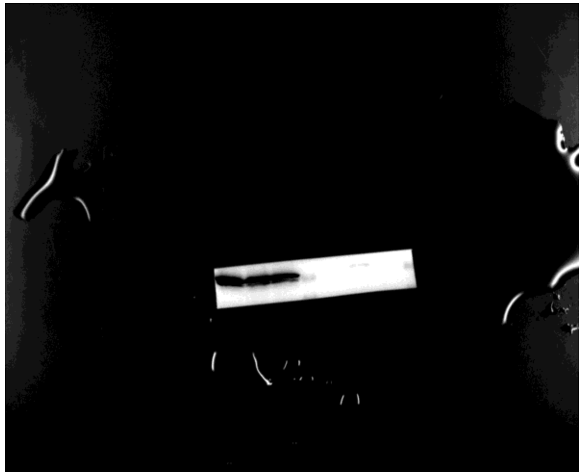

ELAVL1
(long exposure time)

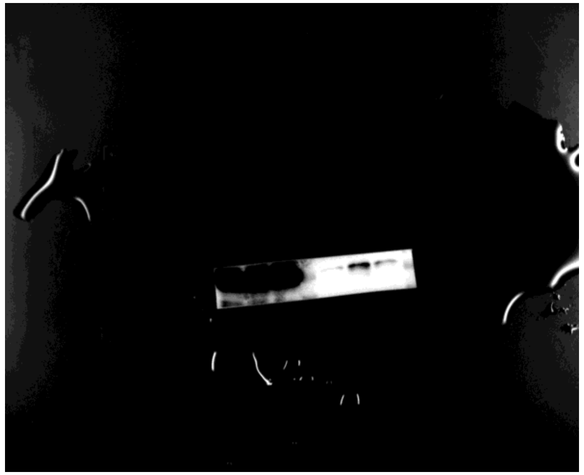

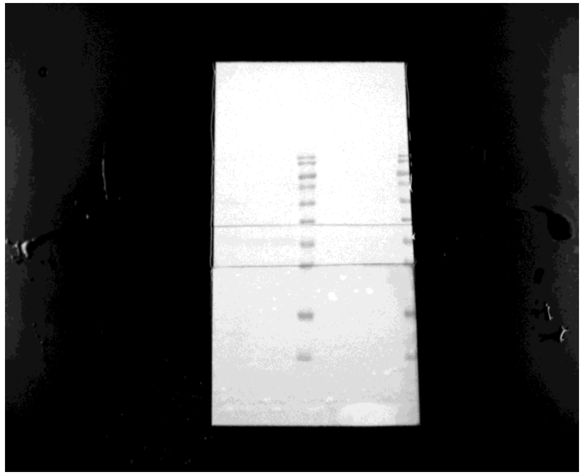

F4F

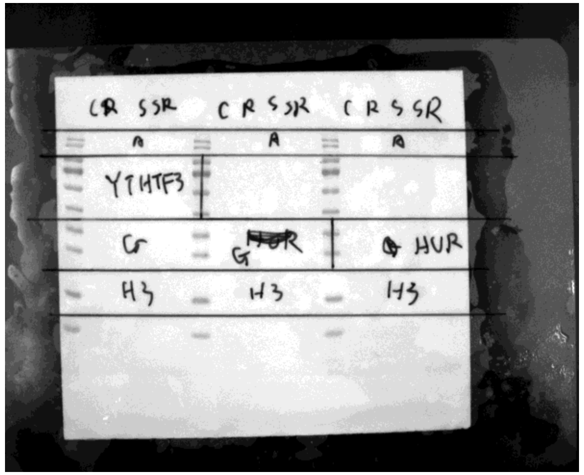

ATP2B4

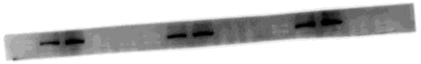

GAPDH

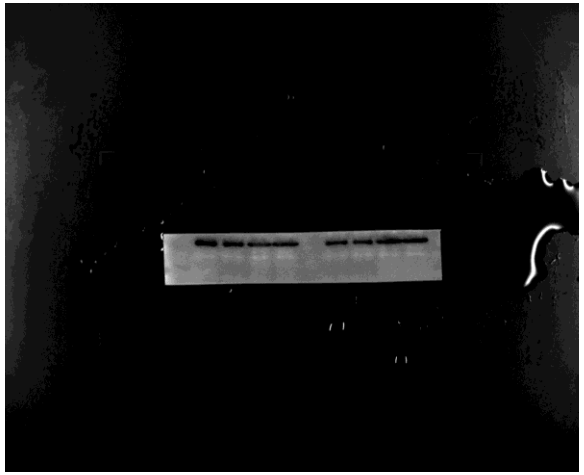

ELAVL1

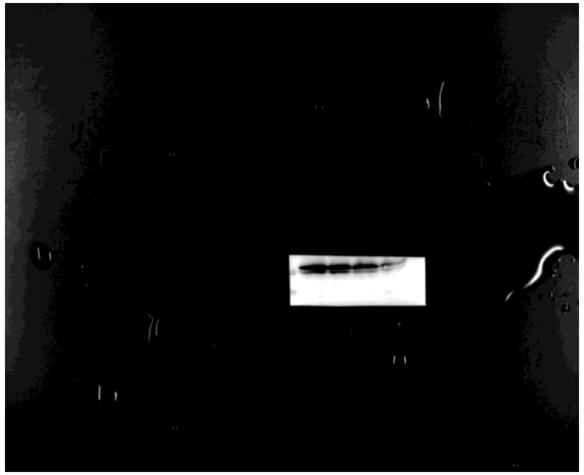

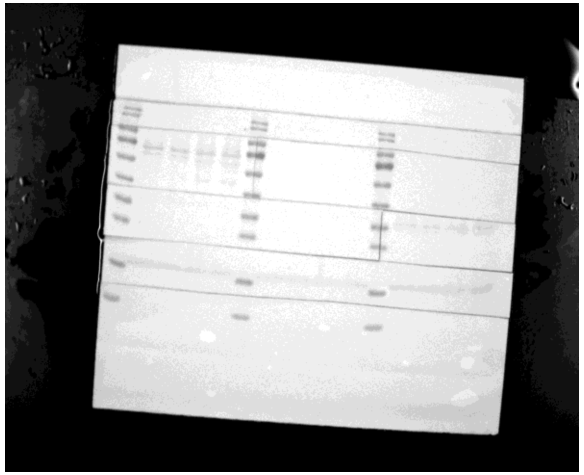

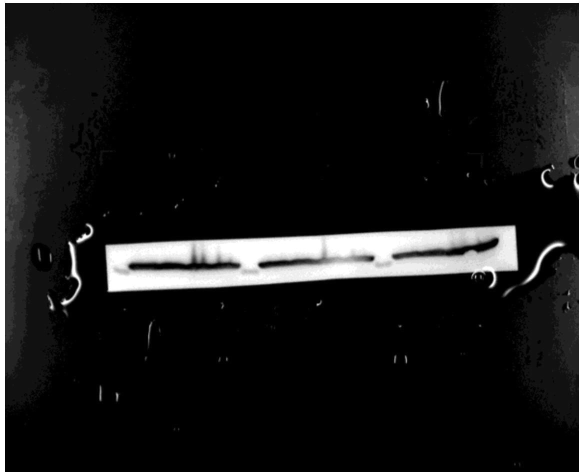

H3

F4G

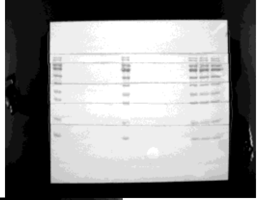

ATP2B4

GAPDH

H3

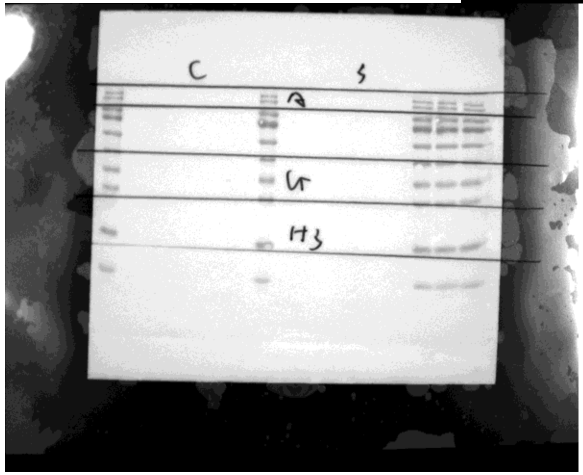

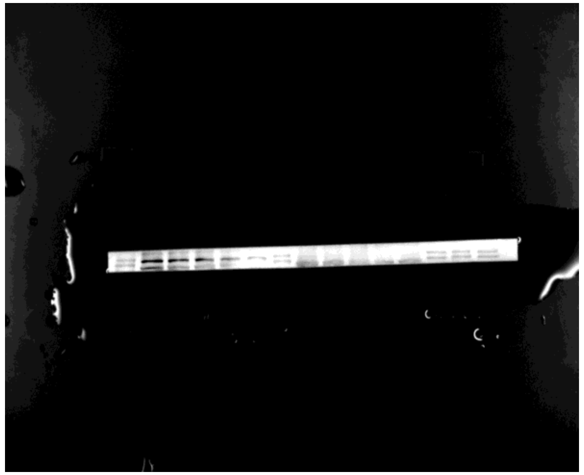

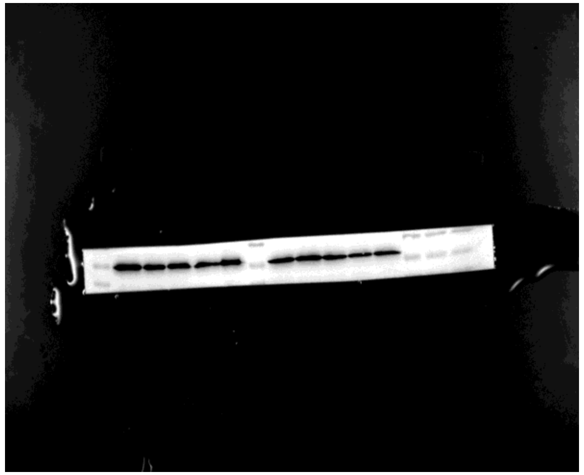

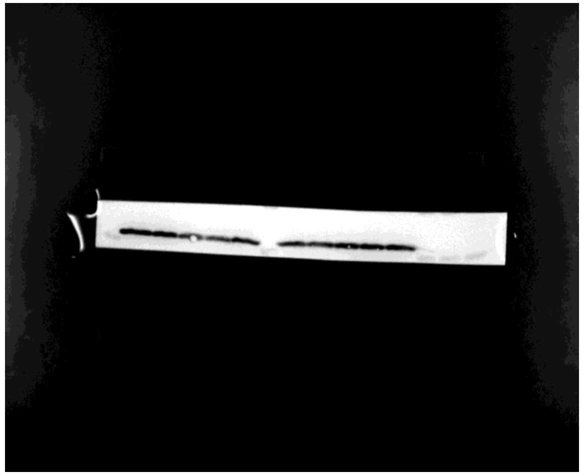

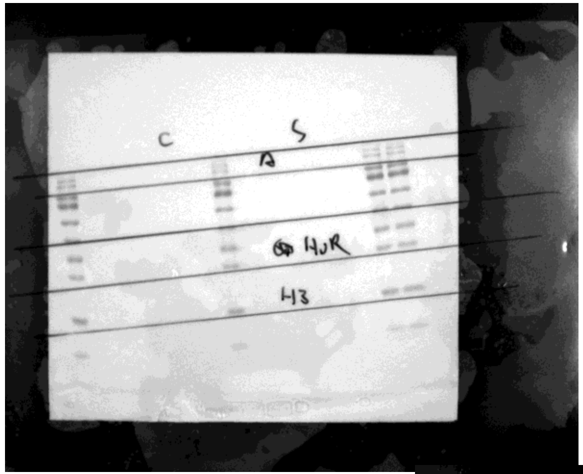

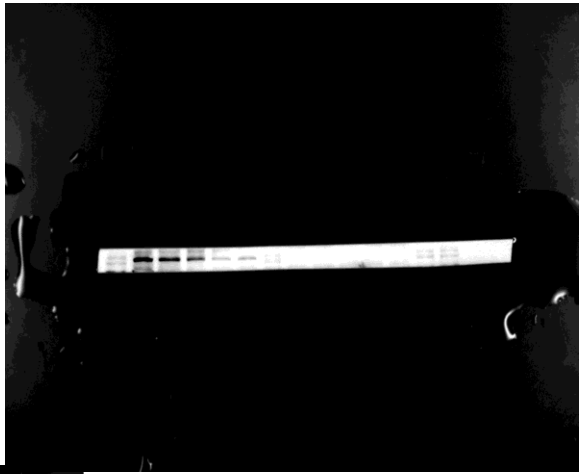

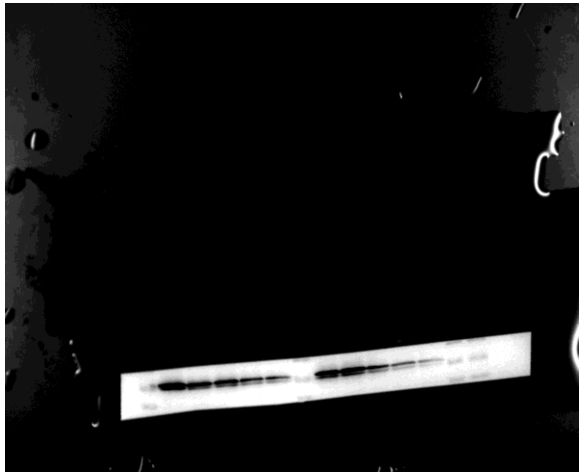

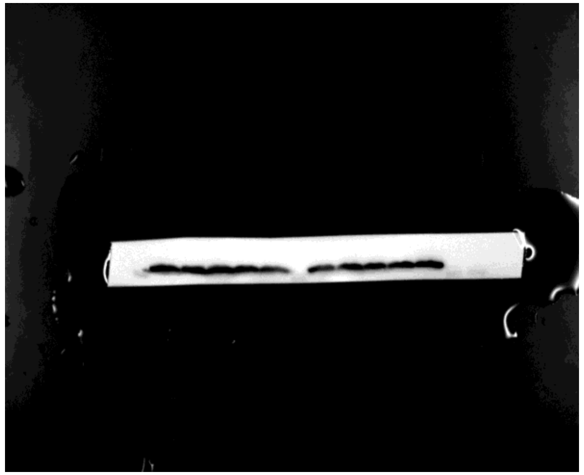

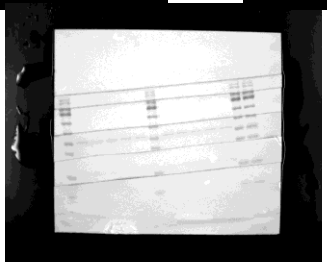

ATP2B4

ELAVL1

H3

F4K

ATP2B4 GAPDH Gama-H2AX

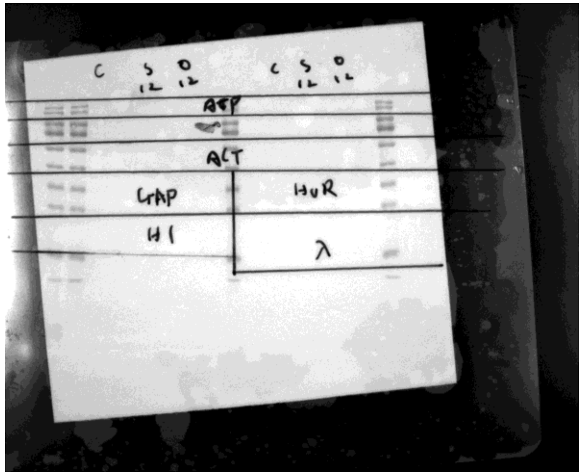

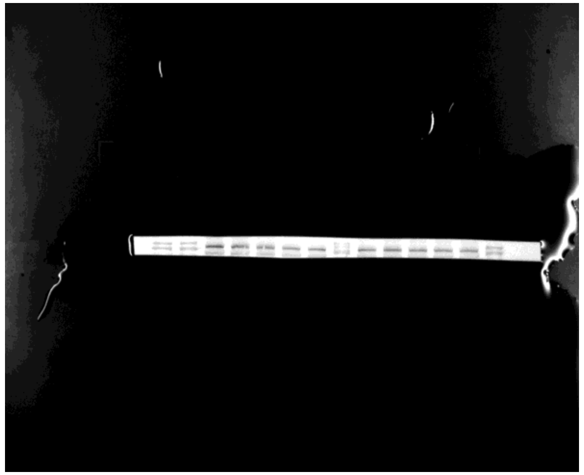

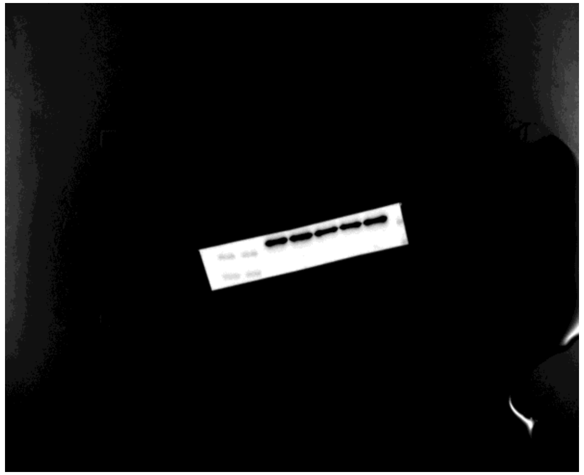

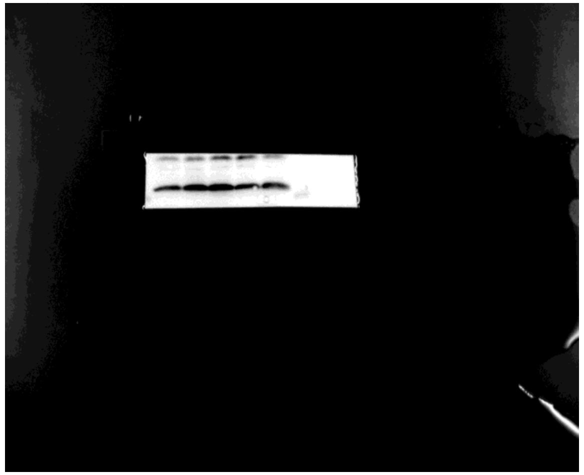

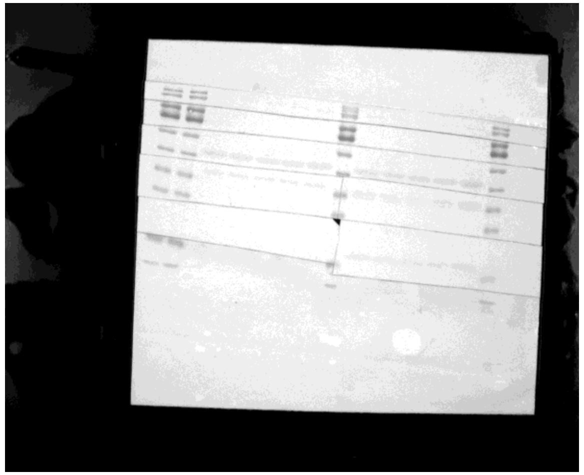

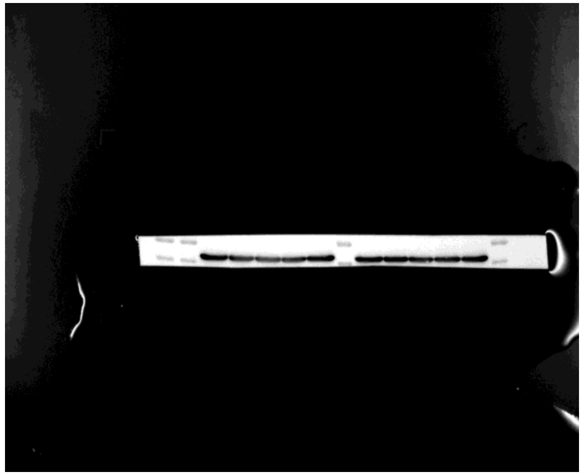

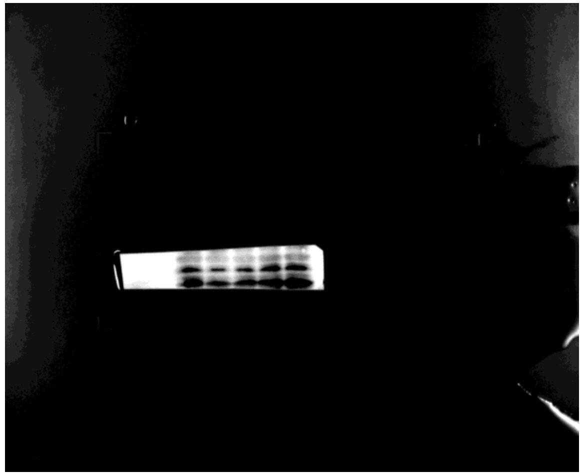

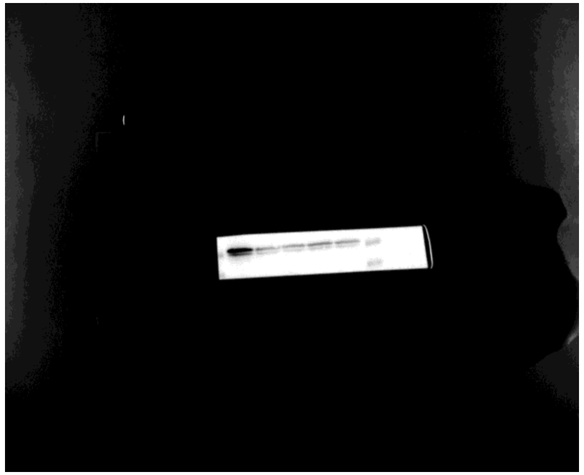

Beta-actin H1.0 ELAVL1

F4K

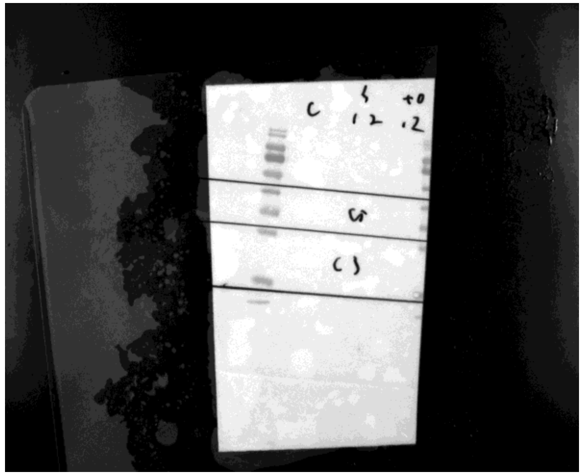

Cleaved-Caspase3

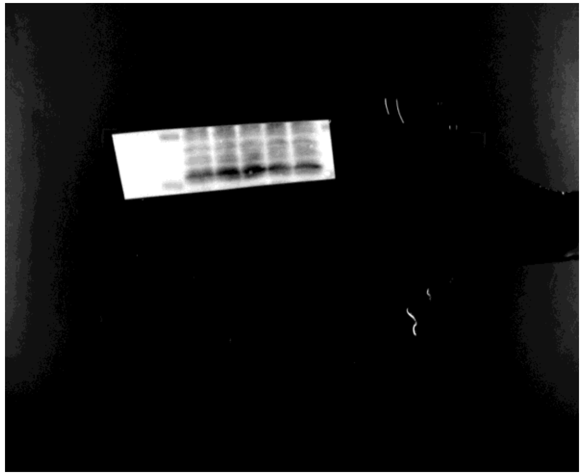

GAPDH

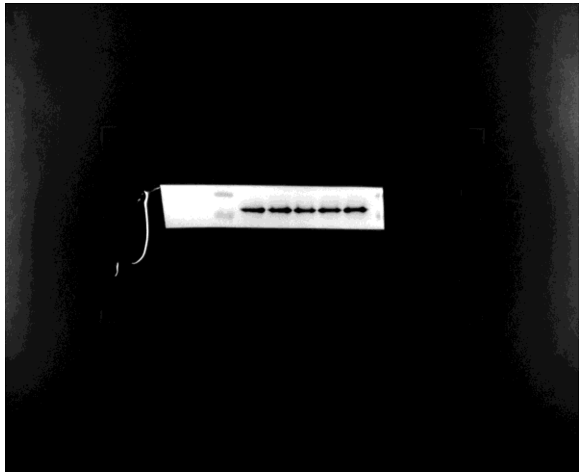

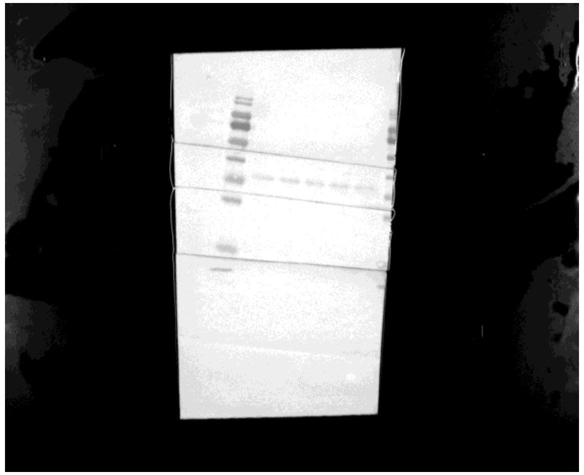

F5E

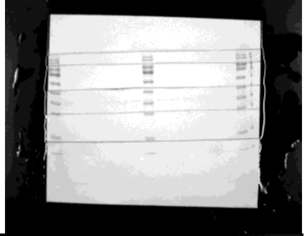

ATP2B4

H3

ELAVL1

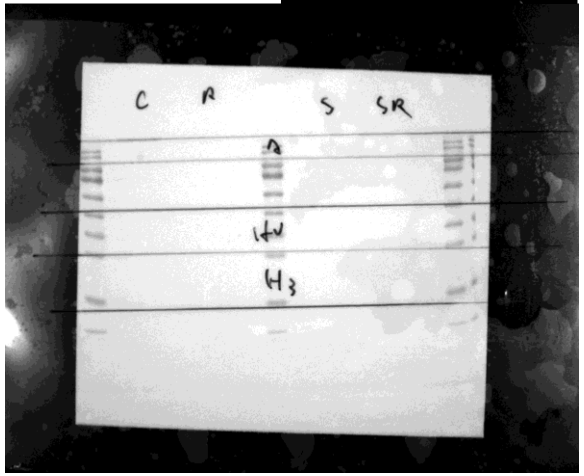

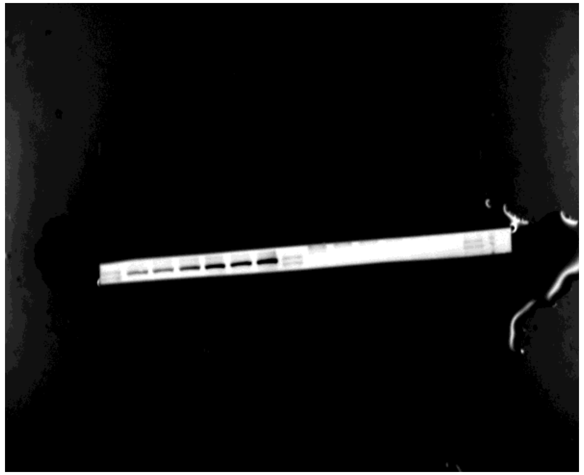

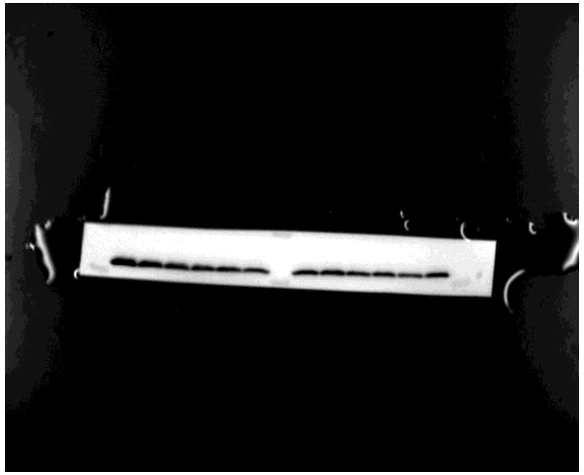

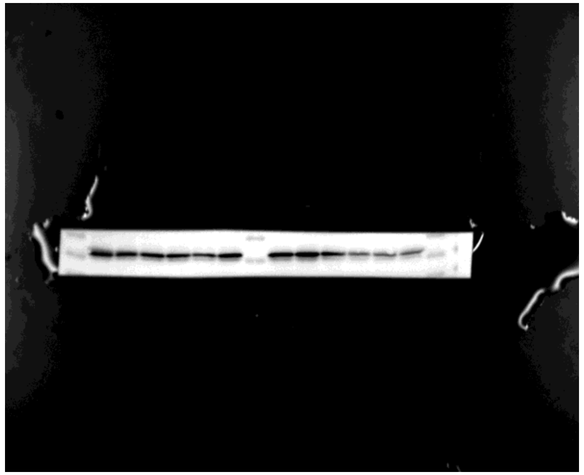

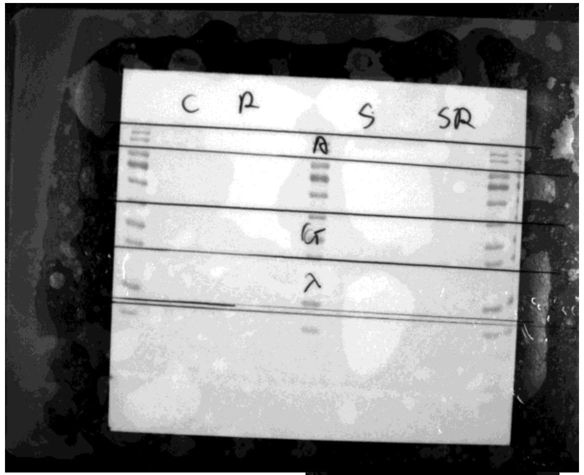

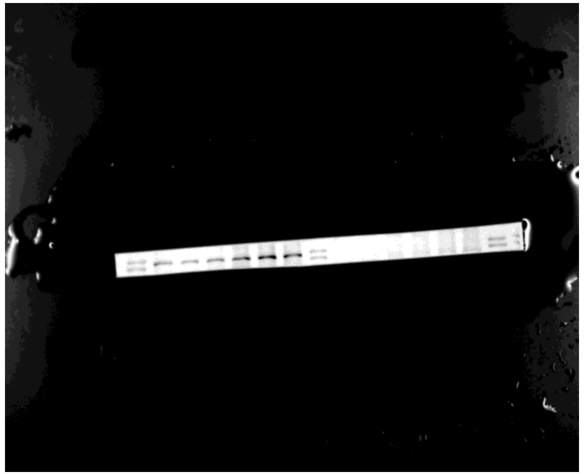

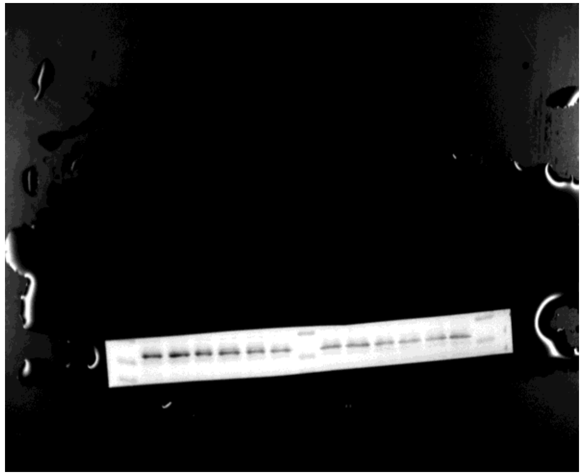

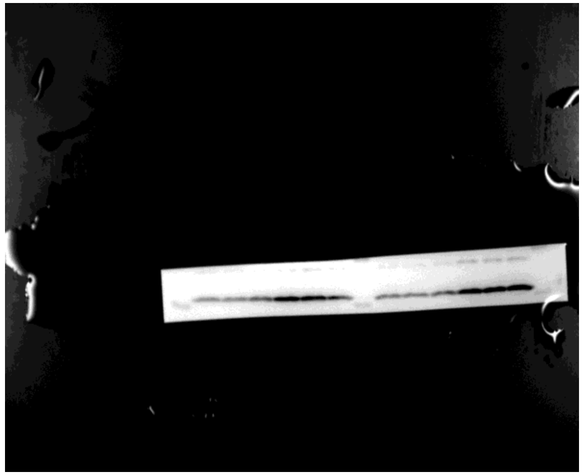

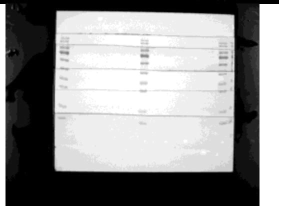

ATP2B4

GAPDH

GAMA-H2AX

F5E

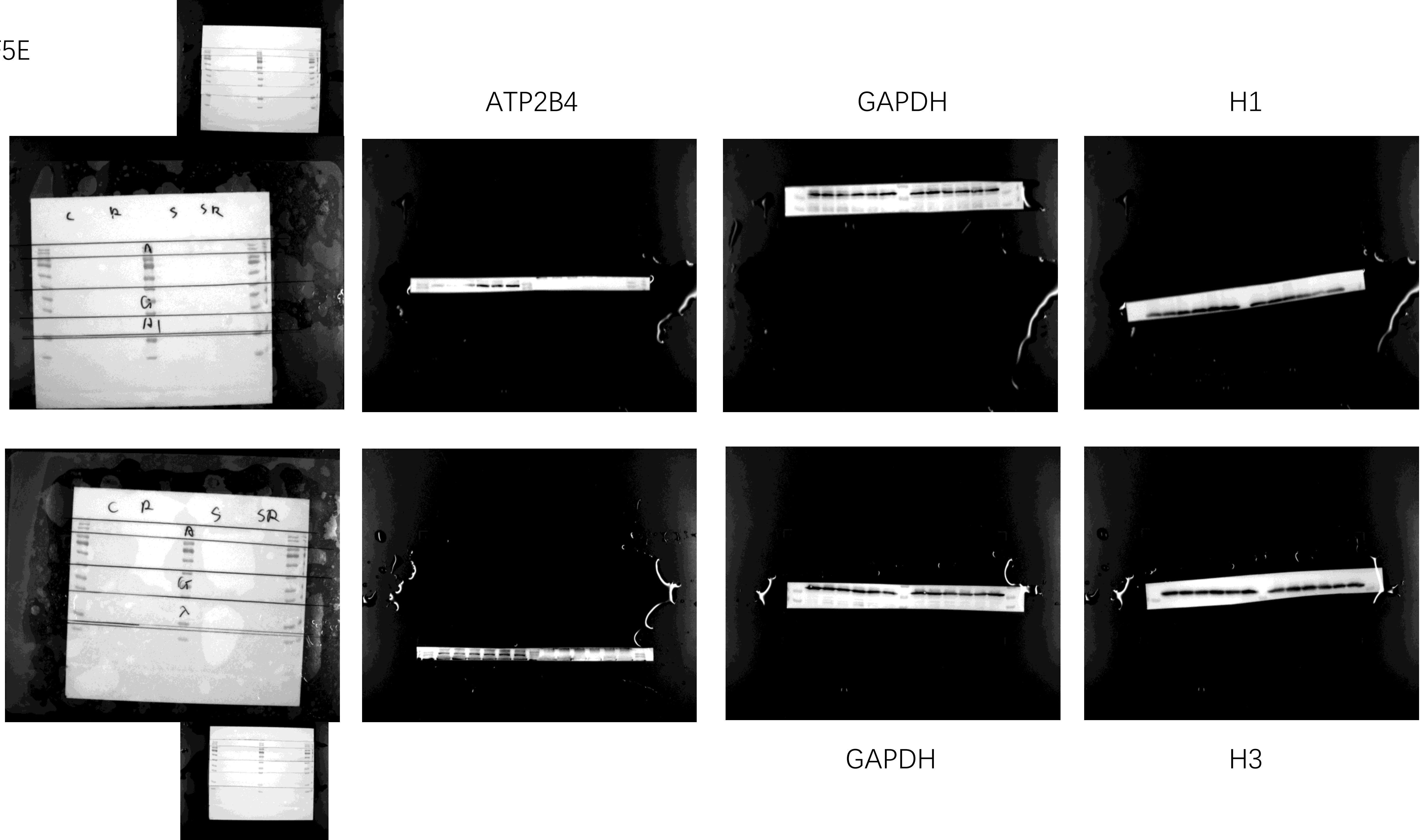

FS1C

ATP2B4

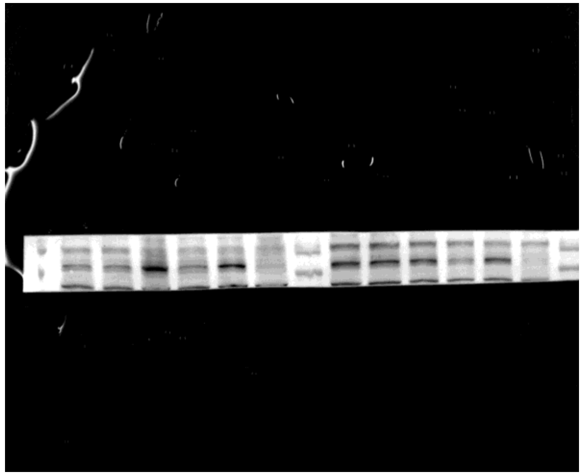

GAPDH

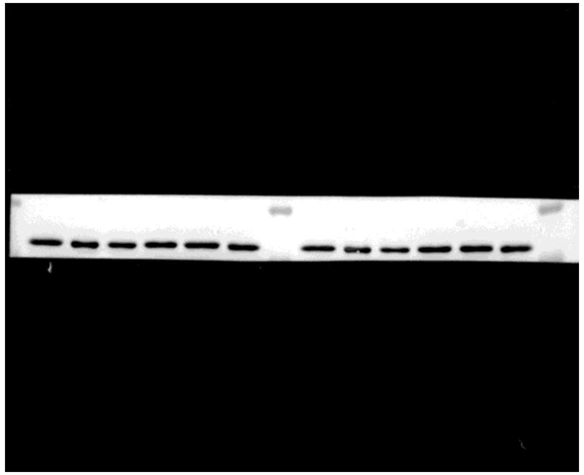

FS1E

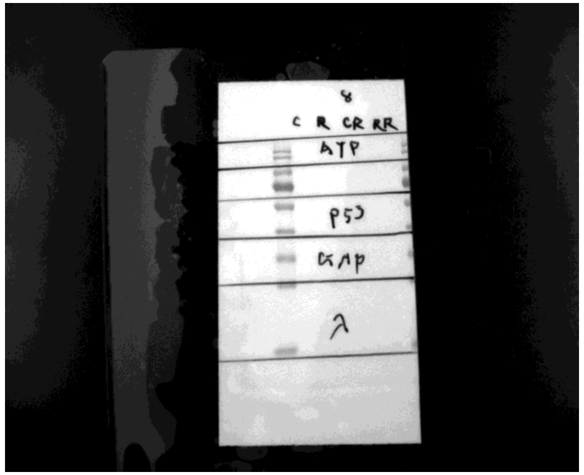

ATP2B4

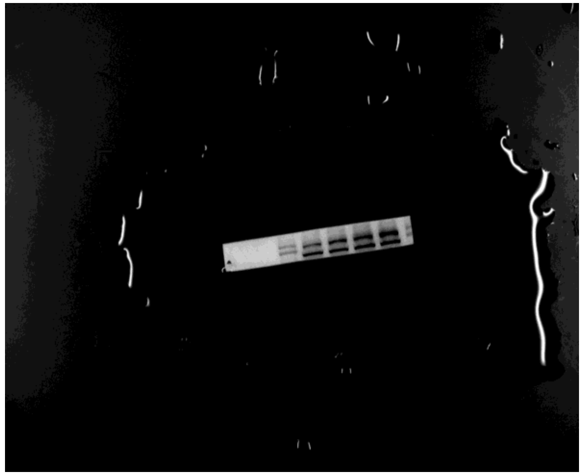

GAPDH

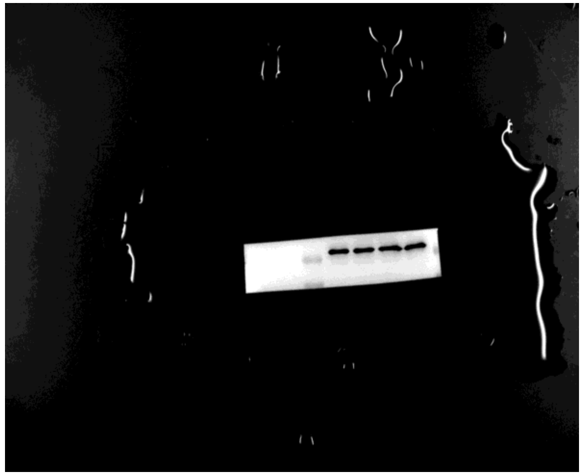

Gama-H2AX

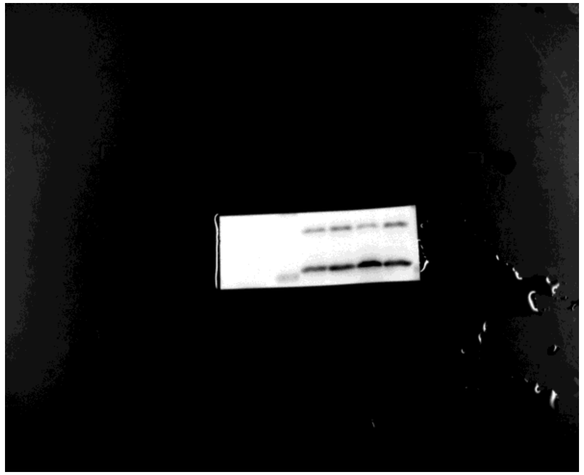

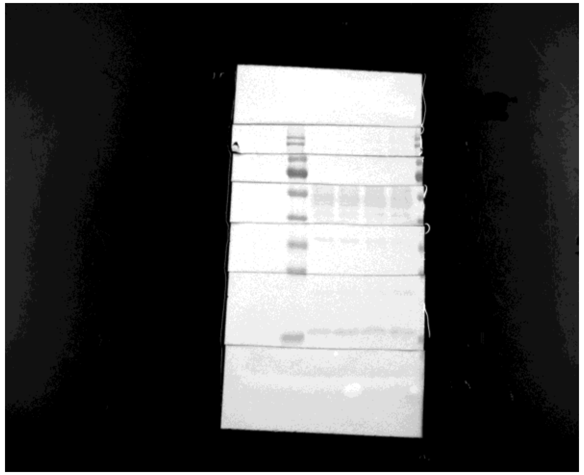

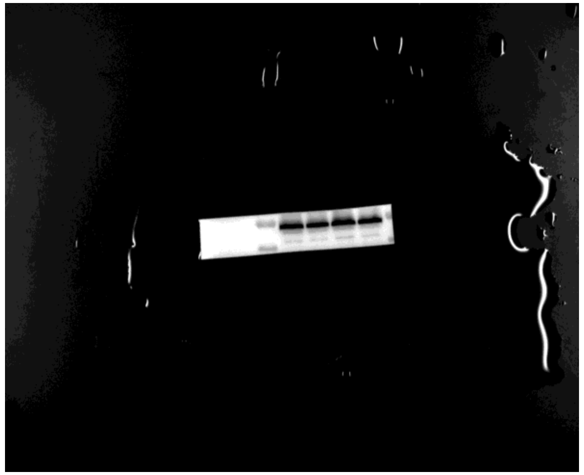

P53

FS1J

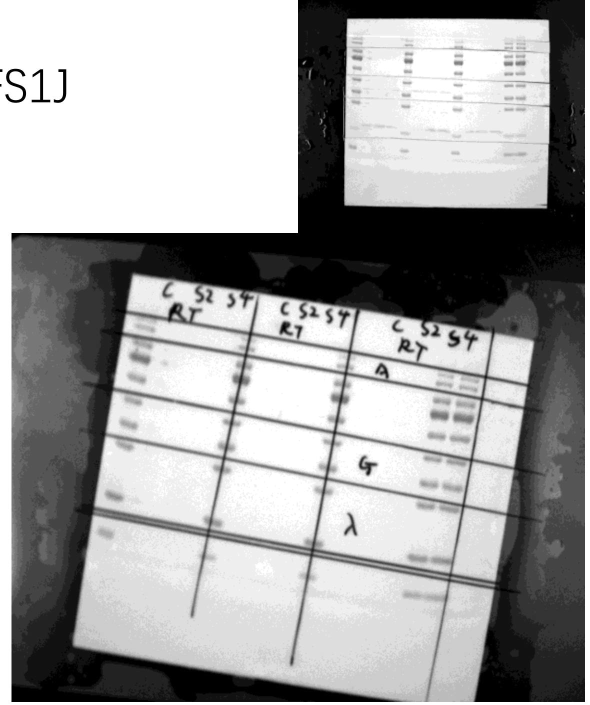

ATP2B4

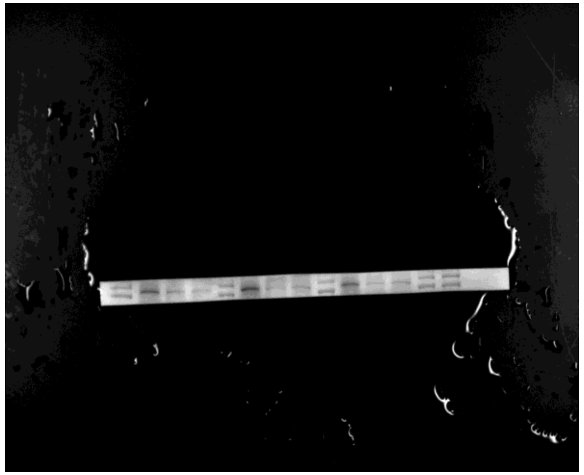

GAPDH

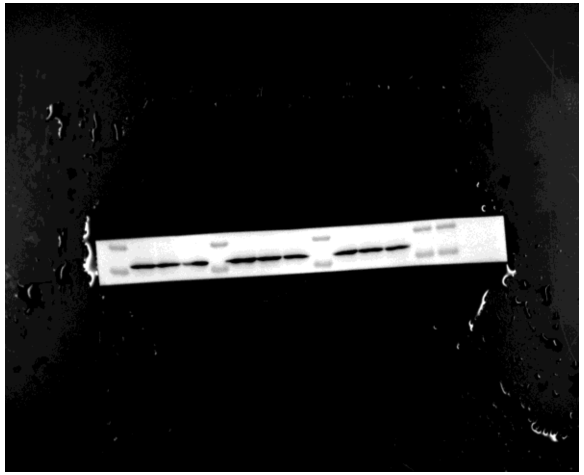

Gama-H2AX

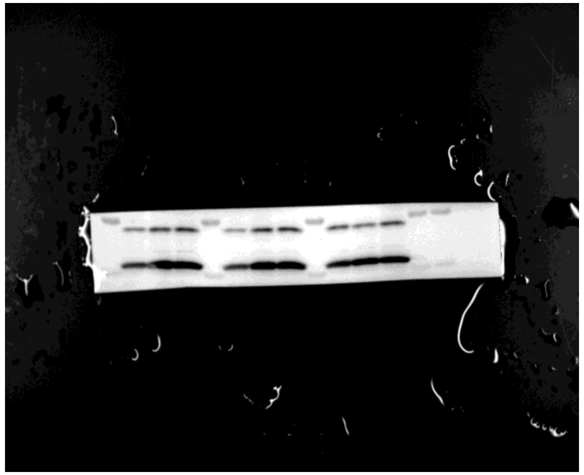

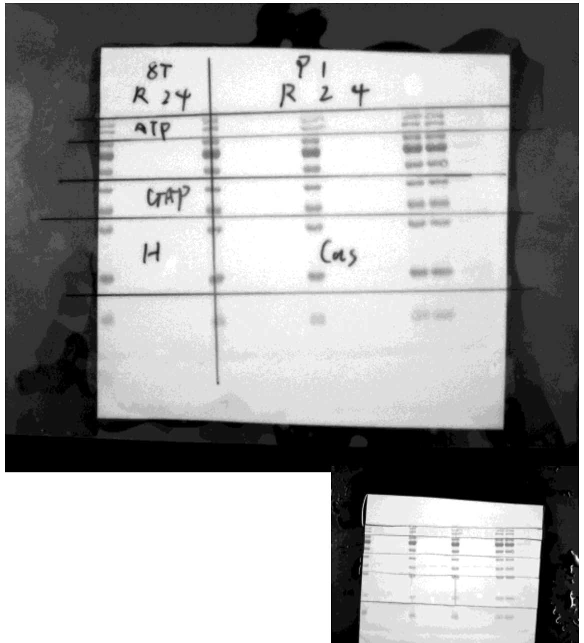

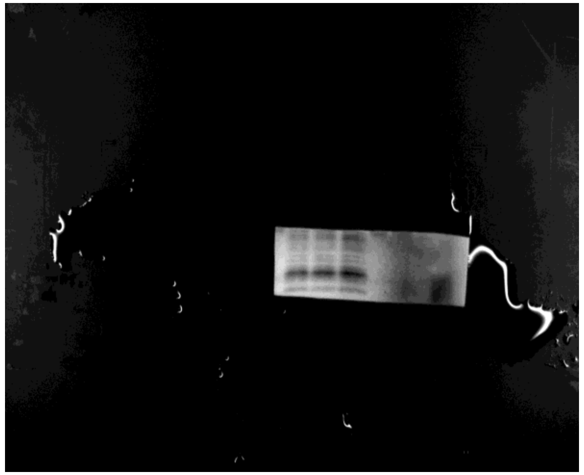

Cleaved-Caspase3

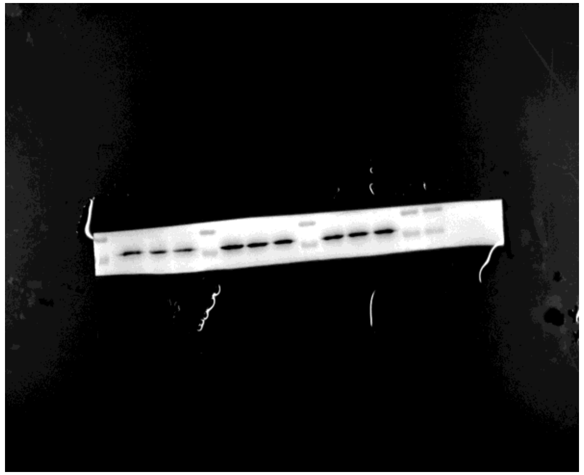

FS1K

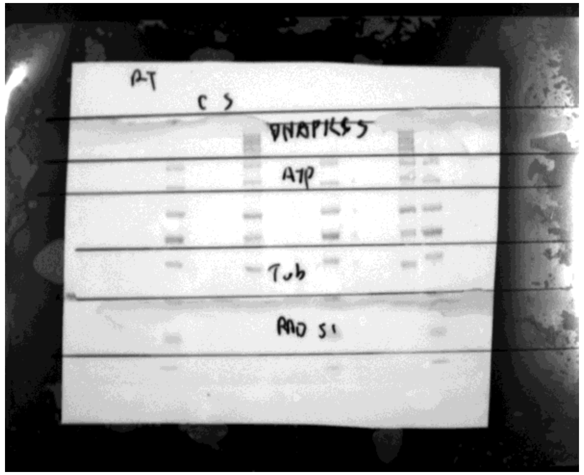

ATP2B4

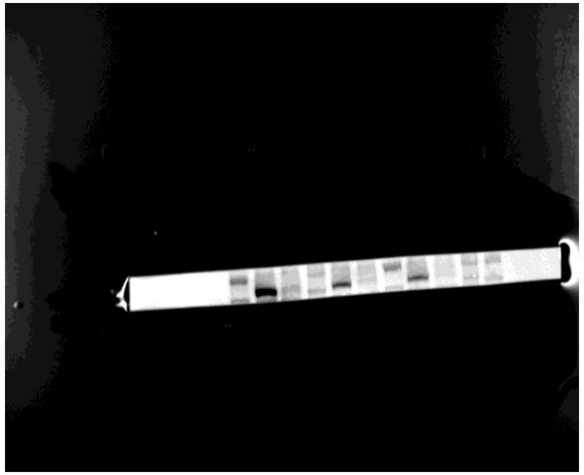

DNA-Pkcs

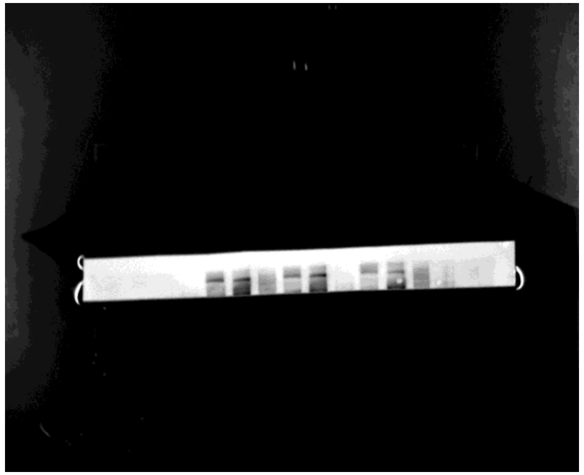

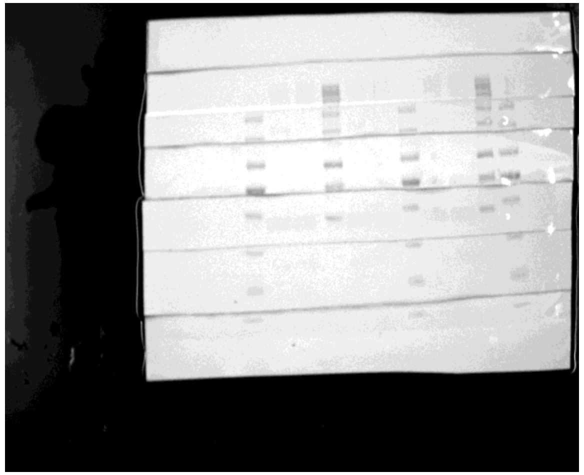

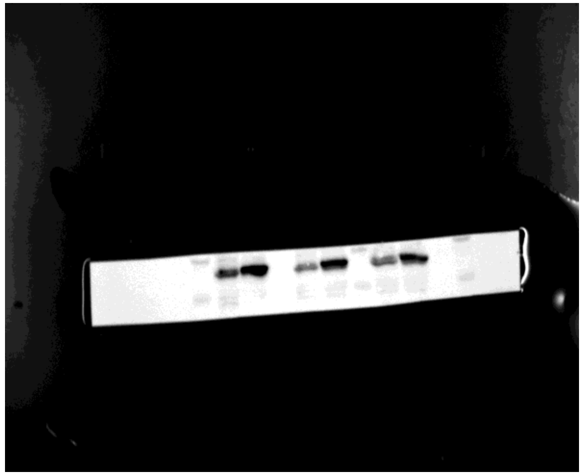

Rad51

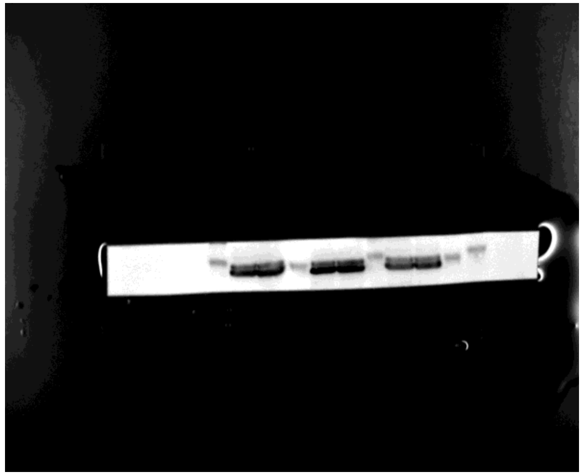

Tublin

FS2F

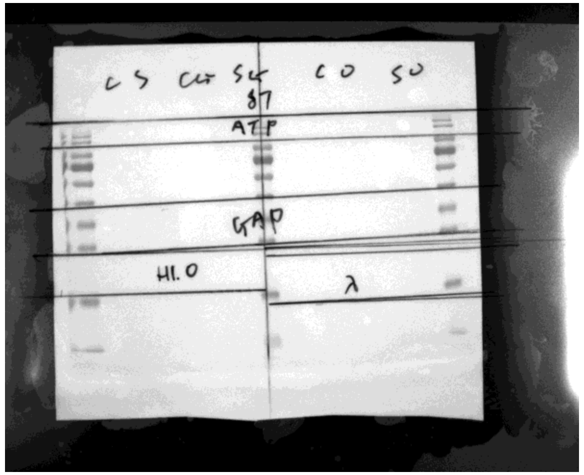

H1.0

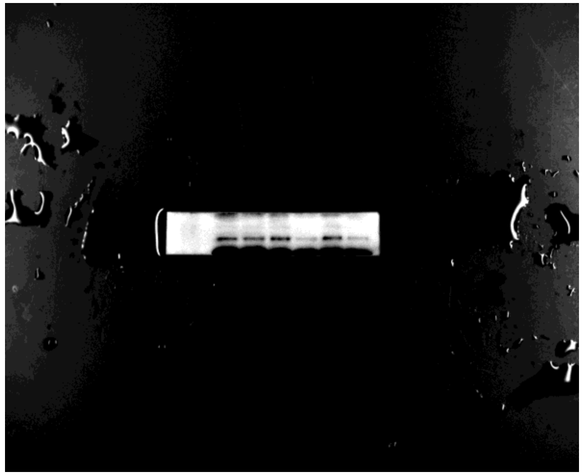

GAPDH

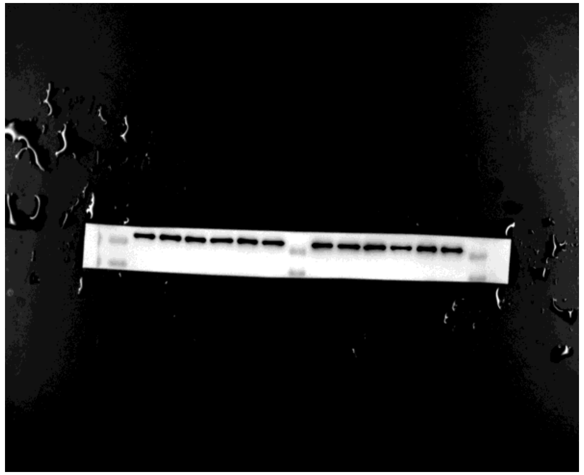

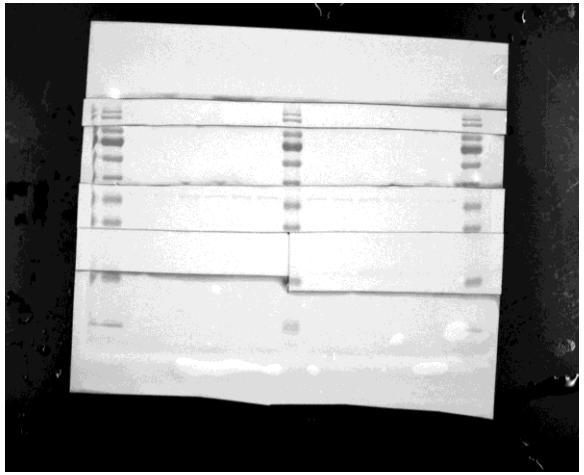

FS2G

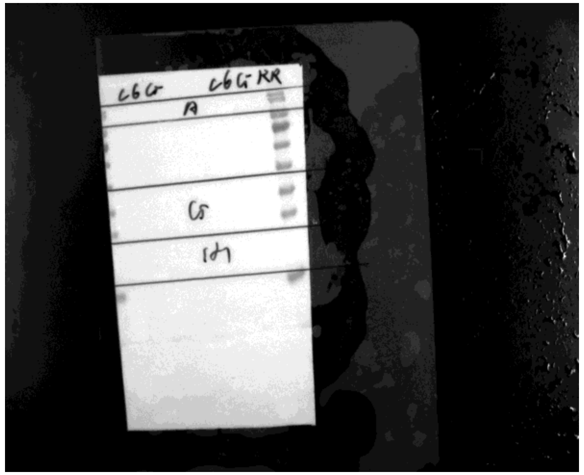

ATP2B4

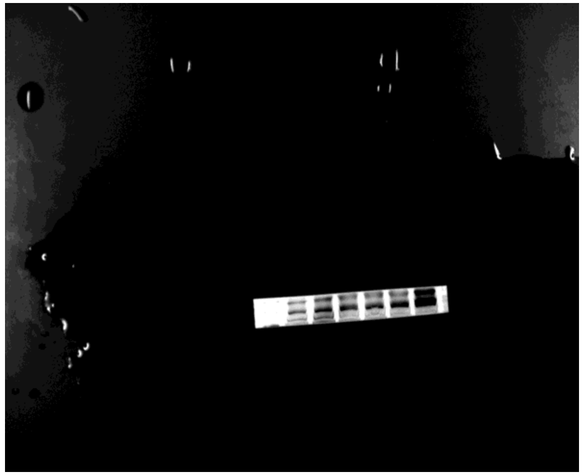

GAPDH

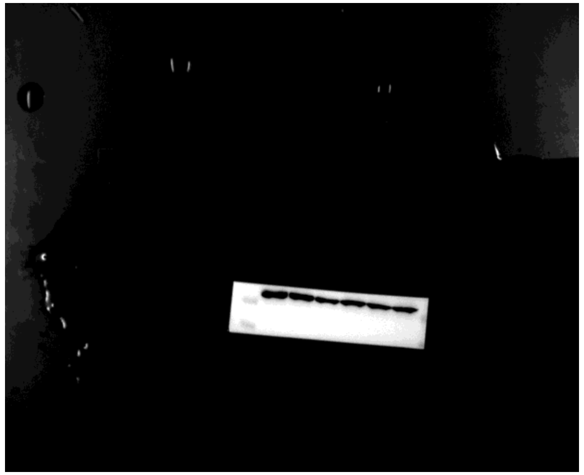

H1.0

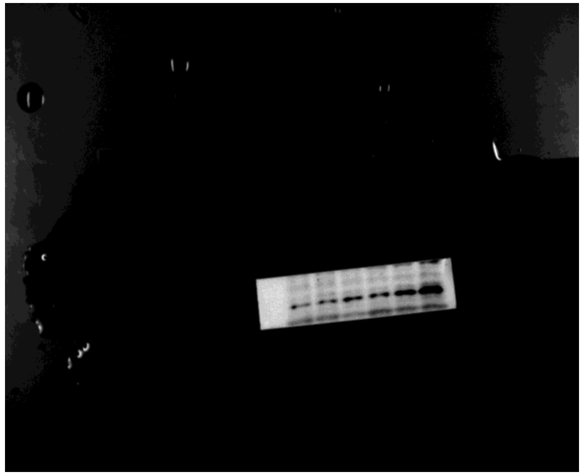

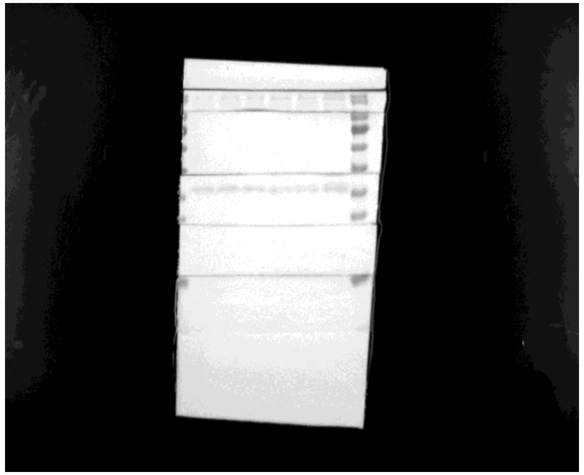

FS3B

ATP2B4 H3

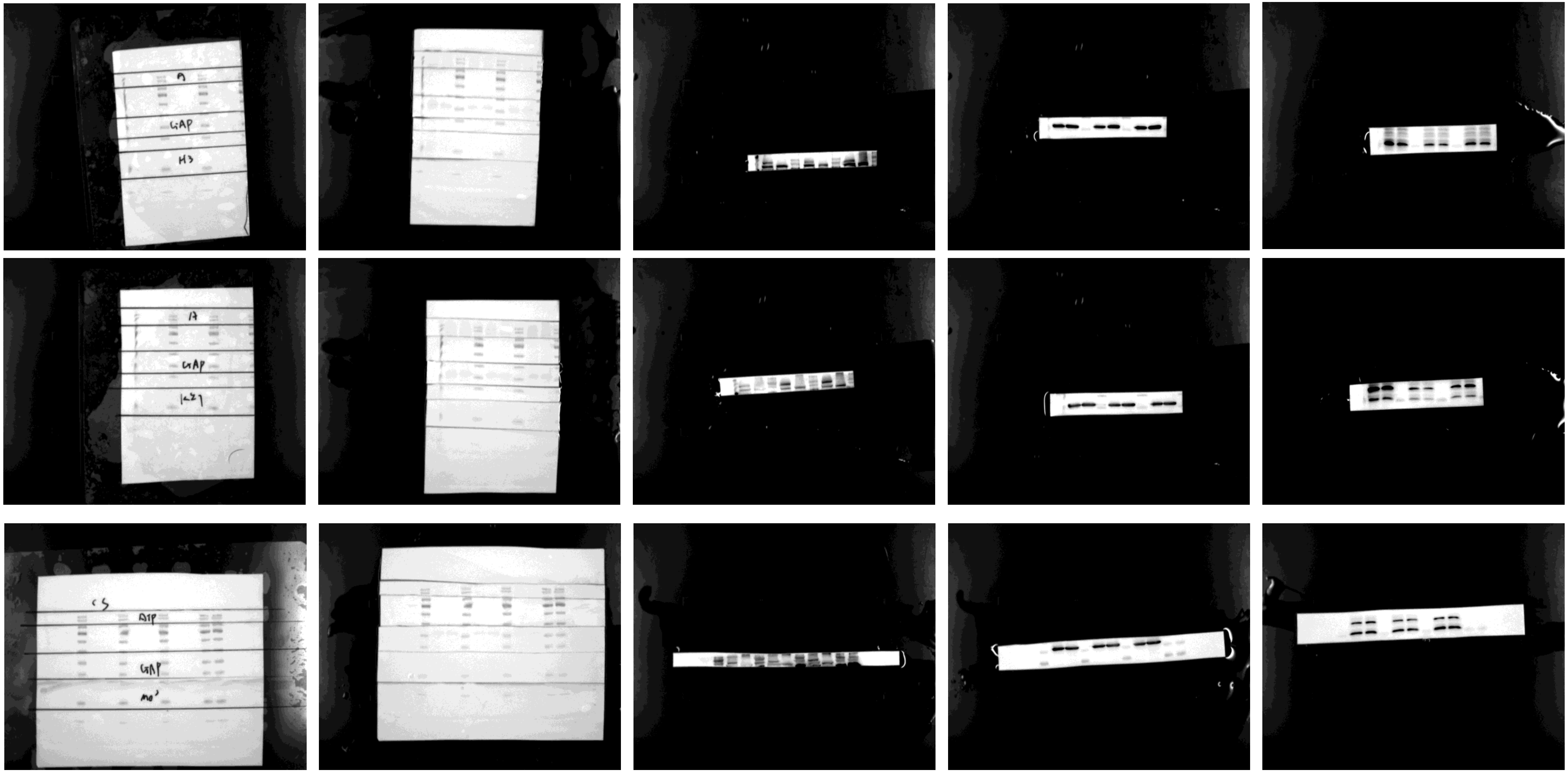

H3

K27

K9

FS3D

ATP2B4 ELAVL1 H3

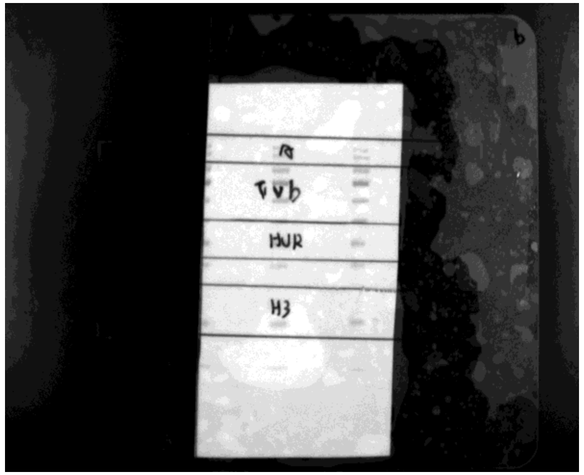
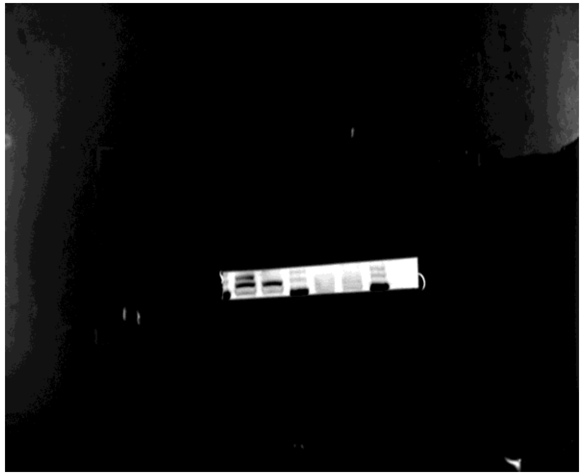
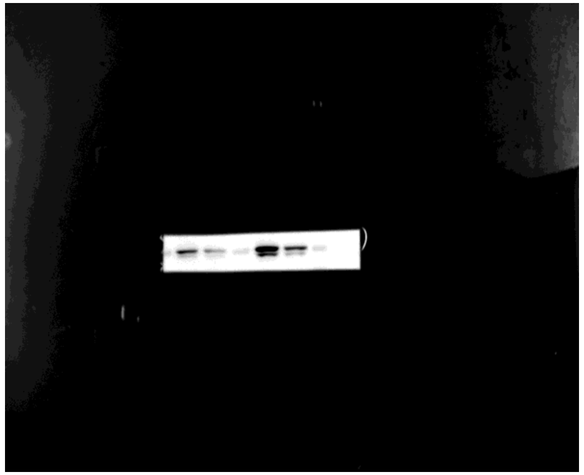
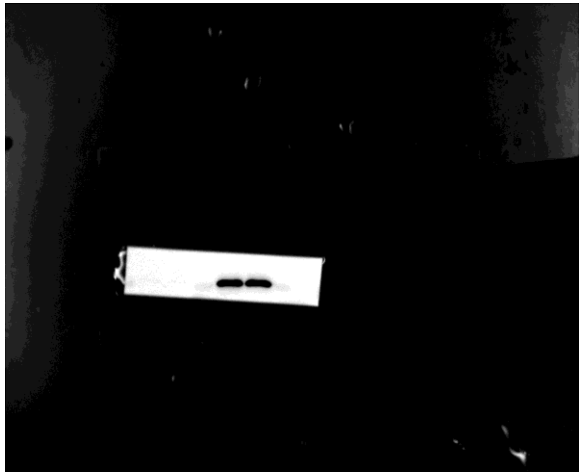
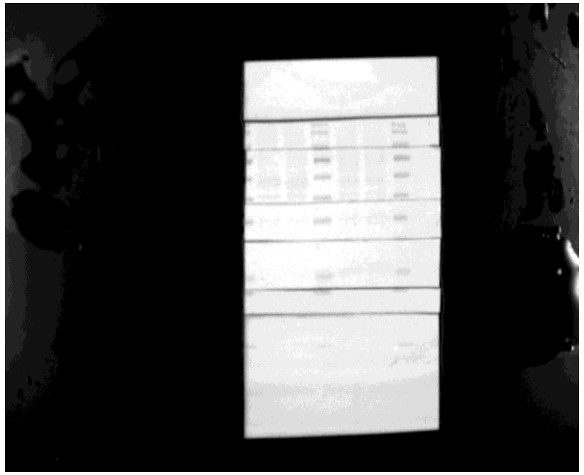
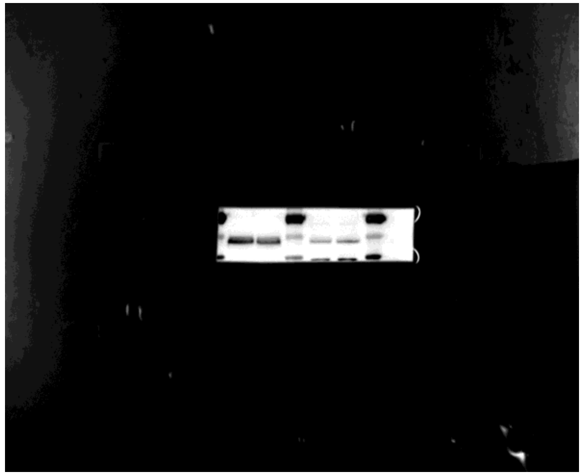

Tublin

FS3E

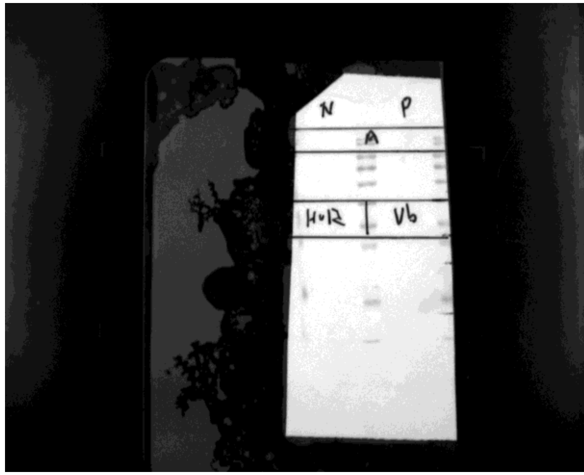

ATP2B4

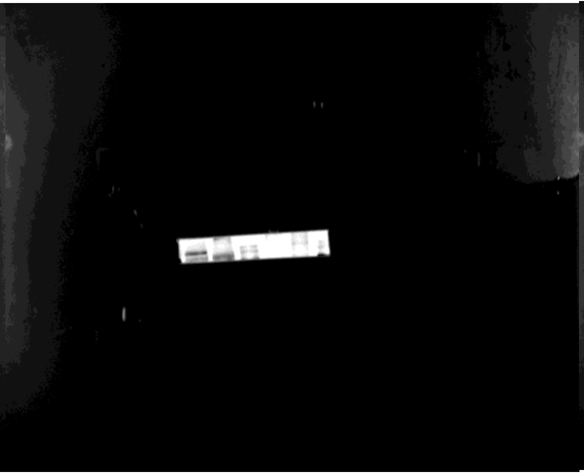

ELAVL1

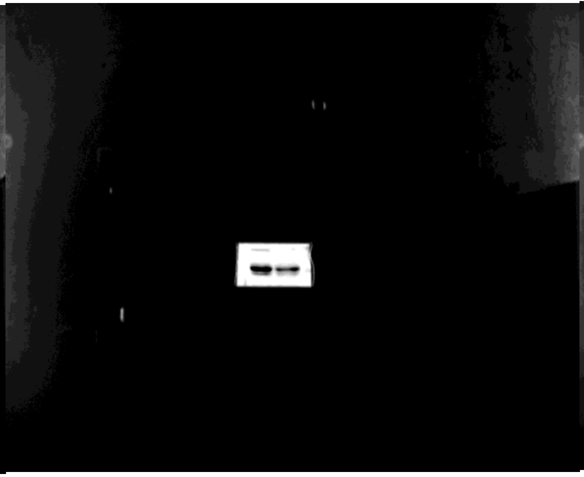

Ubiquitin

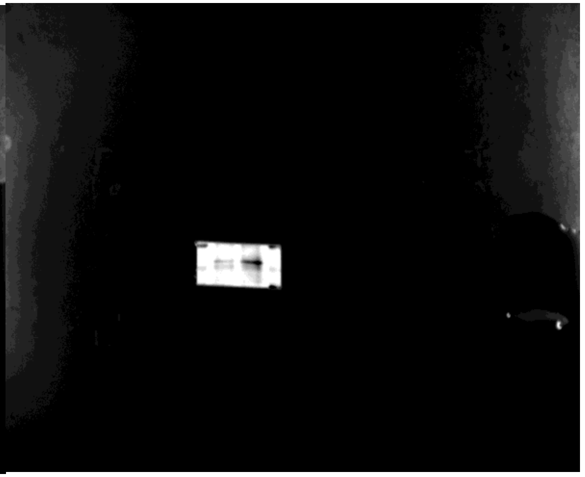

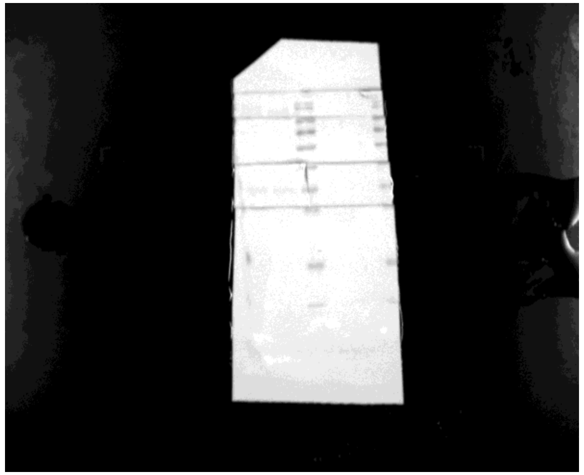

FS4C

H1.0 GAPDH

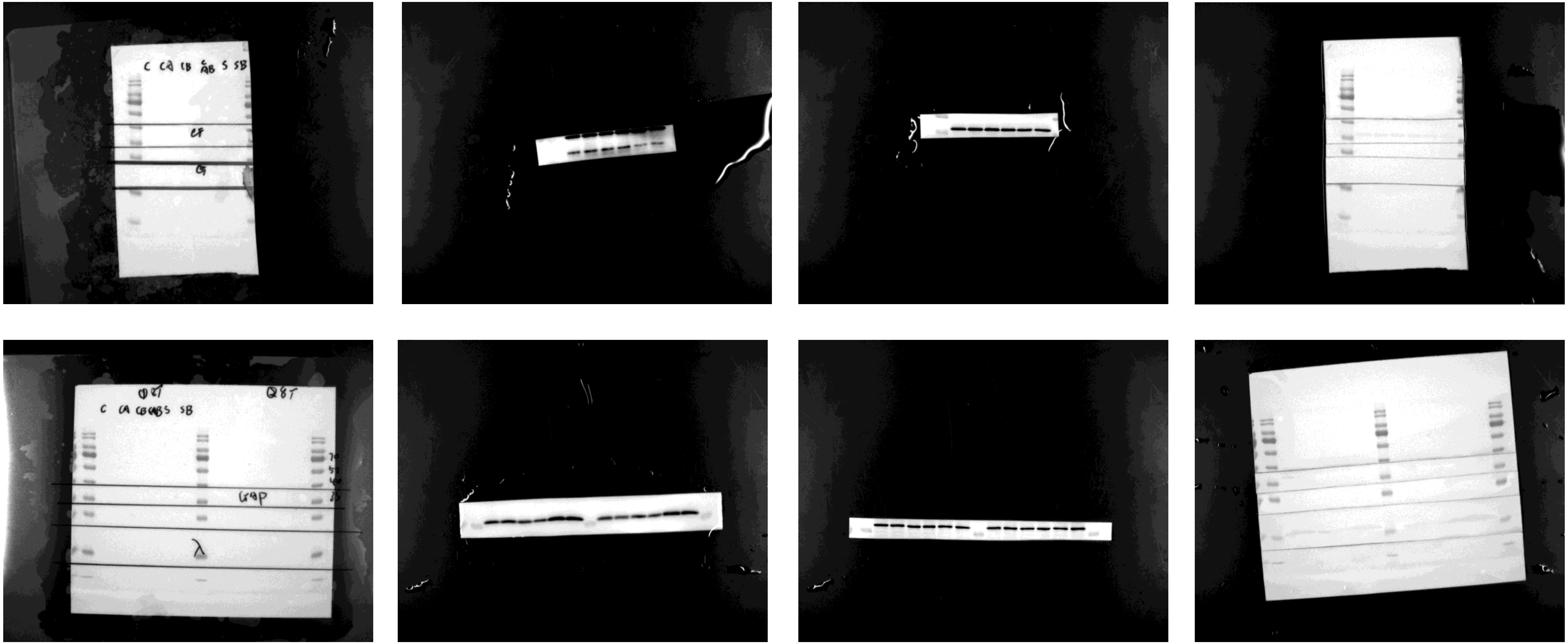

Gama-H2AX GAPDH

FS4E

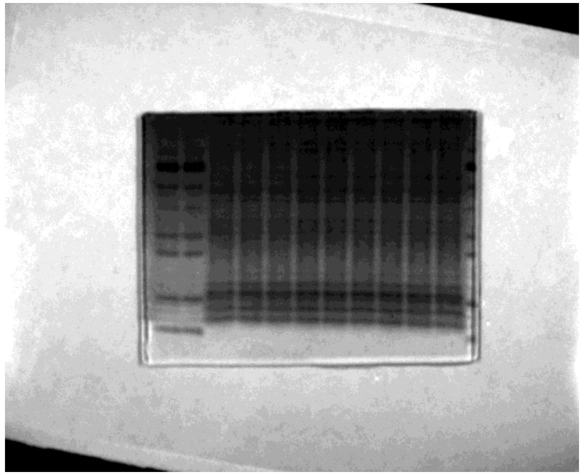

coomassie blue staining

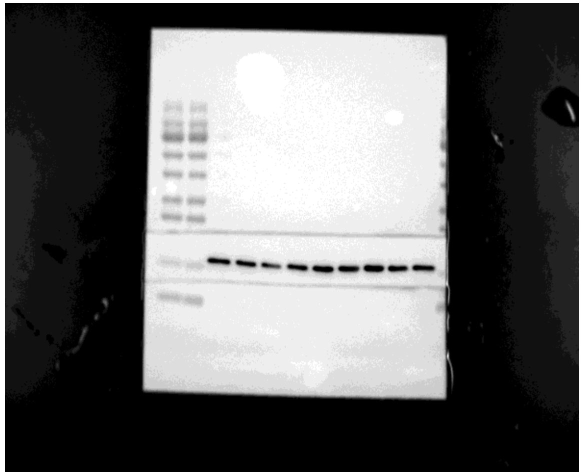

H3

FS4F

ATP2B4

ELAVL1

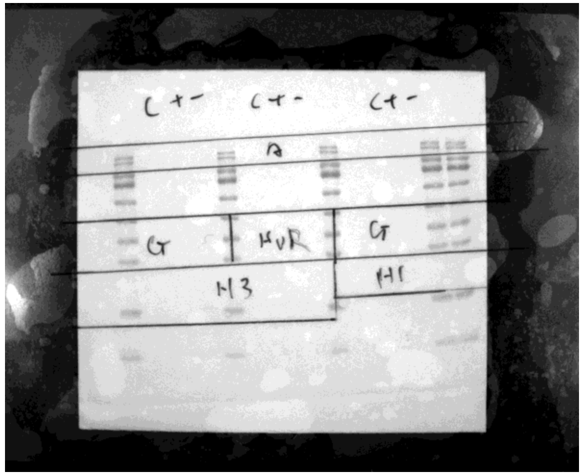

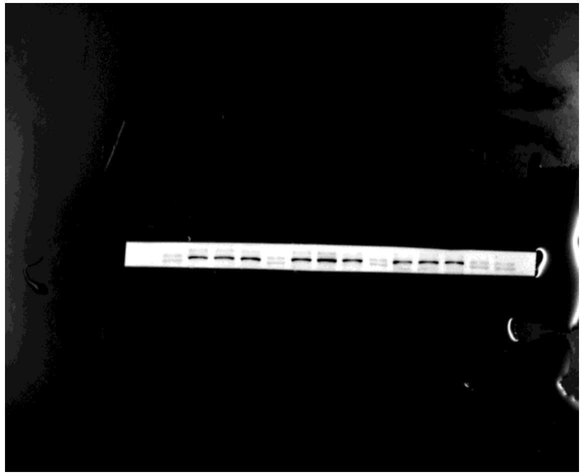

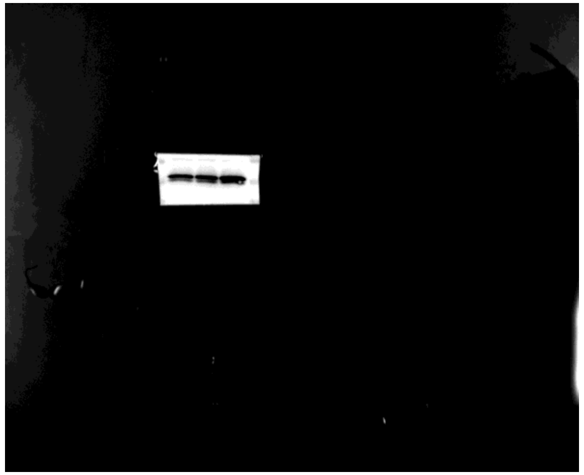

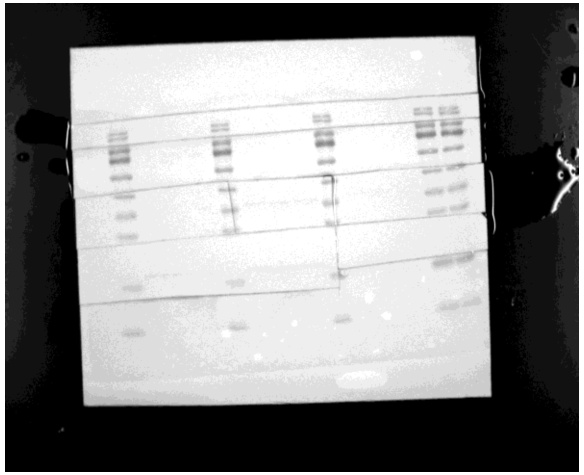

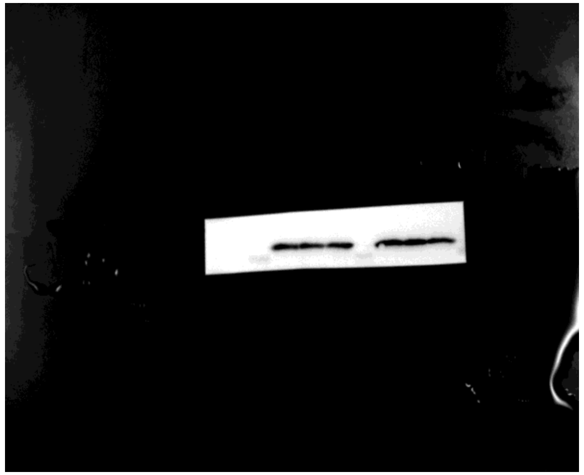

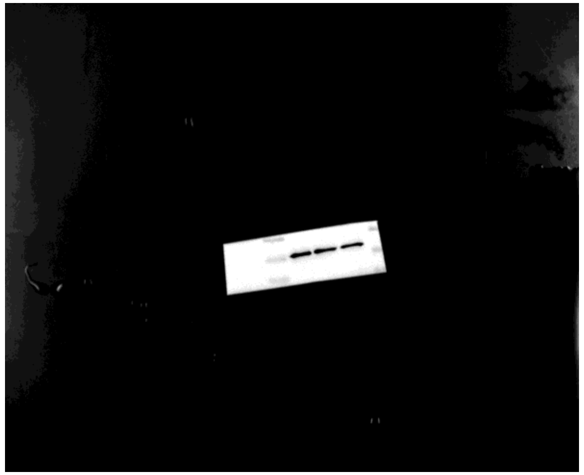

H3

GAPDH

FS4I

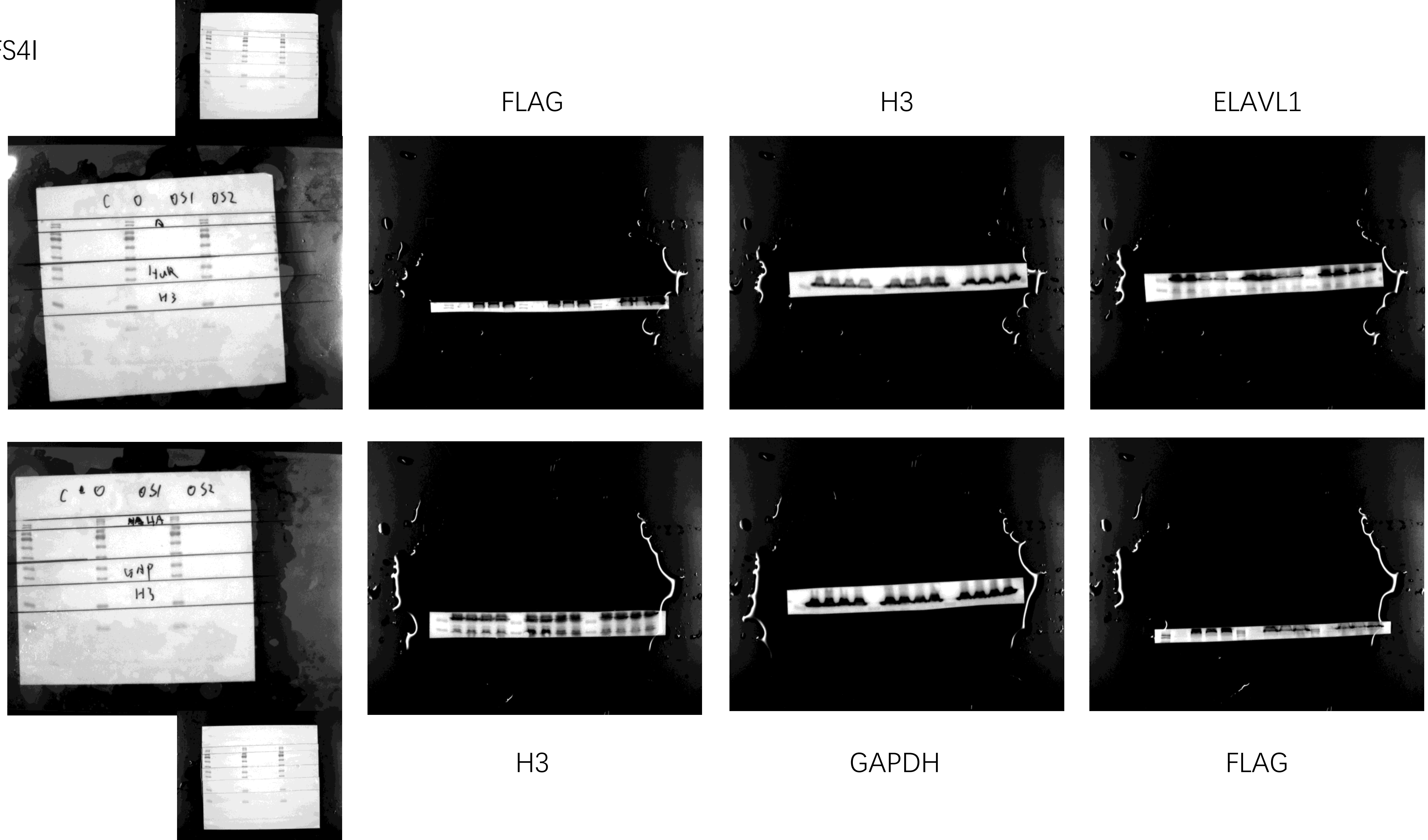

Supplement: Supplementary file 8 — original WB [file 41420_2026_3142_MOESM8_ESM.pdf]
